# Global, regional, and national burden of intracerebral hemorrhage and attributable risk factors in youths and young adults, 1990–2021: a statistical analysis of incidence, mortality, and DALYs

**DOI:** 10.3389/fneur.2025.1594166

**Published:** 2025-09-09

**Authors:** Bing Wu, Changbao Huang, Qian Li, Yuanyuan Rui

**Affiliations:** ^1^Department of Emergency, The Second People’s Hospital of Lu’an City, Lu’an, China; ^2^Department of Emergency, The First Affiliated Hospital of Wannan Medical College, Wuhu, China

**Keywords:** intracerebral hemorrhage, global burden of disease, incidence, mortality, sociodemographic index

## Abstract

**Background:**

Intracerebral hemorrhage (ICH) remains a leading contributor to mortality and long-term disability worldwide. This study aims to report global trends in the incidence, mortality and disability-adjusted life years (DALYs) of ICH among youths and young adults from 1990 to 2021.

**Methods:**

This study analyzed ICH incidence, mortality, and DALYs in youth and young adults aged 15–39 years using data from the Global Burden of Disease (GBD) database. Rates for incidence, mortality, and DALYs were calculated per 100,000 population with 95% uncertainty intervals (UIs). Data from 204 countries and territories were stratified by age, sex, and location. Temporal trends were assessed through Joinpoint regression models to compute annual percent change (APC) and log-transformed linear regression models to estimate the average annual percentage change (EAPC).

**Results:**

Globally, the incidence of ICH among youths and young adults in 2021 was 246938.25 (95% UI, 192258.36–303133.32), with 85038.37 deaths (95% UI, 76818.49–93855.53), and 5385247.12 DALYs (95% UI, 4884623.97–5910984.71). From 1990 to 2021, the incidence decreased by −0.4% (95% UI, −6.14 to 5.54%), mortality by −4.62% (95% UI, −14.24 to 6.74%), and DALYs by −5.08% (95% UI, −13.98 to 5.38%). The incidence rate declined from 11.31 (95% UI, 8.56, 14.27) per 100,000 individuals in 1990 to 8.30 (95% UI, 6.46, 10.19) per 100,000 individuals in 2021, an overall decrease of −26.62% (95% UI, −30.85 to −22.24). Among the five Sociodemographic Index (SDI) regions, the highest EAPCs were observed in the high SDI regions for incidence (−1.63%; 95% CI, −1.74 to −1.52), mortality (−1.79%; 95% CI, −2.01 to −1.58), and DALYs (−1.67%; 95% UI, −1.84 to −1.50). At the national level, China had the highest number of ICH cases globally in 2021, with 49364.51 cases (95% UI, 37242.00–62918.59). The main risk factors for ICH-related mortality and DALYs globally are hypertension, air pollution, and tobacco use.

**Conclusion:**

The global incidence, mortality, and DALYs of ICH among youths and young adults are generally on a declining trend. The main risk factors are hypertension, air pollution, and tobacco use. A thorough understanding of the epidemiology of ICH in youths and young adults is crucial for developing timely and effective intervention measures.

## Introduction

Intracerebral hemorrhage (ICH) remains a leading contributor to mortality and long-term disability in adults worldwide, particularly in low- and middle-income nations, where it represents a significant public health challenge (1–3). Despite the lower incidence of hemorrhagic stroke compared to ischemic stroke, ICH is associated with higher mortality and more extensive long-term disability (4). This results in a disproportionate social and economic burden, owing to the substantial costs of long-term care, rehabilitation, and lost productivity. Consequently, ICH represents a critical issue for global health that requires urgent attention.

Although existing research has explored the global impact of ICH (5), there is a lack of studies focusing on the 15–39 age group. The 15–39 age group is a crucial stage in an individual’s life, as it encompasses key milestones such as educational attainment, career development, and the establishment of social relationships. Our study aims to provide the most up-to-date and comprehensive epidemiological analysis of ICH in youths and young adults by utilizing the latest Global Burden of Disease (GBD) 2021 dataset and employing advanced statistical methods such as Estimated Annual Percentage Change (EAPC). These methods enable us to assess temporal trends and offer a forward-looking perspective on the ICH burden in this age group.

## Methods

### Overview and methods

The GBD database is one of the most comprehensive and systematic epidemiological datasets worldwide. Managed by the Institute for Health Metrics and Evaluation (IHME) at the University of Washington, its primary goal is to quantify health losses due to various diseases, injuries, and risk factors (6). The GBD framework enables comparisons of disease burden across countries and regions. Key GBD metrics include prevalence, incidence, mortality, and disability-adjusted life years (DALYs). DALYs are calculated as the sum of Years of Life Lost (YLL) due to premature death and Years Lived with Disability (YLD). The specific formula are as follows (Equations 1, 2):


(1) $$\begin{array}{l} \mathrm{YLL}=\text{number of death}\times \text{standard life}\;\\ \text{expectancy}\;\mathrm{at}\;\text{the}\;\mathrm{age}\;\text{of death} \end{array}$$

(2) YLD=prevalence of the condition×disability weight

Disability weight ranges from 0 (perfect health) to 1 (death) and is assigned based on expert consensus. This methodological framework ensures a scientifically rigorous understanding of global disease burden. This study analyzes GBD data on ICH, including incidence, mortality, and DALYs. To enhance clarity in risk factor analysis, second-level risk factors were selected for classification. Data were obtained from the GBD database^1^ and downloaded on February 13, 2025 (7). Analyses were stratified by sex, age (15–19, 20–24, 25–29, 30–34, and 35–39 years), region, and year. The dataset contains no personally identifiable information.

### Sociodemographic index

Sociodemographic index (SDI) is an indicator used to assess the level of social development in a country or region, based on economic income, education level, and fertility rate (8). The SDI value ranges from 0 to 1, with 0 representing the lowest economic income and education level, and the highest fertility rate; 1 represents the highest economic income and education level, and the lowest fertility rate. In the GBD database, 204 countries and regions are classified into five SDI levels: low, low-middle, middle, middle-high, and high. This indicator helps examine the impact of socio-economic factors and regional differences on ICH disease burden.

### Statistical analysis

Annual Percentage Change (APC) and its 95% confidence interval (CI) were calculated using the joinpoint regression model to evaluate internal trends for each individual time period (9). EAPC and its 95% CI were calculated using a log-transformed linear regression model to analyze temporal trends in the incidence rate, death rate, and DALY rate of ICH from 1990 to 2021 (10). The specific calculation method is as follows (Equations 3, 4):


(3) y=α+βxε

(4) EAPC=100×(exp(β)−1)

Where x represents years, y is the natural logarithm of the rate (such as incidence rate), α is the intercept, β is the slope, and ε is the random error. EAPC is particularly valuable for assessing long-term trends because it reveals whether the burden is increasing or decreasing over time, unaffected by short-term fluctuations. If the lower bound of the 95% CI of EAPC is greater than 0, it indicates an increasing trend for the corresponding indicator. If the upper bound of the 95% CI of EAPC is less than 0, it indicates a decreasing trend for the corresponding indicator. If the 95% CI includes 0, it indicates that there is no statistically significant trend in the change of the indicator. Our study also used Percentage Change (PC) to reflect the changes in indicators from 1990 to 2021. The specific calculation methods are as follows (Equations 5, 6):


(5) Cases change=(2021cases−1990cases)/1990cases

(6) Rate change=(2021rate−1990rate)/1990rate

Fitting curves were used to analyze the relationship between disease burden and SDI. The analysis in this study was performed using R language and Joinpoint Software 5.1.0.0 (National Cancer Institute, United States).

## Results

### Global burden trends

#### Incidence

**Figure 1:**
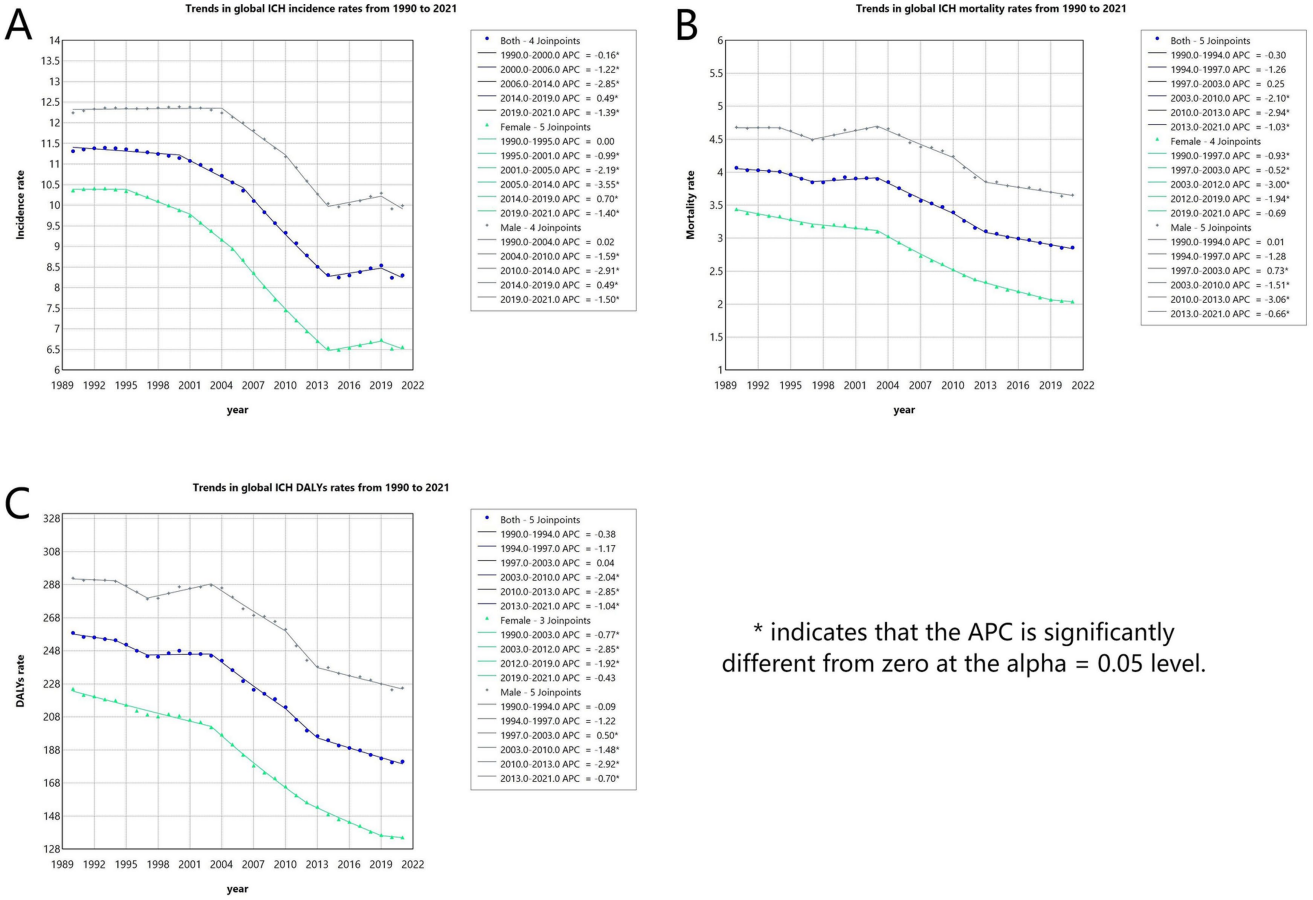
Annual percent change (APC) and trends in global ICH incidence, mortality and disability-adjusted life years (DALYs) among youths and young adults from 1990 to 2021. **(A)** Incidence rate. **(B)** Mortality rate. **(C)** DALYs rate.

**Figure 2:**
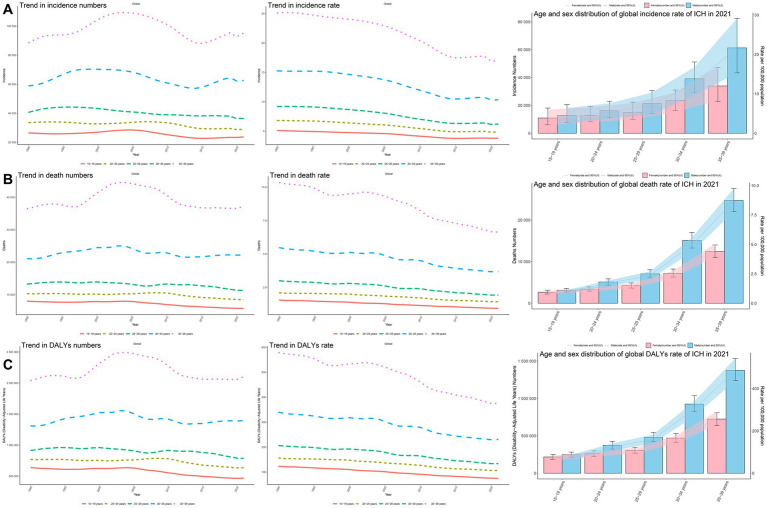
Trends in incidence, mortality, and disability-adjusted life years (DALYs) of ICH among youths and young adults by age and sex, 1990–2021. **(A)** Incidence cases and rates. **(B)** Mortality cases and rates. **(C)** DALYs cases and rates.

**Figure 3:**
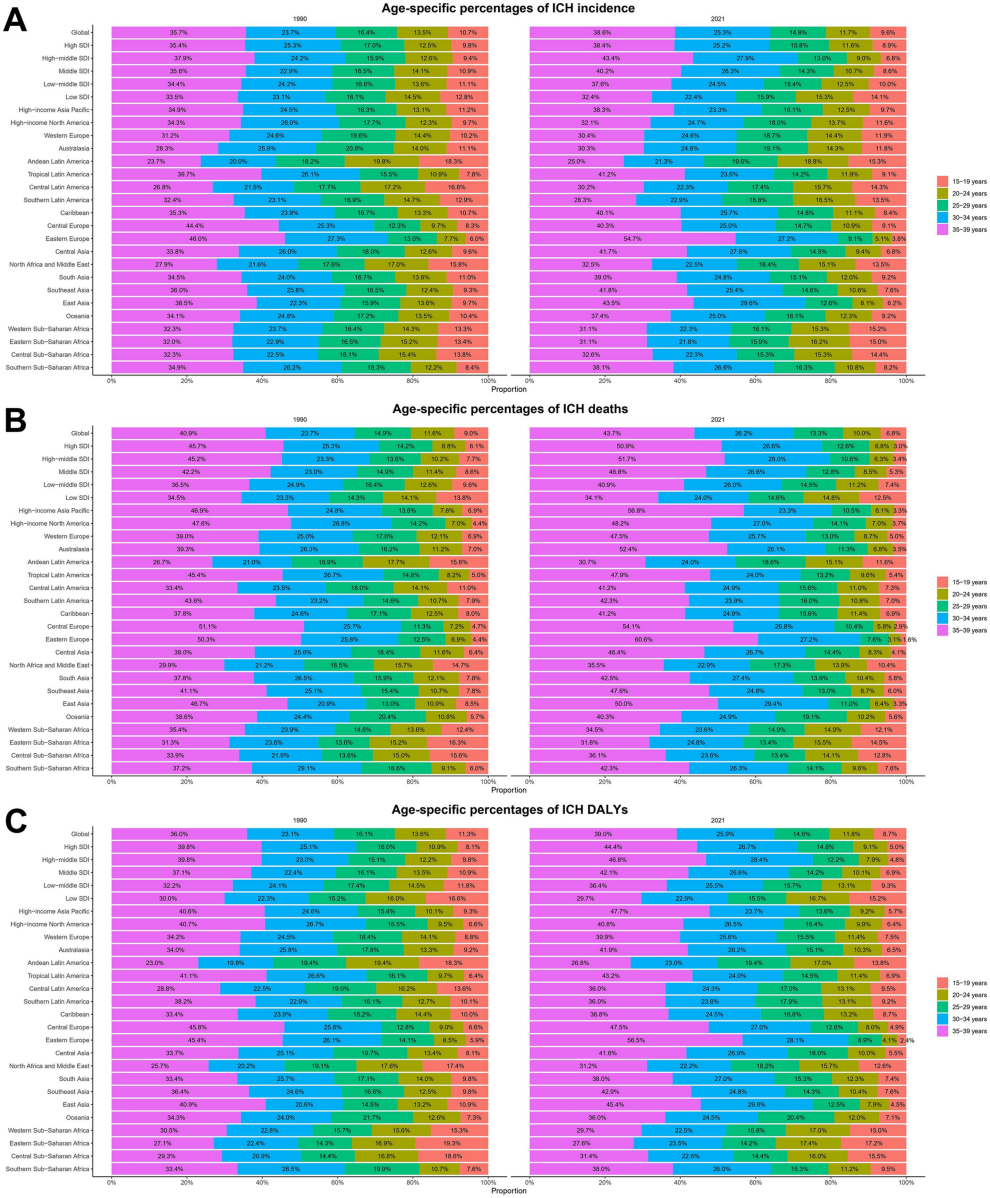
Age-specific percentages of ICH incidence, mortality, and disability-adjusted life years (DALYs) among youths and young adults in 1990 and 2021. **(A)** Incidence. **(B)** Deaths. **(C)** DALYs.

The global incidence rate of ICH in youths and young adults has shown a declining trend. However, a slight increase was observed from 2014 to 2019. The most significant decline occurred from 2006 to 2014, with an APC of −2.85% (95% CI, −2.98 to −2.72%) (Figure 1A). The lowest incidence rate was recorded in 2020 at 8.24 (95% UI, 10.10–6.43) per 100,000 population (Figure 1A). Globally, the number of ICH cases decreased marginally from 247927.77 (95% UI, 187560.36–312679.06) in 1990 to 246938.25 (95% UI, 192258.36–303133.32) in 2021, reflecting a − 0.4% change (95% UI, −6.14 to 5.54%). The incidence rate declined from 11.31 (95% UI, 8.56–14.27) per 100,000 in 1990 to 8.30 (95% UI, 6.46–10.19) per 100,000 in 2021, a reduction of −26.62% (95% UI, −30.85 to −22.24%), with an EAPC of −1.31% (95% CI, −1.46 to −1.15%) (Table 1). Among individuals aged 15–29 years, the incidence decreased across all subgroups, with the most significant decline observed in the 20–24 age group (−13.56%). Conversely, the incidence increased in the 30–39 age group, particularly in the 35–39 age group (+7.71%) (Table 1; Figure 2A). The highest incidence rate was observed in the 35–39 age group, with its proportion of total cases rising from 35.7% in 1990 to 38.6% in 2021. The incidence rate in this group declined from 25.11 (95% UI, 16.75–23.06) per 100,000 in 1990 to 16.98 (95% UI, 11.99–23.06) per 100,000 in 2021, a reduction of −32.35% (95% UI, −38.22 to −24.31%), with an EAPC of −1.48% (95% CI, −1.64 to −1.32%) (Table 1; Figures 2A, 3A). The lowest incidence rate was found in the 15–19 age group, with its proportion of total cases decreasing from 10.7% in 1990 to 9.6% in 2021. The incidence rate declined from 5.10 (95% UI, 2.95–8.33) per 100,000 in 1990 to 3.81 (95% UI, 2.19–6.09) per 100,000 in 2021, representing a − 25.40% reduction (95% UI, −31.87 to −19.21%), with an EAPC of −1.14% (95% CI, −1.24 to −1.05%) (Table 1; Figures 2A, 3A). Regarding sex differences, males consistently had higher incidence rates than females, with the largest gap observed in the 35–39 age group (Figure 2A).

**Table 1:** Incidence of ICH between 1990 and 2021 at the global and regional level.

Location	Rate per 100,000 (95% UI)						
	1990		2021		1990–2021		
	Incident cases	Incident rate	Incident cases	Incident rate	Cases change^b^	Rate change^b^	EAPC^a^
Global	247927.77 (187560.36, 312679.06)	11.31 (8.56, 14.27)	246938.25 (192258.36, 303133.32)	8.30 (6.46, 10.19)	−0.40 (−6.14, 5.54)	−26.62 (−30.85, −22.24)	−1.31 (−1.46, −1.15)
Sex							
Male	135700.74 (102954.62, 169276.54)	12.24 (9.29, 15.27)	150829.75 (118258.90, 183213.61)	9.99 (7.83, 12.14)	11.15 (4.11, 18.44)	−18.39 (−23.56, −13.04)	−0.86 (−1.00, −0.71)
Female	112227.04 (83673.39, 144329.29)	10.36 (7.72, 13.32)	96108.50 (74145.12, 121543.33)	6.56 (5.06, 8.30)	−14.36 (−19.72, −9.37)	−36.68 (−40.64, −32.99)	−1.93 (−2.12, −1.73)
Age (years)							
15–19	26507.24 (15320.75, 43280.52)	5.10 (2.95, 8.33)	23755.97 (13639.78, 38006.82)	3.81 (2.19, 6.09)	−10.38 (−18.15, −2.95)	−25.40 (−31.87, −19.21)	−1.14 (−1.24, −1.05)
20–24	33555.11 (22477.58, 48724.48)	6.82 (4.57, 9.90)	29004.66 (19627.13, 41945.94)	4.86 (3.29, 7.02)	−13.56 (−19.49, −7.94)	−28.77 (−33.66, −24.14)	−1.33 (−1.43, −1.22)
25–29	40617.49 (26420.10, 60603.80)	9.18 (5.97, 13.69)	36424.86 (24412.36, 51914.12)	6.19 (4.15, 8.82)	−10.32 (−16.73, −2.87)	−32.53 (−37.36, −26.93)	−1.57 (−1.70, −1.44)
30–34	58809.23 (42837.53, 76440.18)	15.26 (11.11, 19.83)	62493.04 (46825.40, 80982.37)	10.34 (7.75, 13.40)	6.26 (−0.54, 13.35)	−32.25 (−36.59, −27.73)	−1.55 (−1.71, −1.39)
35–39	88438.70 (59013.36, 121778.76)	25.11 (16.75, 34.57)	95259.71 (67256.74, 129334.13)	16.98 (11.99, 23.06)	7.71 (−1.63, 20.52)	−32.35 (−38.22, −24.31)	−1.48 (−1.64, −1.32)
SDI							
High SDI	22538.60 (16836.70, 29326.75)	6.50 (4.85, 8.45)	15186.60 (11068.24, 20195.96)	4.30 (3.13, 5.72)	−32.62 (−36.69, −29.01)	−33.82 (−37.82, −30.27)	−1.63 (−1.74, −1.52)
High-middle SDI	56362.72 (42487.31, 71190.09)	12.45 (9.39, 15.73)	38254.33 (29259.18, 47724.88)	8.69 (6.65, 10.84)	−32.13 (−36.48, −27.03)	−30.24 (−34.71, −24.99)	−1.60 (−1.81, −1.38)
Middle SDI	92351.36 (68992.13, 117695.75)	12.27 (9.17, 15.64)	82822.00 (63923.85, 102089.43)	8.93 (6.89, 11.01)	−10.32 (−16.45, −3.52)	−27.23 (−32.20, −21.71)	−1.33 (−1.51, −1.15)
Low-middle SDI	51738.17 (40001.53, 64661.47)	11.41 (8.82, 14.26)	71091.25 (55844.66, 87635.65)	8.86 (6.96, 10.92)	37.41 (29.19, 45.14)	−22.37 (−27.01, −18.00)	−1.04 (−1.18, −0.91)
Low SDI	24722.19 (19704.22, 30179.75)	13.41 (10.69, 16.37)	39396.01 (31976.32, 47524.25)	8.77 (7.12, 10.58)	59.35 (50.91, 68.57)	−34.60 (−38.06, −30.81)	−1.58 (−1.68, −1.48)
Regions							
Andean Latin America	1731.82 (1419.98, 2083.89)	11.20 (9.18, 13.48)	1588.64 (1261.47, 1945.78)	5.87 (4.66, 7.19)	−8.27 (−15.61, −0.84)	−47.62 (−51.81, −43.37)	−2.28 (−2.40, −2.17)
Australasia	291.89 (210.62, 397.02)	3.58 (2.58, 4.87)	227.25 (145.97, 342.04)	2.17 (1.39, 3.27)	−22.14 (−36.33, −9.06)	−39.37 (−50.42, −29.18)	−1.92 (−2.18, −1.66)
Caribbean	1488.19 (1226.33, 1756.91)	10.01 (8.25, 11.82)	1534.14 (1278.37, 1799.57)	8.43 (7.02, 9.89)	3.09 (−2.54, 9.08)	−15.82 (−20.41, −10.93)	−0.82 (−0.92, −0.71)
Central Asia	3842.68 (3220.46, 4519.27)	13.50 (11.32, 15.88)	3664.08 (3050.67, 4292.55)	9.80 (8.16, 11.48)	−4.65 (−9.68, 0.67)	−27.43 (−31.26, −23.38)	−1.40 (−1.64, −1.16)
Central Europe	4504.60 (3647.01, 5338.63)	9.62 (7.78, 11.40)	1583.98 (1197.31, 2013.15)	4.52 (3.42, 5.75)	−64.84 (−67.79, −61.68)	−52.96 (−56.91, −48.74)	−2.80 (−2.97, −2.62)
Central Latin America	5104.01 (3906.35, 6464.42)	7.48 (5.72, 9.47)	4517.60 (3394.61, 5857.38)	4.47 (3.36, 5.79)	−11.49 (−17.55, −6.13)	−40.27 (−44.36, −36.65)	−1.98 (−2.15, −1.81)
Central Sub-Saharan Africa	2717.10 (2184.21, 3285.75)	13.09 (10.52, 15.83)	4990.16 (4075.14, 5938.70)	9.22 (7.53, 10.98)	83.66 (69.95, 98.01)	−29.51 (−34.77, −24.01)	−1.30 (−1.36, −1.23)
East Asia	76992.83 (56555.50, 99688.34)	13.61 (10.00, 17.62)	51831.73 (39374.82, 65720.46)	10.82 (8.22, 13.72)	−32.68 (−38.73, −25.40)	−20.50 (−27.64, −11.91)	−1.18 (−1.45, −0.90)
Eastern Europe	7662.06 (5661.74, 10118.29)	8.93 (6.60, 11.80)	6327.32 (4775.40, 8151.36)	9.56 (7.22, 12.32)	−17.42 (−25.29, −7.67)	7.03 (−3.16, 19.68)	−0.02 (−0.25, 0.21)
Eastern Sub-Saharan Africa	12379.51 (10013.08, 14861.85)	17.46 (14.12, 20.96)	16866.04 (13881.23, 20147.65)	9.63 (7.92, 11.50)	36.24 (28.80, 44.32)	−44.87 (−47.88, −41.60)	−2.20 (−2.31, −2.08)
High-income Asia Pacific	6976.79 (5330.17, 8795.03)	10.34 (7.90, 13.03)	2686.64 (1880.07, 3689.88)	5.32 (3.72, 7.30)	−61.49 (−67.03, −57.06)	−48.57 (−55.97, −42.66)	−2.67 (−2.85, −2.49)
High-income North America	4729.48 (3130.94, 6787.05)	4.17 (2.76, 5.99)	3984.32 (2723.36, 5533.36)	3.23 (2.21, 4.49)	−15.76 (−22.23, −7.46)	−22.50 (−28.46, −14.87)	−1.02 (−1.11, −0.93)
North Africa and Middle East	15252.67 (12387.83, 18411.00)	11.40 (9.26, 13.76)	18161.06 (14790.05, 21850.06)	7.14 (5.82, 8.59)	19.07 (13.81, 24.03)	−37.33 (−40.10, −34.72)	−1.80 (−1.91, −1.69)
Oceania	327.82 (265.17, 392.18)	12.34 (9.98, 14.76)	550.72 (456.34, 652.28)	9.77 (8.10, 11.58)	67.99 (58.87, 79.39)	−20.80 (−25.10, −15.42)	−0.89 (−1.01, −0.76)
South Asia	40473.45 (29875.41, 52445.66)	9.38 (6.92, 12.15)	60390.59 (45728.19, 75867.96)	7.64 (5.78, 9.59)	49.21 (38.48, 60.63)	−18.57 (−24.43, −12.34)	−0.87 (−1.00, −0.75)
Southeast Asia	32943.21 (25604.58, 40700.35)	16.72 (13.00, 20.66)	38204.61 (30528.44, 45946.57)	13.78 (11.01, 16.57)	15.97 (8.24, 23.76)	−17.62 (−23.11, −12.08)	−0.78 (−0.95, −0.61)
Southern Latin America	2623.88 (2114.94, 3161.15)	13.75 (11.09, 16.57)	1743.55 (1326.43, 2216.34)	6.76 (5.14, 8.59)	−33.55 (−41.31, −25.15)	−50.85 (−56.59, −44.64)	−2.74 (−2.98, −2.50)
Southern Sub-Saharan Africa	2891.30 (2160.66, 3658.43)	13.38 (10.00, 16.93)	2494.99 (1952.85, 3056.68)	7.33 (5.74, 8.98)	−13.71 (−21.14, −5.62)	−45.20 (−49.92, −40.06)	−2.64 (−3.01, −2.26)
Tropical Latin America	8808.30 (6389.32, 11555.16)	13.70 (9.93, 17.97)	4578.26 (3398.26, 5854.43)	5.18 (3.85, 6.63)	−48.02 (−53.10, −42.54)	−62.15 (−65.84, −58.15)	−3.62 (−3.92, −3.33)
Western Europe	7329.91 (5437.04, 9565.20)	5.09 (3.77, 6.64)	3506.21 (2322.09, 5199.01)	2.70 (1.79, 4.01)	−52.17 (−58.83, −45.30)	−46.88 (−54.28, −39.25)	−2.57 (−2.78, −2.37)
Western Sub-Saharan Africa	8856.24 (6947.36, 10982.20)	12.37 (9.71, 15.34)	17506.33 (14154.46, 21223.19)	9.16 (7.40, 11.10)	97.67 (87.08, 108.92)	−26.01 (−29.97, −21.80)	−1.11 (−1.23, −0.98)

EAPC, estimated annual percentage change; SDI, sociodemographic Index; UI, uncertainty interval. ^a^EAPC is expressed as 95% confidence interval. ^b^Change shows the percentage change.

#### Mortality

**Figure 4:**
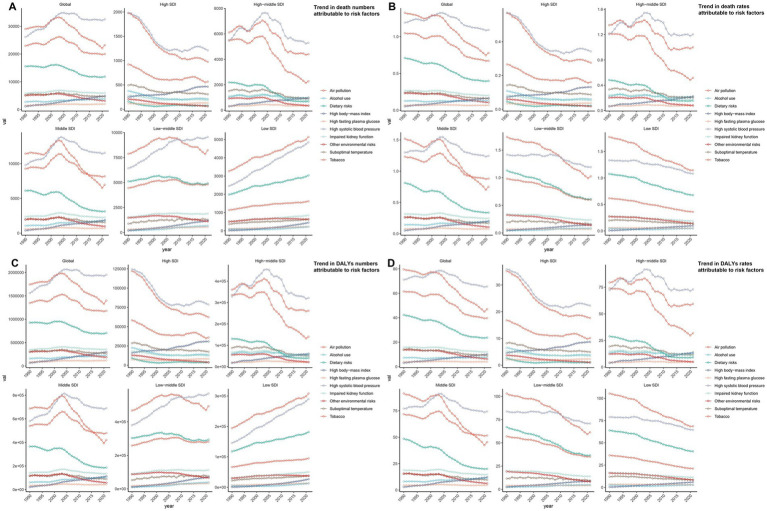
Trends of ICH mortality and disability-adjusted life years (DALYs) among youths and young adults attributable to risk factors from 1990 to 2021, grouping by different Sociodemographic Index (SDI) quintiles. **(A)** Number of deaths. **(B)** Mortality rates. **(C)** Number of DALYs. **(D)** DALYs rates.

The global mortality rate of ICH in youths and young adults has shown a declining trend. The most significant decline occurred from 2010 to 2013, with an APC of −2.94% (95% CI, −4.53 to −1.32%) (Figure 1B). The lowest mortality rate was recorded in 2021 at 2.86 (95% UI, 2.58–3.15) per 100,000 population (Figure 1B). Globally, the number of ICH-related deaths decreased from 89160.94 (95% UI, 81821.53–96337.17) in 1990 to 85038.37 (95% UI, 76818.49–93855.53) in 2021, reflecting a − 4.62% change (95% UI, −14.24 to 6.74%). The mortality rate declined from 4.07 (95% UI, 3.73–4.40) per 100,000 in 1990 to 2.86 (95% UI, 2.58–3.15) per 100,000 in 2021, a reduction of −29.73% (95% UI, −36.81 to −21.36%), with an EAPC of −1.28% (95% CI, −1.42 to −1.13%) (Supplementary Table S1). Air pollution, high systolic blood pressure, and tobacco use remained the leading risk factors for ICH mortality (Figures 4A,B). Among individuals aged 15–29 years, mortality declined across all subgroups, with the most significant reduction observed in the 15–19 age group (−27.89%). Conversely, the mortality increased in the 30–39 age group, particularly in the 30–34 age group (+5.48%) (Supplementary Table S1; Figure 2B). The highest mortality rate was observed in the 35–39 age group, with its proportion of total deaths rising from 40.9% in 1990 to 43.7% in 2021. The mortality rate in this group declined from 10.35 (95% UI, 9.50–11.17) per 100,000 in 1990 to 6.63 (95% UI, 6.03–7.29) per 100,000 in 2021, a reduction of −35.91% (95% UI, −42.65 to −27.78%), with an EAPC of −1.49% (95% CI, −1.65 to −1.33%) (Supplementary Table S1; Figures 2B, 3B). In both 1990 and 2021, air pollution, high systolic blood pressure, and tobacco use were the leading risk factors for mortality in 35–39 age group (Supplementary Figure S1). The lowest mortality rate was recorded in the 15–19 age group, with its proportion of total deaths decreasing from 9.0% in 1990 to 6.8% in 2021. The mortality rate declined from 1.55 (95% UI, 1.39–1.70) per 100,000 in 1990 to 0.93 (95% UI, 0.81–1.04) per 100,000 in 2021, representing a − 39.97% reduction (95% UI, −47.15 to −31.63%), with an EAPC of −1.71% (95% CI, −1.78 to −1.65%) (Supplementary Table S1; Figures 2B, 3B). In 1990 and 2021, suboptimal temperature was a key risk factor for mortality in 15–19 age group, whereas alcohol consumption was identified as a protective factor (Supplementary Figure S1). Regarding sex differences, males consistently exhibited higher mortality rates than females, with the largest gap observed in the 35–39 age group (Figure 2B).

#### DALYs

The global DALY rate of ICH in youths and young adults has shown a declining trend. The most significant decline occurred between 2010 and 2013, with an APC of −2.85% (95% CI, −4.43 to −1.23%) (Figure 1C). The lowest DALY rate was recorded in 2021 at 181.03 (95% UI, 164.20–198.70) per 100,000 population (Figure 1C). Globally, the number of ICH-related DALYs decreased from 5673698.79 (95% UI, 5241479.49–6127466.64) in 1990 to 5385247.12 (95% UI, 4884623.97–5910984.71) in 2021, reflecting a − 5.08% change (95% UI, −13.98 to 5.38%). The DALY rate declined from 258.86 (95% UI, 239.14–279.56) per 100,000 in 1990 to 181.03 (95% UI, 164.20–198.70) per 100,000 in 2021, a reduction of −30.07% (95% UI, −36.62 to −22.36%), with an EAPC of −1.30% (95% CI, −1.43 to −1.16%) (Supplementary Table S2). Air pollution, high systolic blood pressure, and tobacco use remained the leading risk factors for ICH DALYs (Figures 4C,D). Among individuals aged 15–29 years, DALYs decreased across all subgroups, with the most significant reduction observed in the 15–19 age group (−27.03%). Conversely, DALYs increased in the 30–39 age group, particularly in the 30–34 age group (+6.45%) (Supplementary Table S2; Figure 2C). The highest DALY rate was observed in the 35–39 age group, with its proportion of total DALYs increasing from 36.0% in 1990 to 39.0% in 2021. The DALY rate in this group declined from 579.11 (95% UI, 530.06–623.36) per 100,000 in 1990 to 374.67 (95% UI, 341.66–411.04) per 100,000 in 2021, a reduction of −35.30% (95% UI, −41.76 to −27.67%), with an EAPC of −1.47% (95% CI, −1.62 to −1.32%) (Supplementary Table S2; Figures 2C, 3C). In both 1990 and 2021, air pollution, high systolic blood pressure, and tobacco use were the primary risk factors contributing to DALYs in 35–39 age group (Supplementary Figure S2). The lowest DALY rate was recorded in the 15–19 age group, with its proportion of total DALYs declining from 11.3% in 1990 to 8.7% in 2021. The DALY rate decreased from 123.08 (95% UI, 112.06–134.19) per 100,000 in 1990 to 74.77 (95% UI, 65.37–83.79) per 100,000 in 2021, representing a − 39.26% reduction (95% UI, −45.77 to −31.50%), with an EAPC of −1.69% (95% CI, −1.76 to −1.63%) (Supplementary Table S2; Figures 2C, 3C). In 1990 and 2021, suboptimal temperature was a key risk factor for DALYs in this age group, whereas alcohol consumption was identified as a protective factor (Supplementary Figure S2). Regarding sex differences, males consistently exhibited higher DALY rates than females, with the largest gap observed in the 35–39 age group (Figure 2C).

#### Regional trends by SDI

**Figure 5:**
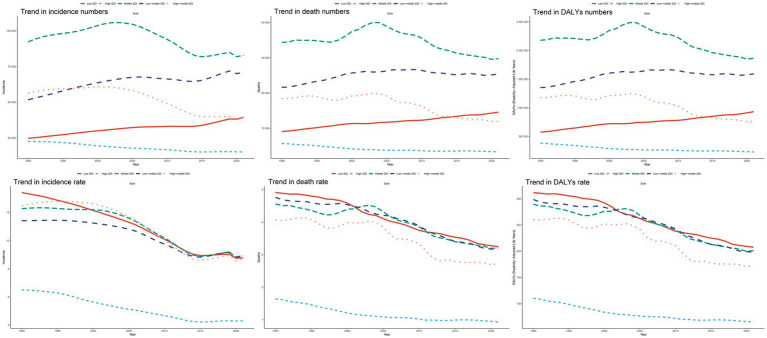
Epidemiological trends in ICH incidence, mortality, and disability-adjusted life years (DALYs) rates among youths and young adults across five Sociodemographic Index (SDI) areas from 1990 to 2021.

Compared to 1990, the incidence, mortality, and DALYs of ICH declined in high, high-middle, and middle SDI regions in 2021, whereas they increased in low-middle and low SDI regions. In 2021, the lowest incidence, mortality, and DALYs were observed in high SDI regions. The most pronounced declines in incidence, mortality, and DALY rate were also seen in high SDI regions, with corresponding EAPCs of −1.63% (95% CI, −1.74 to −1.52), −1.79% (95% CI, −2.01 to −1.58), and −1.67% (95% CI, −1.84 to −1.50), respectively (Table 1; Figure 5; Supplementary Tables S1, S2; Supplementary Figure S5). Among all SDI regions, the slowest decline in incidence rate was observed in low-middle SDI regions, with an EAPC of −1.04% (95% CI, −1.18 to −0.91). Meanwhile, middle SDI regions experienced the slowest reductions in mortality rate and DALY rate, with EAPCs of −1.22% (95% CI, −1.41 to −1.04) and −1.27% (95% CI, −1.44 to −1.11), respectively (Table 1; Figure 5; Supplementary Tables S1, S2; Supplementary Figure S5). Over the past 32 years, air pollution, high systolic blood pressure, and tobacco use have remained the primary risk factors for ICH-related deaths and DALYs in high, high-middle, and middle SDI regions. In contrast, air pollution, high systolic blood pressure, and dietary risks have been the leading contributors to deaths and DALYs in low-middle and low SDI regions (Figures 4A–D).

### National trends

#### Incidence

**Figure 6:**
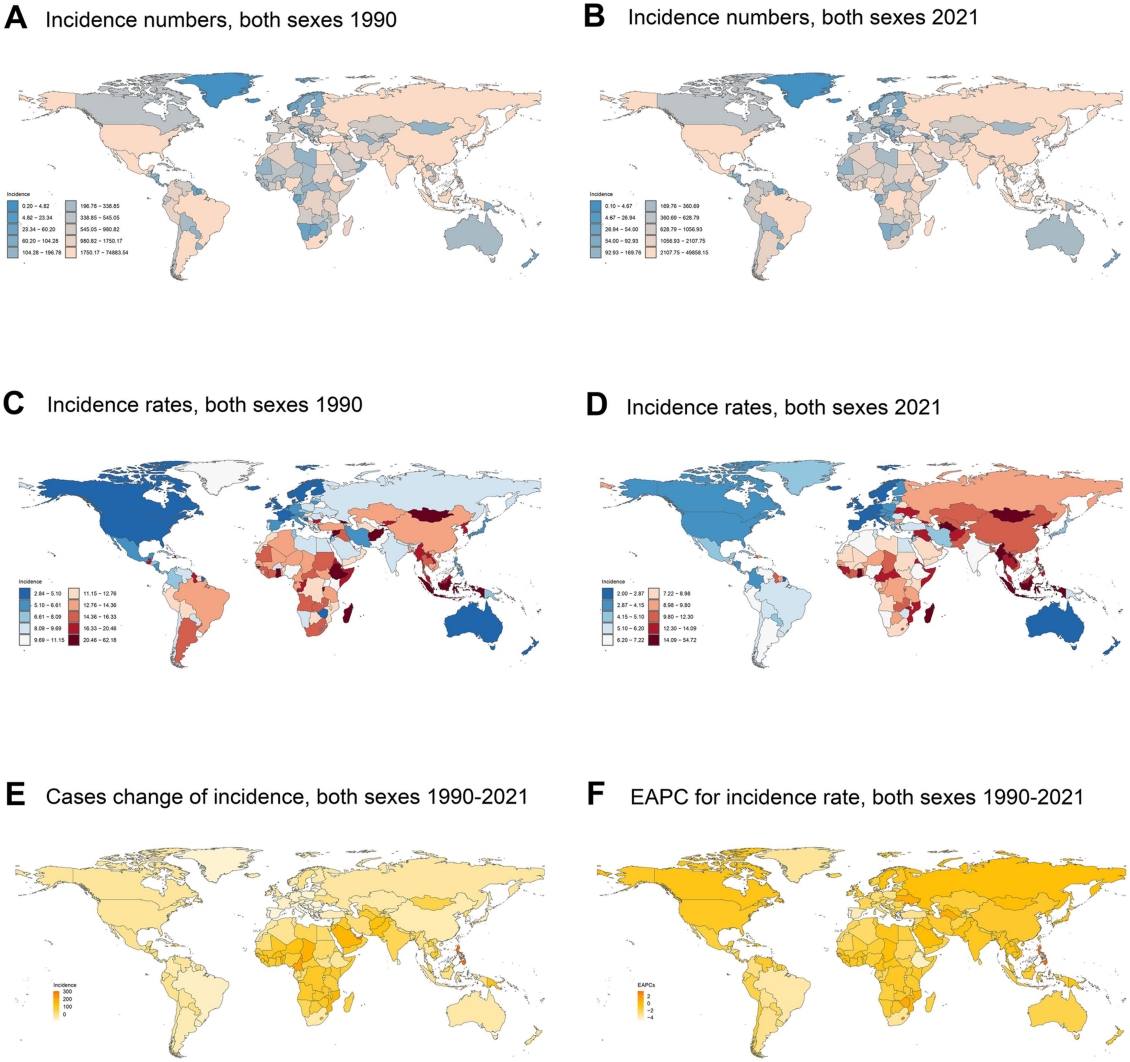
Incidence of ICH among youths and young adults across 204 countries and territories in 1990 and 2021. **(A)** Number of incidence cases in 1990. **(B)** Number of incidence cases in 2021. **(C)** Incidence rate in 1990. **(D)** Incidence rate in 2021. **(E)** Cases change of incidence between 1990 and 2021. **(F)** Estimated annual percent change (EAPC) in incidence.

In 2021, China had the highest number of ICH cases (49364.51 cases; 95% UI: 37242.00–62918.59), marking a − 33.42% (95% UI, −39.49 to −26.01%) decline from 1990 (74142.12 cases; 95% UI: 54251.50–96466.22). In contrast, South Korea had the lowest case count (1747.82 cases; 95% UI: 1467.77–2088.72), reflecting an 18.94% (95% UI, 8.06–31.80%) increase from 1990 (1469.55 cases; 95% UI: 1186.50–1764.20) (Supplementary Table S3; Figures 6A,B,E). Over the past 32 years, the Philippines exhibited the most pronounced increase in incidence rate, with an EAPC of 3.41% (95% CI, 2.86–3.97), while the Portuguese Republic experienced the steepest decline, with an EAPC of −4.78% (95% CI, −5.19 to −4.37) (Supplementary Table S3; Figures 6C,D,F). In 2021, the global incidence rate was 8.30 (95% UI, 6.46–10.19) per 100,000 population, ranking higher than 116 countries and lower than 88 countries.

#### Mortality

In 2021, China recorded the highest number of ICH-related deaths (17518.35 cases; 95% UI: 14291.91–20930.10), reflecting a − 31.66% (95% UI, −46.64 to −11.88%) decrease from 1990 (25635.06 cases; 95% UI: 21512.06–31297.82). Conversely, San Marino had the lowest number of deaths (0.03 cases; 95% UI: 0.02–0.04), marking a − 65.44% (95% UI, −80.61 to −45.34%) decline from 1990 (0.08 cases; 95% UI: 0.06–0.10) (Supplementary Table S4; Supplementary Figures S3A,B,E). Over the past 32 years, Zimbabwe exhibited the most pronounced increase in mortality rate, with an EAPC of 4.72% (95% CI, 3.52–5.94), whereas Slovenia showed the steepest decline, with an EAPC of −7.93% (95% CI, −8.82 to −7.58) (Supplementary Table S4; Supplementary Figures S3C,D,F). In 2021, the global mortality rate was 2.86 (95% UI, 2.58–3.15) per 100,000 population, ranking higher than 119 countries and lower than 85 countries.

#### DALYs

In 2021, China had the highest number of ICH-related DALYs (1102003.76; 95% UI, 918369.00–1298948.48), marking a − 33.28% (95% UI, −46.71 to −16.29) decrease from 1990 (1651705.08; 95% UI, 1400466.00–1993851.30). In contrast, San Marino recorded the lowest DALYs (2.25; 95% UI, 1.63–3.02), showing a − 57.35% (95% UI, −71.28 to −41.13) decline from 1990 (5.27; 95% UI, 4.34–6.43) (Supplementary Table S5; Supplementary Figures S4A,B,E). Over the past 32 years, Zimbabwe exhibited the most significant increase in DALYs rate, with an EAPC of 4.43% (95% CI, 3.29–5.59), whereas the Grand Duchy of Luxembourg experienced the steepest decline, with an EAPC of −6.81% (95% CI, −7.12 to −6.50) (Supplementary Table S5; Supplementary Figures S4C,D,F). In 2021, the global DALYs rate was 181.03 (95% UI, 164.20–198.70) per 100,000 population, ranking higher than 118 countries and lower than 86 countries.

## Discussion

ICH is a major cause of mortality and disability among adolescents and young adults, imposing a substantial medical burden on both families and society. A comprehensive investigation of the incidence, mortality, and DALYs of ICH is crucial for developing effective public health strategies. However, there is currently a lack of epidemiological studies focusing on ICH in individuals aged 15–39 across different global regions. Previous research has been limited to specific countries or regions. This study utilizes data from the GBD database from 1990 to 2021 to analyze the incidence, mortality, and DALYs of ICH among adolescents and young adults worldwide, stratified by region, country, sex, and age group. Furthermore, it examines specific risk factors associated with ICH. The findings provide essential evidence for developing effective prevention and control strategies.

From 1990 to 2021, the global incidence rate, mortality rate, and DALYs rate of ICH among adolescents and young adults showed an overall declining trend, with percentage changes of −26.62, −29.73%, and −30.07%, respectively. The greater reduction in mortality compared to incidence may be attributed to advancements in clinical medicine over the past 32 years, allowing more ICH patients to receive timely and effective treatment. In this study, mortality data include all deaths caused by ICH, without considering specific hemorrhage locations or volumes. This limitation may obscure the treatment effects in certain ICH patients. Notably, between 2014 and 2019, the global incidence rate showed a slight increase. A previous study attributed this increase to population aging; however, since the present study focuses on adolescents and young adults, aging alone cannot explain this trend (5). This increase may reflect improvements in diagnostic reporting, the increasing prevalence of chronic diseases such as hypertension, and lifestyle modifications. However, after 2019, the incidence rate resumed its downward trend, which may have been influenced by the COVID-19 pandemic. During this period, global healthcare resources were primarily allocated to combating the pandemic, which impacted the timely diagnosis of other diseases.

The trends in incidence and mortality of ICH vary across different SDI regions. In high SDI and high-middle SDI regions, both the number of ICH cases and deaths have shown a declining trend. These regions benefit from well-developed healthcare systems, early intervention measures, and lower birth rates. In contrast, in middle SDI, lower-middle SDI, and low SDI regions, the number of ICH cases and deaths has been increasing. This trend may be attributed to limited medical resources (11), unhealthy lifestyles (12), insufficient public health service coverage, and lower levels of socioeconomic development (13). Additionally, these regions tend to have higher birth rates, contributing to a growing at-risk population. Despite these variations, all five SDI regions have experienced a decline in both incidence and mortality rates of ICH. This overall reduction may be attributed to advancements in medical research, improvements in medical technology, and the dissemination of medical knowledge over the past 32 years. Increased public awareness of disease prevention and risk factors has likely contributed to the reduction in the overall disease burden. The decline in incidence and mortality has been most pronounced in high SDI regions, likely due to improvements in early screening programs, enhanced screening techniques, and increased clinical awareness. Furthermore, advancements in ICH treatment strategies have played a crucial role in reducing mortality rates (14).

In 2021, at the global level, China had the highest number of ICH cases and deaths, while South Korea had the lowest number of cases, and San Marino had the lowest number of deaths. These differences not only reflect variations in healthcare services and disease prevention but also highlight the impact of socioeconomic development on disease burden (6). Countries with a high incidence of ICH often face challenges such as limited medical resources, inadequate healthcare service coverage, and the widespread presence of high-risk factors such as smoking and air pollution.

In both 1990 and 2021, more than 70% of ICH incidence and mortality occurred in the young adult population aged 25–39 years. This pattern may be closely related to ICH risk factors. Hypertension is one of the most critical risk factors for ICH, and men are more likely to develop hypertension than women. Prolonged hypertension leads to vascular remodeling, resulting in lipohyalinosis and the formation of aneurysms. When these pathological vessels rupture, blood extravasates into the brain parenchyma, leading to ICH (15, 16). Animal studies have shown that increased arterial stiffness, leading to hypertension-induced vascular changes and ICH, may be attributed to the dysregulation of matrix metalloproteinases (MMPs), elastin, and collagen (17, 18). Hypertension-induced structural changes in blood vessels are a slow process, and these lesions become more prevalent with age. Regarding sex differences, the incidence, mortality, and DALY rates are higher in males than females, with the disparity widening with age. This may be related to differences in hormonal levels and lifestyle factors. Genetic differences between males and females lead to distinct hormonal levels, which further affect platelet activation, vascular reactivity, and the function of the endogenous fibrinolytic system. For instance, the proteins slit guidance ligand (SLIT3) and G protein-coupled receptor 26 (GPR26) are involved in the regulation of estrogen and serotonin (19), which influence the sex differences in ICH. For certain types of ICH, such as those associated with reversible cerebral vasoconstriction syndrome (RCVS), the impact of hormonal differences is even more pronounced (20).

In 1990, the main risk factors for death and DALYs due to ICH were air pollution, high systolic blood pressure, and tobacco use, in that order. By 2021, the primary risk factors for ICH were high systolic blood pressure, air pollution, and tobacco use. Over the past 32 years, the global economy has rapidly developed, and significant progress has been made in environmental management, leading to substantial improvements in air pollution. However, due to increased economic levels, rising work pressures, and poor lifestyle habits—such as young people tending to stay up late, binge-eating, reduced physical activity, and obesity—the incidence of hypertension among the youth has increased. Effective blood pressure control is crucial for the prevention of ICH. A previous study has found a causal relationship between smoking and ICH (21). Cigarette smoke increases the risk of stroke through various mechanisms that promote vascular damage and the formation of blood clots, including endothelial dysfunction, lipid oxidation, inflammation, and enhanced coagulation (22). Air pollution is also a significant risk factor for ICH, consistent with previous studies (23). Pollutants are deposited in the lungs through respiration, enter the bloodstream, and react with nitric oxide (NO) to generate reactive oxygen species, leading to endothelial dysfunction (24). Additionally, pollutants can trigger autonomic reflexes through pressure receptors and chemoreceptors in the lungs, resulting in increased vascular resistance and hypertension, ultimately leading to ICH. Furthermore, long-term exposure to particulate matter (PM) and gaseous pollutants may directly or indirectly damage the brain—indirectly through the aforementioned autonomic reflex arc, or directly through the diffusion or absorption of nanoparticles and gaseous pollutants across the blood–brain barrier, which locally triggers neuroinflammation and neuronal damage (25). Some studies have reported a link between ozone (O3) and cerebral hemorrhage, although the exact mechanisms remain unclear (26).

This study is the first epidemiological research using the GBD database to examine the burden of ICH among adolescents and young adults aged 15–39, providing valuable reference for healthcare professionals in developing appropriate prevention measures, management policies, and diagnostic approaches. However, this study also has some limitations. First, as a cross-sectional study based on the GBD database, the data source and accuracy are influenced by various national statistical agencies and health departments, including differences in definitions and methodological inconsistencies. These discrepancies may lead to an underestimation of ICH incidence. Second, the availability of data from underdeveloped regions is concerning, as there may be a large number of undiagnosed ICH cases, which could prevent an accurate reflection of the true disease burden. Finally, the GBD database does not provide detailed risk stratification for ICH, limiting the ability to analyze different subgroups. This limitation hampers the potential to assess disease outcomes based on severity, thereby affecting the study’s ability to offer deeper insights and limiting the assessment of ICH prognosis and treatment effectiveness.

## Conclusion

In conclusion, from 1990 to 2021, the incidence, mortality, and DALYs of ICH among adolescents and young adults have shown an overall downward trend. However, the burden increases with age. The main risk factors are hypertension, air pollution, and tobacco use. Therefore, policymakers must urgently implement more effective prevention and control measures to reduce the disease burden of ICH among adolescents and young adults, thereby alleviating the economic pressure on society.

## Data Availability

The original contributions presented in the study are included in the article/Supplementary material, further inquiries can be directed to the corresponding authors.

## Footnotes

^1^ https://vizhub.healthdata.org/gbd-results/

## References

[1] Kim J Thayabaranathan T Donnan GA Howard G Howard VJ Rothwell PM. Global stroke statistics 2019. Int J Stroke. (2020) 15:819–38. doi: 10.1177/1747493020909545, PMID: 32146867

[2] Sheth KN. Spontaneous intracerebral hemorrhage. N Engl J Med. (2022) 387:1589–96. doi: 10.1056/NEJMra2201449, PMID: 36300975

[3] Hankey GJ. Stroke. Lancet. (2017) 389:641–54. doi: 10.1016/S0140-6736(16)30962-X, PMID: 27637676

[4] Gerstl JVE Blitz SE Qu QR Yearley AG Lassarén P Lindberg R. Global, regional, and national economic consequences of stroke. Stroke. (2023) 54:2380–9. doi: 10.1161/STROKEAHA.123.043131, PMID: 37497672 PMC7614992

[5] Xu L Wang Z Wu W Li M Li Q. Global, regional, and national burden of intracerebral hemorrhage and its attributable risk factors from 1990 to 2021: results from the 2021 global burden of disease study. BMC Public Health. (2024) 24:2426. doi: 10.1186/s12889-024-19923-7, PMID: 39243077 PMC11378620

[6] Collaborators GBDRF. Global burden and strength of evidence for 88 risk factors in 204 countries and 811 subnational locations, 1990-2021: a systematic analysis for the global burden of disease study 2021. Lancet. (2024) 403:2162–203. doi: 10.1016/S0140-6736(24)00933-4, PMID: 38762324 PMC11120204

[7] Collaborators GBDCoD. Global burden of 288 causes of death and life expectancy decomposition in 204 countries and territories and 811 subnational locations, 1990-2021: a systematic analysis for the global burden of disease study 2021. Lancet. (2024) 403:2100–32. doi: 10.1016/S0140-6736(24)00367-2, PMID: 38582094 PMC11126520

[8] Kocarnik JM Compton K Dean FE Fu W Gaw BL Harvey JD. Cancer incidence, mortality, years of life lost, years lived with disability, and disability-adjusted life years for 29 cancer groups from 2010 to 2019: a systematic analysis for the global burden of disease study 2019. JAMA Oncol. (2022) 8:420–44. doi: 10.1001/jamaoncol.2021.6987 34967848 PMC8719276

[9] Kim HJ Fay MP Feuer EJ Midthune DN. Permutation tests for joinpoint regression with applications to cancer rates. Stat Med. (2000) 19:335–51. doi: 10.1002/(sici)1097-0258(20000215)19:3<335::aid-sim336>3.0.co;2-z, PMID: 10649300

[10] Cao G Liu J Liu M. Global, regional, and National Incidence and mortality of neonatal preterm birth, 1990-2019. JAMA Pediatr. (2022) 176:787–96. doi: 10.1001/jamapediatrics.2022.1622, PMID: 35639401 PMC9157382

[11] Lioutas VA Beiser AS Aparicio HJ Himali JJ Selim MH Romero JR. Assessment of incidence and risk factors of intracerebral hemorrhage among participants in the Framingham heart study between 1948 and 2016. JAMA Neurol. (2020) 77:1252–60. doi: 10.1001/jamaneurol.2020.1512, PMID: 32511690 PMC7281354

[12] Schutte AE Srinivasapura Venkateshmurthy N Mohan S Prabhakaran D. Hypertension in low- and middle-income countries. Circ Res. (2021) 128:808–26. doi: 10.1161/CIRCRESAHA.120.318729, PMID: 33793340 PMC8091106

[13] An SJ Kim TJ Yoon BW. Epidemiology, risk factors, and clinical features of intracerebral hemorrhage: An update. J Stroke. (2017) 19:3–10. doi: 10.5853/jos.2016.00864, PMID: 28178408 PMC5307940

[14] Magid-Bernstein J Girard R Polster S Srinath A Romanos S Awad IA. Cerebral hemorrhage: pathophysiology, treatment, and future directions. Circ Res. (2022) 130:1204–29. doi: 10.1161/CIRCRESAHA.121.319949, PMID: 35420918 PMC10032582

[15] Laurent S Boutouyrie P. The structural factor of hypertension: large and small artery alterations. Circ Res. (2015) 116:1007–21. doi: 10.1161/CIRCRESAHA.116.303596, PMID: 25767286

[16] Keep RF Hua Y Xi G. Intracerebral haemorrhage: mechanisms of injury and therapeutic targets. Lancet Neurol. (2012) 11:720–31. doi: 10.1016/S1474-4422(12)70104-7, PMID: 22698888 PMC3884550

[17] Wakisaka Y Chu Y Miller JD Rosenberg GA Heistad DD. Critical role for copper/zinc-superoxide dismutase in preventing spontaneous intracerebral hemorrhage during acute and chronic hypertension in mice. Stroke. (2010) 41:790–7. doi: 10.1161/STROKEAHA.109.569616, PMID: 20150548 PMC2847648

[18] Wang M Zhang J Telljohann R Jiang L Wu J Monticone RE. Chronic matrix metalloproteinase inhibition retards age-associated arterial proinflammation and increase in blood pressure. Hypertension. (2012) 60:459–66. doi: 10.1161/HYPERTENSIONAHA.112.191270, PMID: 22689745 PMC3537179

[19] Chung J Montgomery B Marini S Rosand J Anderson CD. Genome-wide interaction study with sex identifies novel loci for intracerebral hemorrhage risk. Arteriosc Thromb Vasc Biol. (2019) 39:A571–1. doi: 10.1161/atvb.39.suppl_1.571

[20] Roy-O'Reilly M McCullough LD. Sex differences in stroke: the contribution of coagulation. Exp Neurol. (2014) 259:16–27. doi: 10.1016/j.expneurol.2014.02.011, PMID: 24560819 PMC4127336

[21] Larsson SC Burgess S Michaelsson K. Smoking and stroke: a mendelian randomization study. Ann Neurol. (2019) 86:468–71. doi: 10.1002/ana.25534, PMID: 31237718 PMC6701987

[22] Rigotti NA Clair C. Managing tobacco use: the neglected cardiovascular disease risk factor. Eur Heart J. (2013) 34:3259–67. doi: 10.1093/eurheartj/eht352, PMID: 24014389

[23] Verhoeven JI Allach Y Vaartjes ICH Klijn CJM de Leeuw FE. Ambient air pollution and the risk of ischaemic and haemorrhagic stroke. Lancet Planet Health. (2021) 5:e542–52. doi: 10.1016/S2542-5196(21)00145-5, PMID: 34390672

[24] Münzel T Gori T Al-Kindi S Deanfield J Lelieveld J Daiber A. Effects of gaseous and solid constituents of air pollution on endothelial function. Eur Heart J. (2018) 39:3543–50. doi: 10.1093/eurheartj/ehy481, PMID: 30124840 PMC6174028

[25] Hahad O Lelieveld J Birklein F Lieb K Daiber A Münzel T. Ambient air pollution increases the risk of cerebrovascular and neuropsychiatric disorders through induction of inflammation and oxidative stress. Int J Mol Sci. (2020) 21:4306. doi: 10.3390/ijms21124306, PMID: 32560306 PMC7352229

[26] Wilker EH Mostofsky E Fossa A Koutrakis P Warren A Charidimou A. Ambient pollutants and spontaneous intracerebral hemorrhage in greater Boston. Stroke. (2018) 49:2764–6. doi: 10.1161/STROKEAHA.118.023128, PMID: 30580707

