# Supplementary material for: Global, regional, and national burden of intracerebral hemorrhage and attributable risk factors in youths and young adults, 1990–2021: a statistical analysis of incidence, mortality, and DALYs
Source: Front Neurol. 2025 Sep 9;16:1594166. doi: 10.3389/fneur.2025.1594166 (PMC12454068; doi:10.3389/fneur.2025.1594166)
Supplement: Supplementary file 1 [file Table_1.docx]

**Table S1** Mortality of ICH between 1990 and 2021 at the global and regional level

| **location** | **Rate per 100,000 (95% UI)** | | | | | | |
| --- | --- | --- | --- | --- | --- | --- | --- |
|  | **1990** | | **2021** | | **1990-2021** | | |
|  | **Death numbers** | **Mortality rate** | **Death numbers** | **Mortality rate** | **Cases change^b^** | **Rate change^b^** | **EAPC^a^** |
| Global | 89160.94(81821.53,96337.17) | 4.07(3.73,4.40) | 85038.37(76818.49,93855.53) | 2.86(2.58,3.15) | -4.62(-14.24,6.74) | -29.73(-36.81,-21.36) | -1.28(-1.42,-1.13) |
| **Sex** |  |  |  |  |  |  |  |
| Male | 51896.62(44359.04,58992.43) | 4.68(4.00,5.32) | 55164.97(49177.87,61621.20) | 3.65(3.26,4.08) | 6.30(-6.30,23.25) | -21.95(-31.20,-9.51) | -0.91(-1.06,-0.76) |
| Female | 37264.32(32233.62,42167.19) | 3.44(2.98,3.89) | 29873.40(26210.78,33869.65) | 2.04(1.79,2.31) | -19.83(-31.23,-8.05) | -40.72(-49.15,-32.01) | -1.87(-2.02,-1.71) |
| **Age(years)** |  |  |  |  |  |  |  |
| 15-19 | 8028.92(7238.87,8827.42) | 1.55(1.39,1.70) | 5789.56(5055.35,6477.49) | 0.93(0.81,1.04) | -27.89(-36.51,-17.86) | -39.97(-47.15,-31.63) | -1.71(-1.78,-1.65) |
| 20-24 | 10314.74(9197.40,11323.86) | 2.10(1.87,2.30) | 8498.22(7455.07,9522.44) | 1.42(1.25,1.59) | -17.61(-26.87,-7.97) | -32.11(-39.74,-24.16) | -1.37(-1.47,-1.26) |
| 25-29 | 13266.41(11993.48,14369.24) | 3.00(2.71,3.25) | 11295.75(10042.60,12703.64) | 1.92(1.71,2.16) | -14.85(-23.35,-4.90) | -35.94(-42.34,-28.45) | -1.50(-1.62,-1.38) |
| 30-34 | 21105.14(19132.69,22772.88) | 5.48(4.96,5.91) | 22261.55(20010.39,24687.38) | 3.68(3.31,4.08) | 5.48(-5.13,18.57) | -32.75(-39.51,-24.40) | -1.36(-1.49,-1.23) |
| 35-39 | 36445.72(33453.95,39352.76) | 10.35(9.50,11.17) | 37193.28(33839.34,40881.93) | 6.63(6.03,7.29) | 2.05(-8.68,14.99) | -35.91(-42.65,-27.78) | -1.49(-1.65,-1.33) |
| **SDI** |  |  |  |  |  |  |  |
| High SDI | 5673.31(5395.74,5929.80) | 1.64(1.56,1.71) | 3271.76(2971.49,3674.07) | 0.93(0.84,1.04) | -42.33(-48.30,-34.20) | -43.36(-49.22,-35.37) | -1.79(-2.01,-1.58) |
| High-middle SDI | 18400.25(16556.13,20481.92) | 4.07(3.66,4.53) | 11988.80(10553.14,13490.99) | 2.72(2.40,3.06) | -34.84(-43.77,-23.45) | -33.03(-42.21,-21.32) | -1.62(-1.85,-1.39) |
| Middle SDI | 34335.97(31215.84,38361.86) | 4.56(4.15,5.10) | 29754.24(27011.73,33050.46) | 3.21(2.91,3.56) | -13.34(-23.53,-2.15) | -29.68(-37.95,-20.60) | -1.22(-1.41,-1.04) |
| Low-middle SDI | 21610.72(18513.30,24699.12) | 4.77(4.08,5.45) | 25367.47(21682.76,29630.31) | 3.16(2.70,3.69) | 17.38(2.34,38.22) | -33.68(-42.18,-21.91) | -1.42(-1.55,-1.29) |
| Low SDI | 9050.65(7259.97,10632.85) | 4.91(3.94,5.77) | 14574.15(11824.12,17535.85) | 3.25(2.63,3.90) | 61.03(34.29,88.59) | -33.91(-44.88,-22.60) | -1.48(-1.55,-1.41) |
| **Regions** |  |  |  |  |  |  |  |
| Andean Latin America | 730.55(623.73,838.12) | 4.72(4.03,5.42) | 536.81(426.02,662.76) | 1.98(1.57,2.45) | -26.52(-43.68,-4.85) | -58.04(-67.84,-45.66) | -3.02(-3.26,-2.78) |
| Australasia | 45.20(41.54,49.49) | 0.55(0.51,0.61) | 24.42(21.56,27.51) | 0.23(0.21,0.26) | -45.99(-52.99,-36.63) | -57.94(-63.39,-50.65) | -3.25(-3.52,-2.98) |
| Caribbean | 623.64(538.58,711.91) | 4.20(3.62,4.79) | 650.67(513.81,817.52) | 3.57(2.82,4.49) | 4.34(-15.96,29.33) | -14.80(-31.37,5.61) | -0.28(-0.49,-0.08) |
| Central Asia | 1182.56(1113.15,1258.58) | 4.16(3.91,4.42) | 850.29(735.73,964.09) | 2.27(1.97,2.58) | -28.10(-38.13,-18.28) | -45.28(-52.92,-37.81) | -2.95(-3.32,-2.57) |
| Central Europe | 1560.88(1500.67,1622.36) | 3.33(3.20,3.46) | 401.02(364.60,433.43) | 1.15(1.04,1.24) | -74.31(-76.74,-71.75) | -65.63(-68.88,-62.21) | -3.79(-4.01,-3.58) |
| Central Latin America | 1536.13(1484.49,1590.48) | 2.25(2.17,2.33) | 1285.65(1122.66,1442.46) | 1.27(1.11,1.43) | -16.31(-27.43,-6.65) | -43.52(-51.03,-37.00) | -2.15(-2.53,-1.77) |
| Central Sub-Saharan Africa | 858.94(623.04,1152.63) | 4.14(3.00,5.55) | 1547.94(1062.48,2104.27) | 2.86(1.96,3.89) | 80.21(34.63,144.76) | -30.83(-48.33,-6.06) | -1.28(-1.32,-1.23) |
| East Asia | 26492.31(22343.42,32048.48) | 4.68(3.95,5.67) | 18472.87(15272.86,21898.36) | 3.86(3.19,4.57) | -30.27(-45.28,-10.46) | -17.66(-35.38,5.73) | -0.83(-1.14,-0.52) |
| Eastern Europe | 2116.51(2010.71,2203.07) | 2.47(2.34,2.57) | 1986.99(1785.25,2161.30) | 3.00(2.70,3.27) | -6.12(-15.43,4.13) | 21.68(9.61,34.97) | -0.17(-0.60,0.27) |
| Eastern Sub-Saharan Africa | 4594.24(3654.53,5529.04) | 6.48(5.16,7.80) | 6684.80(5292.38,8226.80) | 3.82(3.02,4.70) | 45.50(11.30,77.24) | -41.12(-54.96,-28.28) | -1.95(-2.07,-1.83) |
| High-income Asia Pacific | 1513.30(1343.44,1691.90) | 2.24(1.99,2.51) | 394.65(370.42,433.26) | 0.78(0.73,0.86) | -73.92(-76.98,-69.25) | -65.17(-69.25,-58.93) | -3.64(-3.86,-3.42) |
| High-income North America | 1049.06(1019.25,1077.97) | 0.93(0.90,0.95) | 790.93(736.64,838.89) | 0.64(0.60,0.68) | -24.61(-30.21,-19.63) | -30.65(-35.81,-26.06) | -1.26(-1.48,-1.03) |
| North Africa and Middle East | 7167.87(6151.04,8071.45) | 5.36(4.60,6.03) | 7012.84(5851.42,8325.50) | 2.76(2.30,3.27) | -2.16(-17.23,18.63) | -48.50(-56.44,-37.56) | -2.15(-2.25,-2.05) |
| Oceania | 205.82(146.66,283.47) | 7.75(5.52,10.67) | 384.20(266.14,520.47) | 6.82(4.72,9.24) | 86.67(32.26,166.79) | -11.99(-37.64,25.78) | -0.54(-0.64,-0.44) |
| South Asia | 13913.31(10735.71,16801.54) | 3.22(2.49,3.89) | 17527.77(14034.85,21145.38) | 2.22(1.77,2.67) | 25.98(4.51,51.66) | -31.25(-42.97,-17.24) | -1.25(-1.44,-1.07) |
| Southeast Asia | 15081.62(13630.10,16734.00) | 7.66(6.92,8.49) | 17191.06(14871.77,20248.69) | 6.20(5.36,7.30) | 13.99(-4.10,36.76) | -19.03(-31.88,-2.85) | -0.70(-0.88,-0.53) |
| Southern Latin America | 700.16(649.61,747.06) | 3.67(3.40,3.92) | 292.10(270.90,314.23) | 1.13(1.05,1.22) | -58.28(-63.03,-54.21) | -69.14(-72.66,-66.13) | -3.67(-3.99,-3.36) |
| Southern Sub-Saharan Africa | 1261.34(1145.76,1376.40) | 5.84(5.30,6.37) | 1247.92(1102.55,1425.80) | 3.67(3.24,4.19) | -1.06(-14.03,15.46) | -37.17(-45.41,-26.68) | -1.75(-2.64,-0.85) |
| Tropical Latin America | 3327.19(3217.51,3448.44) | 5.17(5.00,5.36) | 1636.68(1572.39,1711.46) | 1.85(1.78,1.94) | -50.81(-53.48,-47.88) | -64.18(-66.12,-62.04) | -3.58(-3.87,-3.29) |
| Western Europe | 2015.05(1948.27,2085.81) | 1.40(1.35,1.45) | 446.48(426.90,464.04) | 0.34(0.33,0.36) | -77.84(-79.24,-76.59) | -75.39(-76.95,-74.00) | -4.73(-4.87,-4.58) |
| Western Sub-Saharan Africa | 3185.27(2553.63,3843.86) | 4.45(3.57,5.37) | 5672.28(4307.47,6866.44) | 2.97(2.25,3.59) | 78.08(47.40,117.37) | -33.34(-44.83,-18.63) | -1.31(-1.42,-1.20) |

**Abbreviations:** EAPC, estimated annual percentage change; SDI, sociodemographic Index; UI, uncertainty interval. ^a^EAPC is expressed as 95% confidence interval. ^b^Change shows the percentage change.

**Table S2** DALYs of ICH between 1990 and 2021 at the global and regional level

| **location** | **Rate per 100,000 (95% UI)** | | | | | | |
| --- | --- | --- | --- | --- | --- | --- | --- |
|  | **1990** | | **2021** | | **1990-2021** | | |
|  | **DALYs numbers** | **DALYs rate** | **DALYs numbers** | **DALYs rate** | **Cases change^b^** | **Rate change^b^** | **EAPC^a^** |
| Global | 5673698.79(5241479.49,6127466.64) | 258.86(239.14,279.56) | 5385247.12(4884623.97,5910984.71) | 181.03(164.20,198.70) | -5.08(-13.98,5.38) | -30.07(-36.62,-22.36) | -1.30(-1.43,-1.16) |
| **Sex** |  |  |  |  |  |  |  |
| Male | 3236405.42(2779634.87,3658641.84) | 291.97(250.76,330.06) | 3404908.82(3040221.40,3772793.69) | 225.53(201.38,249.90) | 5.21(-6.88,21.13) | -22.75(-31.63,-11.06) | -0.94(-1.07,-0.81) |
| Female | 2437293.38(2136634.69,2757019.51) | 224.98(197.23,254.49) | 1980338.30(1738934.00,2228609.84) | 135.17(118.69,152.11) | -18.75(-29.93,-8.26) | -39.92(-48.19,-32.16) | -1.83(-1.98,-1.69) |
| **Age(years)** |  |  |  |  |  |  |  |
| 15-19 | 639312.98(582068.88,697002.81) | 123.08(112.06,134.19) | 466522.90(407897.24,522838.81) | 74.77(65.37,83.79) | -27.03(-34.86,-17.71) | -39.26(-45.77,-31.50) | -1.69(-1.76,-1.63) |
| 20-24 | 769416.02(689803.37,846523.76) | 156.36(140.18,172.03) | 635203.10(563859.35,705505.95) | 106.37(94.42,118.14) | -17.44(-25.68,-8.71) | -31.97(-38.75,-24.77) | -1.37(-1.47,-1.27) |
| 25-29 | 914657.03(834359.65,994530.95) | 206.65(188.50,224.69) | 787176.66(705253.34,878364.23) | 133.80(119.87,149.29) | -13.94(-21.92,-4.85) | -35.25(-41.26,-28.41) | -1.48(-1.59,-1.37) |
| 30-34 | 1310442.06(1195360.02,1417658.38) | 340.00(310.14,367.82) | 1394961.44(1260911.04,1538941.27) | 230.77(208.59,254.59) | 6.45(-3.71,18.84) | -32.13(-38.61,-24.23) | -1.35(-1.48,-1.22) |
| 35-39 | 2039870.69(1867098.37,2195736.19) | 579.11(530.06,623.36) | 2101383.02(1916233.87,2305370.73) | 374.67(341.66,411.04) | 3.02(-7.26,15.17) | -35.30(-41.76,-27.67) | -1.47(-1.62,-1.32) |
| **SDI** |  |  |  |  |  |  |  |
| High SDI | 381827.33(357041.00,406705.61) | 110.05(102.90,117.22) | 229305.32(206897.48,254787.80) | 64.91(58.57,72.13) | -39.95(-45.46,-32.88) | -41.01(-46.43,-34.08) | -1.67(-1.84,-1.50) |
| High-middle SDI | 1178897.49(1062781.56,1298041.71) | 260.51(234.85,286.84) | 760725.89(679389.58,851555.73) | 172.79(154.31,193.42) | -35.47(-43.25,-25.33) | -33.67(-41.66,-23.25) | -1.63(-1.84,-1.43) |
| Middle SDI | 2178996.89(1986866.48,2429984.31) | 289.52(263.99,322.86) | 1865296.44(1690941.91,2059387.11) | 201.11(182.31,222.04) | -14.40(-23.69,-4.15) | -30.54(-38.07,-22.22) | -1.27(-1.44,-1.11) |
| Low-middle SDI | 1353707.47(1169733.28,1534159.25) | 298.57(257.99,338.37) | 1592446.54(1369991.44,1827703.06) | 198.44(170.72,227.75) | 17.64(2.91,37.15) | -33.54(-41.86,-22.51) | -1.42(-1.55,-1.29) |
| Low SDI | 574658.09(464391.82,672044.28) | 311.79(251.96,364.63) | 932365.65(766955.27,1115159.81) | 207.63(170.79,248.33) | 62.25(36.54,88.56) | -33.41(-43.96,-22.61) | -1.47(-1.55,-1.40) |
| **Regions** |  |  |  |  |  |  |  |
| Andean Latin America | 46492.02(40034.58,53158.60) | 300.65(258.89,343.76) | 34350.92(27997.02,42030.32) | 126.85(103.39,155.21) | -26.11(-42.36,-5.47) | -57.81(-67.09,-46.02) | -2.98(-3.21,-2.76) |
| Australasia | 3308.64(3002.42,3674.07) | 40.58(36.82,45.06) | 2097.12(1816.18,2427.33) | 20.03(17.34,23.18) | -36.62(-43.62,-28.44) | -50.64(-56.10,-44.27) | -2.67(-2.89,-2.46) |
| Caribbean | 38294.60(33157.70,43558.60) | 257.62(223.06,293.03) | 39664.79(31688.87,49273.08) | 217.91(174.09,270.69) | 3.58(-15.54,27.25) | -15.42(-31.03,3.92) | -0.30(-0.50,-0.10) |
| Central Asia | 75423.33(70768.30,80343.72) | 265.07(248.71,282.37) | 55040.55(48120.62,62078.51) | 147.22(128.71,166.04) | -27.02(-36.34,-17.99) | -44.46(-51.55,-37.58) | -2.85(-3.20,-2.50) |
| Central Europe | 97823.88(93239.45,102853.66) | 208.81(199.02,219.55) | 27292.07(24963.18,29933.70) | 77.93(71.28,85.48) | -72.10(-74.45,-69.57) | -62.68(-65.83,-59.30) | -3.53(-3.72,-3.34) |
| Central Latin America | 98985.35(95028.39,102809.32) | 145.00(139.20,150.60) | 82732.41(73251.76,92841.16) | 81.78(72.41,91.77) | -16.42(-26.72,-7.09) | -43.60(-50.55,-37.30) | -2.14(-2.49,-1.78) |
| Central Sub-Saharan Africa | 55182.60(41137.82,72804.89) | 265.78(198.14,350.66) | 98903.10(70021.52,131376.14) | 182.83(129.44,242.86) | 79.23(35.84,139.21) | -31.21(-47.86,-8.19) | -1.30(-1.34,-1.25) |
| East Asia | 1708223.78(1462995.98,2047632.97) | 301.96(258.61,361.96) | 1161835.99(979207.47,1356212.86) | 242.53(204.41,283.11) | -31.99(-45.23,-15.01) | -19.68(-35.32,0.37) | -0.91(-1.18,-0.63) |
| Eastern Europe | 132392.10(125252.24,139245.63) | 154.36(146.04,162.35) | 119059.30(107796.31,128848.60) | 179.92(162.90,194.71) | -10.07(-18.56,-0.90) | 16.56(5.55,28.45) | -0.23(-0.64,0.18) |
| Eastern Sub-Saharan Africa | 291482.52(234217.52,347661.90) | 411.18(330.40,490.43) | 425456.28(339122.03,519976.03) | 242.86(193.58,296.81) | 45.96(12.58,76.27) | -40.94(-54.45,-28.67) | -1.95(-2.06,-1.83) |
| High-income Asia Pacific | 103114.61(91723.78,115153.49) | 152.77(135.90,170.61) | 30568.08(27405.73,33947.14) | 60.48(54.23,67.17) | -70.36(-73.53,-66.06) | -60.41(-64.65,-54.67) | -3.24(-3.44,-3.04) |
| High-income North America | 74567.07(69430.35,80503.30) | 65.80(61.27,71.04) | 59209.37(54069.00,65257.87) | 48.07(43.89,52.98) | -20.60(-25.42,-16.09) | -26.96(-31.40,-22.81) | -1.03(-1.15,-0.91) |
| North Africa and Middle East | 461752.77(400739.53,519160.10) | 345.03(299.44,387.93) | 454447.06(388273.46,532774.69) | 178.73(152.70,209.53) | -1.58(-15.79,17.61) | -48.20(-55.68,-38.10) | -2.15(-2.24,-2.06) |
| Oceania | 12671.22(9151.48,17322.01) | 477.01(344.51,652.09) | 23540.72(16698.85,31561.02) | 417.80(296.37,560.15) | 85.78(33.14,161.73) | -12.41(-37.23,23.39) | -0.55(-0.65,-0.45) |
| South Asia | 874356.30(687834.25,1047791.46) | 202.58(159.36,242.76) | 1106005.25(897374.78,1320095.29) | 139.84(113.46,166.90) | 26.49(5.77,50.19) | -30.97(-42.28,-18.04) | -1.26(-1.44,-1.08) |
| Southeast Asia | 940966.12(847657.50,1032982.20) | 477.64(430.27,524.34) | 1060304.71(928870.27,1234604.50) | 382.33(334.94,445.18) | 12.68(-4.25,33.45) | -19.95(-31.98,-5.20) | -0.75(-0.91,-0.59) |
| Southern Latin America | 45361.31(42349.97,48567.60) | 237.75(221.97,254.56) | 21221.92(19599.68,22936.23) | 82.27(75.98,88.91) | -53.22(-57.79,-48.60) | -65.40(-68.78,-61.99) | -3.37(-3.66,-3.08) |
| Southern Sub-Saharan Africa | 76732.40(69776.76,83455.84) | 354.99(322.81,386.10) | 76043.05(67341.81,86296.69) | 223.42(197.85,253.55) | -0.90(-13.24,14.89) | -37.06(-44.90,-27.04) | -1.74(-2.60,-0.87) |
| Tropical Latin America | 198084.85(191778.73,205196.15) | 308.00(298.20,319.06) | 99584.15(95272.32,103798.18) | 112.77(107.88,117.54) | -49.73(-52.41,-46.93) | -63.39(-65.34,-61.35) | -3.49(-3.78,-3.21) |
| Western Europe | 133005.21(127082.63,139236.63) | 92.29(88.18,96.61) | 35450.90(32123.22,39284.54) | 27.32(24.75,30.27) | -73.35(-75.21,-71.43) | -70.40(-72.47,-68.27) | -4.16(-4.31,-4.01) |
| Western Sub-Saharan Africa | 205478.12(167378.29,243462.15) | 287.08(233.85,340.15) | 372439.39(289980.12,447914.84) | 194.78(151.66,234.25) | 81.26(52.47,117.69) | -32.15(-42.93,-18.51) | -1.26(-1.37,-1.16) |

**Abbreviations:** DALYs, disability-adjusted life years; EAPC, estimated annual percentage change; SDI, sociodemographic Index; UI, uncertainty interval. ^a^EAPC is expressed as 95% confidence interval. ^b^Change shows the percentage change.

**Table S3** Incidence of ICH between 1990 and 2021 in the 204 countries and territories

| **location** | **Rate per 100,000 (95% UI)** | | | | | |
| --- | --- | --- | --- | --- | --- | --- |
|  | **1990** | | **2021** | | **1990-2021** | |
|  | **Incident cases** | **Incident rate** | **Incident cases** | **Incident rate^b^** | **Cases change^b^** | **EAPC^a^** |
| People's Republic of China | 74142.12(54251.50,96466.22) | 13.53(9.90,17.60) | 49364.51(37242.00,62918.59) | 10.70(8.07,13.64) | -33.42(-39.49,-26.01) | -1.20(-1.47,-0.92) |
| Democratic People's Republic of Korea | 1469.55(1186.50,1764.20) | 17.62(14.23,21.15) | 1747.82(1467.77,2088.72) | 17.38(14.59,20.77) | 18.94(8.06,31.80) | -0.27(-0.50,-0.04) |
| Taiwan (Province of China) | 1381.17(1074.19,1664.88) | 14.97(11.64,18.04) | 719.40(551.22,922.33) | 9.54(7.31,12.22) | -47.91(-55.13,-39.49) | -1.74(-1.86,-1.63) |
| Kingdom of Cambodia | 510.43(405.28,629.21) | 13.26(10.53,16.34) | 921.85(777.42,1092.60) | 12.73(10.73,15.08) | 80.60(61.52,105.67) | -0.41(-0.56,-0.26) |
| Republic of Indonesia | 17479.45(12826.14,22116.01) | 22.40(16.43,28.34) | 16942.45(12857.50,21263.36) | 14.88(11.29,18.67) | -3.07(-11.97,5.81) | -1.55(-1.77,-1.33) |
| Lao People's Democratic Republic | 280.39(227.28,341.84) | 18.15(14.71,22.13) | 487.55(412.63,570.21) | 15.20(12.86,17.78) | 73.88(54.16,97.67) | -0.80(-0.96,-0.63) |
| Malaysia | 1333.69(1093.20,1594.64) | 17.97(14.73,21.49) | 1741.47(1414.76,2095.98) | 12.53(10.18,15.08) | 30.58(18.45,46.80) | -1.38(-1.61,-1.15) |
| Republic of Maldives | 18.63(15.46,22.23) | 22.93(19.02,27.35) | 31.18(25.20,38.30) | 11.98(9.69,14.72) | 67.32(43.34,94.28) | -2.93(-3.39,-2.46) |
| Republic of the Union of Myanmar | 3252.07(2655.10,3926.93) | 18.94(15.47,22.87) | 3294.85(2812.46,3796.73) | 14.66(12.51,16.89) | 1.32(-9.32,12.12) | -1.00(-1.31,-0.69) |
| Republic of the Philippines | 1657.66(1129.74,2290.55) | 6.40(4.36,8.84) | 6658.13(5088.67,8342.66) | 14.09(10.77,17.66) | 301.66(228.38,384.88) | 3.41(2.86,3.97) |
| Democratic Socialist Republic of Sri Lanka | 759.64(593.35,927.97) | 10.27(8.02,12.55) | 521.95(395.19,667.27) | 6.47(4.90,8.27) | -31.29(-38.00,-23.83) | -1.91(-2.09,-1.74) |
| Kingdom of Thailand | 3771.43(3091.72,4555.26) | 14.54(11.92,17.57) | 2881.93(2447.78,3372.66) | 13.59(11.55,15.91) | -23.59(-31.86,-13.93) | -0.51(-0.76,-0.26) |
| Democratic Republic of Timor-Leste | 33.16(25.44,41.19) | 10.42(7.99,12.94) | 54.37(43.30,65.98) | 9.52(7.58,11.55) | 63.98(46.95,81.73) | -0.43(-0.50,-0.36) |
| Socialist Republic of Viet Nam | 3714.20(3016.33,4510.36) | 13.02(10.58,15.82) | 4566.11(3745.36,5464.27) | 11.89(9.76,14.23) | 22.94(10.12,35.91) | -0.49(-0.58,-0.39) |
| Republic of Fiji | 60.95(50.43,72.23) | 18.90(15.64,22.40) | 48.25(39.95,57.46) | 13.53(11.20,16.11) | -20.84(-28.27,-11.89) | -1.40(-1.51,-1.28) |
| Republic of Kiribati | 18.80(16.13,21.65) | 61.56(52.84,70.91) | 26.94(23.86,30.19) | 54.18(47.98,60.71) | 43.32(31.82,55.50) | -0.77(-0.91,-0.62) |
| Republic of the Marshall Islands | 4.82(4.05,5.67) | 28.08(23.61,33.08) | 7.89(6.77,9.12) | 33.27(28.56,38.45) | 63.82(49.95,80.57) | 0.69(0.50,0.88) |
| Federated States of Micronesia | 13.31(10.77,16.09) | 33.23(26.88,40.15) | 12.52(10.49,14.78) | 29.48(24.69,34.81) | -5.96(-13.33,2.38) | -0.37(-0.46,-0.29) |
| Independent State of Papua New Guinea | 119.44(89.98,153.73) | 7.22(5.44,9.29) | 261.45(202.91,324.17) | 6.11(4.74,7.57) | 118.90(98.65,145.50) | -0.65(-0.78,-0.51) |
| Independent State of Samoa | 12.91(10.55,15.70) | 19.27(15.76,23.45) | 17.36(14.57,20.52) | 21.60(18.13,25.54) | 34.49(21.91,49.33) | 0.24(0.01,0.46) |
| Solomon Islands | 36.27(29.52,43.49) | 28.28(23.02,33.90) | 92.93(79.67,108.31) | 33.98(29.13,39.60) | 156.22(132.76,185.32) | 0.80(0.60,1.00) |
| Kingdom of Tonga | 3.63(2.84,4.57) | 9.83(7.70,12.37) | 3.64(2.89,4.46) | 9.37(7.43,11.48) | 0.47(-10.03,12.03) | -0.16(-0.27,-0.04) |
| Republic of Vanuatu | 15.32(12.36,18.56) | 26.15(21.09,31.68) | 37.64(32.33,43.54) | 30.20(25.94,34.93) | 145.73(121.32,173.06) | 0.50(0.34,0.66) |
| Republic of Armenia | 95.53(69.41,125.37) | 6.65(4.83,8.72) | 52.76(36.76,73.51) | 4.91(3.42,6.84) | -44.77(-51.69,-37.08) | -1.31(-1.47,-1.15) |
| Republic of Azerbaijan | 401.97(321.52,479.27) | 12.65(10.12,15.08) | 327.66(263.93,398.45) | 7.74(6.23,9.41) | -18.49(-26.53,-7.75) | -2.03(-2.26,-1.81) |
| Georgia | 571.03(485.90,668.12) | 26.82(22.82,31.38) | 145.99(121.82,173.12) | 12.87(10.74,15.26) | -74.43(-77.19,-71.56) | -2.64(-3.16,-2.11) |
| Republic of Kazakhstan | 940.01(790.48,1106.59) | 13.85(11.64,16.30) | 692.54(572.41,815.47) | 9.94(8.21,11.70) | -26.33(-32.86,-19.38) | -1.53(-2.03,-1.03) |
| Kyrgyz Republic | 306.62(259.21,360.74) | 17.00(14.37,20.00) | 203.08(163.93,241.34) | 7.46(6.02,8.87) | -33.77(-40.75,-25.68) | -3.45(-3.85,-3.04) |
| Mongolia | 189.93(157.78,223.79) | 21.49(17.85,25.32) | 288.19(245.25,336.16) | 22.83(19.43,26.63) | 51.73(36.91,71.15) | -0.06(-0.39,0.27) |
| Republic of Tajikistan | 224.69(178.28,275.15) | 10.63(8.43,13.01) | 262.82(207.17,323.05) | 6.30(4.97,7.74) | 16.97(6.02,29.05) | -2.36(-2.67,-2.05) |
| Turkmenistan | 172.71(140.91,204.06) | 11.25(9.18,13.29) | 309.20(265.20,363.12) | 14.87(12.75,17.46) | 79.03(60.15,101.79) | 0.87(0.58,1.15) |
| Republic of Uzbekistan | 940.21(769.57,1124.46) | 10.95(8.97,13.10) | 1381.84(1136.32,1640.88) | 10.06(8.27,11.94) | 46.97(33.50,63.66) | -0.38(-0.48,-0.27) |
| Republic of Albania | 146.77(119.99,176.17) | 10.33(8.45,12.40) | 70.69(57.24,85.29) | 7.46(6.04,9.00) | -51.84(-55.85,-47.32) | -1.48(-1.68,-1.29) |
| Bosnia and Herzegovina | 96.85(70.76,129.55) | 5.10(3.73,6.82) | 38.07(26.65,51.35) | 3.78(2.65,5.10) | -60.69(-64.86,-56.76) | -1.29(-1.45,-1.13) |
| Republic of Bulgaria | 498.00(437.01,570.20) | 16.73(14.68,19.15) | 173.01(145.88,201.26) | 9.11(7.68,10.60) | -65.26(-68.58,-61.95) | -2.22(-2.35,-2.09) |
| Republic of Croatia | 143.39(114.80,174.05) | 7.90(6.33,9.59) | 38.82(26.82,56.18) | 3.11(2.15,4.50) | -72.93(-78.38,-66.34) | -3.60(-3.87,-3.33) |
| Czech Republic | 248.09(186.70,317.24) | 6.69(5.03,8.55) | 110.13(71.22,160.70) | 3.73(2.41,5.44) | -55.61(-64.20,-47.19) | -2.16(-2.42,-1.90) |
| Hungary | 467.02(387.46,555.46) | 12.64(10.48,15.03) | 110.66(76.24,153.09) | 4.02(2.77,5.56) | -76.31(-81.43,-70.56) | -4.18(-4.41,-3.94) |
| North Macedonia | 98.31(81.06,116.86) | 12.38(10.21,14.72) | 54.00(42.77,65.63) | 7.06(5.59,8.58) | -45.07(-50.56,-38.88) | -2.30(-2.50,-2.09) |
| Montenegro | 40.55(34.08,47.82) | 16.15(13.57,19.05) | 18.91(15.78,22.33) | 9.19(7.67,10.85) | -53.36(-57.11,-48.95) | -2.62(-2.98,-2.25) |
| Republic of Poland | 1295.68(924.78,1731.56) | 8.97(6.40,11.99) | 463.57(329.83,614.70) | 3.83(2.73,5.08) | -64.22(-68.30,-59.68) | -3.15(-3.44,-2.86) |
| Romania | 834.73(684.50,983.56) | 9.61(7.88,11.32) | 275.41(218.34,338.54) | 5.11(4.05,6.28) | -67.01(-70.11,-63.49) | -2.12(-2.24,-2.00) |
| Republic of Serbia | 363.78(299.43,437.54) | 10.13(8.34,12.18) | 123.04(90.18,160.72) | 4.15(3.04,5.42) | -66.18(-71.32,-59.80) | -3.48(-3.70,-3.25) |
| Slovak Republic | 149.65(117.58,183.94) | 7.31(5.74,8.99) | 68.48(49.40,91.79) | 4.00(2.89,5.36) | -54.24(-60.32,-48.17) | -2.13(-2.40,-1.86) |
| Republic of Slovenia | 49.75(37.32,64.41) | 6.49(4.87,8.40) | 16.14(10.34,24.85) | 2.83(1.82,4.36) | -67.57(-75.05,-59.53) | -3.20(-3.47,-2.94) |
| Republic of Belarus | 401.80(324.39,488.07) | 10.19(8.22,12.37) | 245.30(198.21,302.46) | 8.36(6.76,10.31) | -38.95(-45.42,-32.19) | -0.58(-0.79,-0.37) |
| Republic of Estonia | 49.24(39.33,60.54) | 8.67(6.93,10.66) | 14.79(9.58,22.06) | 3.74(2.42,5.58) | -69.97(-77.84,-60.04) | -3.56(-3.90,-3.22) |
| Republic of Latvia | 71.69(56.68,87.44) | 7.52(5.94,9.17) | 19.44(13.73,26.45) | 3.61(2.55,4.91) | -72.88(-77.19,-67.98) | -3.09(-3.41,-2.77) |
| Republic of Lithuania | 89.99(69.90,111.50) | 6.46(5.02,8.00) | 34.46(25.94,44.30) | 4.28(3.22,5.50) | -61.70(-65.97,-57.43) | -1.50(-1.65,-1.36) |
| Republic of Moldova | 207.32(171.03,244.28) | 11.90(9.81,14.02) | 77.07(61.53,95.60) | 6.21(4.96,7.71) | -62.83(-67.01,-58.02) | -2.39(-2.60,-2.18) |
| Russian Federation | 5168.32(3716.20,6911.66) | 8.88(6.39,11.88) | 4228.24(3175.40,5477.40) | 9.10(6.83,11.79) | -18.19(-26.19,-6.98) | -0.21(-0.54,0.13) |
| Ukraine | 1673.71(1205.23,2244.28) | 8.81(6.35,11.82) | 1708.02(1272.78,2228.72) | 12.39(9.23,16.17) | 2.05(-12.68,21.59) | 1.01(0.76,1.25) |
| Brunei Darussalam | 17.70(13.76,21.53) | 14.36(11.16,17.46) | 12.39(9.09,15.90) | 6.07(4.45,7.79) | -30.03(-40.68,-19.11) | -3.44(-3.77,-3.11) |
| Japan | 2811.92(1873.96,4023.27) | 6.27(4.18,8.98) | 1580.46(1064.93,2211.59) | 4.88(3.29,6.82) | -43.79(-48.44,-38.41) | -0.82(-1.07,-0.57) |
| Republic of Korea | 4007.67(3204.49,4836.88) | 19.04(15.22,22.98) | 1001.57(716.24,1341.74) | 6.26(4.48,8.39) | -75.01(-80.15,-69.48) | -4.63(-5.04,-4.21) |
| Republic of Singapore | 139.51(103.47,177.70) | 9.24(6.86,11.77) | 92.22(60.90,134.54) | 4.79(3.17,6.99) | -33.90(-48.55,-17.64) | -2.81(-3.16,-2.45) |
| Australia | 252.24(184.92,338.29) | 3.72(2.73,5.00) | 190.69(123.77,282.59) | 2.20(1.43,3.26) | -24.40(-39.11,-9.91) | -2.00(-2.28,-1.73) |
| New Zealand | 39.65(23.91,62.87) | 2.87(1.73,4.55) | 36.57(22.04,59.58) | 2.03(1.22,3.31) | -7.78(-20.40,2.76) | -1.44(-1.63,-1.25) |
| Principality of Andorra | 0.80(0.52,1.20) | 3.18(2.07,4.80) | 0.67(0.42,1.02) | 2.61(1.64,3.99) | -16.41(-24.46,-8.43) | -0.91(-1.04,-0.78) |
| Republic of Austria | 154.19(109.21,212.88) | 5.14(3.64,7.09) | 87.31(54.99,135.11) | 3.09(1.95,4.79) | -43.37(-54.75,-31.73) | -2.36(-2.61,-2.10) |
| Kingdom of Belgium | 205.41(155.15,263.52) | 5.52(4.17,7.08) | 82.97(53.85,125.64) | 2.37(1.54,3.59) | -59.61(-68.60,-49.61) | -3.34(-3.59,-3.09) |
| Republic of Cyprus | 14.08(9.69,19.98) | 4.58(3.15,6.50) | 16.43(10.71,23.92) | 3.28(2.14,4.77) | 16.74(1.05,30.38) | -1.64(-1.86,-1.42) |
| Kingdom of Denmark | 73.49(54.76,97.27) | 3.85(2.87,5.10) | 47.75(29.45,76.41) | 2.62(1.61,4.19) | -35.02(-49.04,-17.71) | -1.95(-2.18,-1.71) |
| Republic of Finland | 84.44(64.91,107.43) | 4.65(3.58,5.92) | 62.58(43.40,85.28) | 3.76(2.61,5.12) | -25.89(-37.29,-16.77) | -0.88(-1.03,-0.74) |
| French Republic | 947.77(734.74,1213.25) | 4.31(3.34,5.51) | 478.83(321.61,699.70) | 2.41(1.62,3.52) | -49.48(-59.09,-40.00) | -2.12(-2.29,-1.95) |
| Federal Republic of Germany | 1551.36(1158.10,2050.30) | 5.22(3.90,6.90) | 706.82(457.61,1063.13) | 2.79(1.81,4.20) | -54.44(-63.72,-44.62) | -2.73(-3.12,-2.35) |
| Hellenic Republic | 343.46(263.77,423.60) | 9.14(7.02,11.27) | 134.60(101.05,170.18) | 4.84(3.63,6.11) | -60.81(-65.59,-55.48) | -2.29(-2.48,-2.09) |
| Republic of Iceland | 4.68(3.22,6.50) | 4.50(3.10,6.26) | 3.39(2.07,5.30) | 2.84(1.73,4.43) | -27.44(-38.61,-15.18) | -2.08(-2.29,-1.88) |
| Ireland | 60.20(40.75,85.97) | 4.39(2.97,6.27) | 44.54(27.82,68.82) | 2.85(1.78,4.40) | -26.02(-38.43,-14.61) | -1.89(-2.07,-1.72) |
| State of Israel | 66.93(45.34,94.72) | 3.50(2.37,4.96) | 67.02(40.01,108.23) | 2.02(1.20,3.26) | 0.14(-19.38,17.97) | -2.24(-2.59,-1.88) |
| Republic of Italy | 1197.45(807.95,1669.92) | 5.61(3.78,7.82) | 447.54(295.44,652.53) | 2.83(1.87,4.13) | -62.63(-67.39,-58.01) | -2.61(-2.76,-2.45) |
| Grand Duchy of Luxembourg | 9.74(7.60,12.11) | 6.60(5.15,8.21) | 5.41(3.50,7.89) | 2.45(1.59,3.58) | -44.50(-56.79,-30.41) | -4.10(-4.46,-3.74) |
| Republic of Malta | 8.27(6.09,10.93) | 5.99(4.42,7.92) | 5.02(3.57,7.00) | 3.75(2.67,5.23) | -39.24(-47.33,-32.14) | -1.84(-1.98,-1.69) |
| Kingdom of the Netherlands | 254.33(170.99,362.46) | 4.22(2.84,6.01) | 144.79(90.34,224.92) | 2.74(1.71,4.26) | -43.07(-51.65,-34.59) | -1.92(-2.15,-1.68) |
| Kingdom of Norway | 53.00(32.71,82.47) | 3.31(2.04,5.16) | 39.12(22.40,63.77) | 2.20(1.26,3.59) | -26.19(-36.75,-17.29) | -1.72(-1.89,-1.54) |
| Portuguese Republic | 338.85(277.08,400.79) | 8.95(7.32,10.59) | 77.04(52.20,110.49) | 2.61(1.77,3.74) | -77.26(-82.88,-70.77) | -4.78(-5.19,-4.37) |
| Kingdom of Spain | 980.82(760.29,1213.68) | 6.61(5.13,8.18) | 303.06(195.43,459.37) | 2.44(1.57,3.70) | -69.10(-76.63,-58.69) | -3.98(-4.29,-3.67) |
| Kingdom of Sweden | 94.98(60.75,141.64) | 3.24(2.07,4.83) | 92.98(57.69,143.03) | 2.87(1.78,4.41) | -2.11(-11.33,6.19) | -0.38(-0.51,-0.25) |
| Swiss Confederation | 92.72(61.52,136.20) | 3.52(2.33,5.17) | 58.30(32.60,94.05) | 2.10(1.17,3.39) | -37.12(-52.15,-26.04) | -2.11(-2.28,-1.94) |
| United Kingdom of Great Britain and Northern Ireland | 786.21(523.89,1135.77) | 3.76(2.51,5.43) | 596.39(397.88,872.01) | 2.74(1.83,4.01) | -24.14(-30.06,-19.57) | -1.40(-1.55,-1.26) |
| Argentine Republic | 1994.78(1627.27,2371.25) | 16.33(13.32,19.41) | 1240.44(962.13,1560.36) | 7.08(5.49,8.90) | -37.82(-45.49,-28.60) | -3.18(-3.45,-2.91) |
| Republic of Chile | 509.57(371.32,670.96) | 8.90(6.48,11.71) | 438.98(306.67,610.92) | 6.20(4.33,8.63) | -13.85(-23.92,-3.32) | -1.45(-1.61,-1.28) |
| Eastern Republic of Uruguay | 119.42(92.95,146.50) | 10.51(8.18,12.90) | 64.03(44.95,87.06) | 5.35(3.76,7.28) | -46.38(-54.54,-37.71) | -2.78(-3.01,-2.55) |
| Canada | 439.24(292.27,654.38) | 3.95(2.63,5.89) | 374.36(255.89,526.64) | 3.16(2.16,4.44) | -14.77(-25.49,-2.97) | -0.69(-0.79,-0.60) |
| United States of America | 4287.42(2837.14,6129.82) | 4.20(2.78,6.00) | 3608.89(2476.97,5003.96) | 3.24(2.23,4.50) | -15.83(-22.66,-7.21) | -1.05(-1.15,-0.96) |
| Antigua and Barbuda | 2.09(1.63,2.54) | 8.09(6.31,9.84) | 1.78(1.37,2.25) | 5.19(3.99,6.53) | -14.50(-24.24,-4.96) | -1.83(-1.97,-1.68) |
| Commonwealth of the Bahamas | 11.59(9.66,13.54) | 9.82(8.18,11.47) | 11.56(9.66,13.71) | 7.48(6.24,8.86) | -0.28(-7.64,7.64) | -1.11(-1.20,-1.03) |
| Barbados | 8.38(6.59,10.22) | 7.68(6.03,9.37) | 5.04(3.86,6.37) | 5.10(3.91,6.45) | -39.86(-46.12,-32.93) | -1.71(-1.87,-1.55) |
| Belize | 4.59(3.62,5.86) | 6.27(4.95,8.00) | 10.48(8.35,12.81) | 5.55(4.42,6.79) | 128.44(108.62,151.61) | -0.81(-1.07,-0.56) |
| Republic of Cuba | 386.16(301.88,479.59) | 7.91(6.19,9.83) | 169.76(125.13,219.69) | 4.73(3.49,6.13) | -56.04(-61.89,-49.48) | -2.30(-2.56,-2.05) |
| Commonwealth of Dominica | 2.03(1.55,2.63) | 6.94(5.31,8.99) | 1.25(0.95,1.60) | 4.82(3.65,6.16) | -38.41(-44.11,-32.13) | -1.82(-2.13,-1.52) |
| Dominican Republic | 288.25(236.42,348.59) | 9.38(7.70,11.35) | 417.77(347.33,490.75) | 9.19(7.64,10.79) | 44.93(31.42,58.84) | -0.15(-0.23,-0.08) |
| Grenada | 3.80(3.15,4.49) | 11.39(9.44,13.46) | 2.34(1.89,2.87) | 5.79(4.67,7.10) | -38.33(-44.15,-31.45) | -2.48(-2.64,-2.33) |
| Republic of Guyana | 59.40(51.47,68.29) | 17.45(15.12,20.06) | 32.35(27.88,36.96) | 10.41(8.97,11.90) | -45.55(-49.03,-41.14) | -2.19(-2.41,-1.96) |
| Republic of Haiti | 433.63(363.97,514.49) | 17.81(14.95,21.14) | 658.63(552.22,778.75) | 12.00(10.06,14.19) | 51.89(39.38,64.99) | -1.43(-1.61,-1.24) |
| Jamaica | 68.65(52.65,86.78) | 6.99(5.36,8.83) | 74.26(59.40,91.34) | 6.22(4.98,7.65) | 8.17(-2.25,20.93) | -1.05(-1.29,-0.82) |
| Saint Lucia | 5.62(4.59,6.77) | 9.98(8.15,12.04) | 4.07(3.24,5.00) | 6.15(4.90,7.56) | -27.56(-33.73,-20.45) | -1.93(-2.07,-1.79) |
| Saint Vincent and the Grenadines | 4.42(3.66,5.22) | 9.61(7.97,11.36) | 2.76(2.27,3.29) | 6.68(5.49,7.96) | -37.48(-42.67,-30.98) | -1.63(-1.92,-1.33) |
| Republic of Suriname | 19.71(16.51,23.18) | 12.10(10.14,14.23) | 20.41(17.16,23.85) | 9.51(8.00,11.11) | 3.54(-5.56,13.01) | -1.07(-1.17,-0.97) |
| Republic of Trinidad and Tobago | 43.04(34.56,51.65) | 8.58(6.89,10.30) | 26.01(20.39,32.10) | 5.22(4.10,6.45) | -39.58(-46.53,-32.45) | -2.06(-2.25,-1.86) |
| Plurinational State of Bolivia | 298.20(241.79,365.17) | 12.09(9.80,14.81) | 252.12(197.43,315.12) | 5.13(4.02,6.41) | -15.45(-23.72,-6.32) | -3.24(-3.42,-3.06) |
| Republic of Ecuador | 438.31(363.61,528.30) | 10.62(8.81,12.80) | 412.50(326.87,511.58) | 5.65(4.47,7.00) | -5.89(-14.98,2.80) | -2.23(-2.34,-2.11) |
| Republic of Peru | 995.32(809.45,1210.77) | 11.22(9.12,13.65) | 924.03(736.90,1138.96) | 6.22(4.96,7.66) | -7.16(-16.21,2.94) | -2.01(-2.15,-1.87) |
| Republic of Colombia | 1002.57(777.33,1254.61) | 7.13(5.53,8.93) | 680.52(480.47,960.77) | 3.39(2.39,4.78) | -32.12(-43.69,-19.68) | -2.94(-3.18,-2.70) |
| Republic of Costa Rica | 76.59(54.71,104.21) | 5.96(4.26,8.11) | 79.81(54.86,111.43) | 4.19(2.88,5.85) | 4.20(-5.29,14.91) | -1.54(-1.71,-1.37) |
| Republic of El Salvador | 247.76(205.24,301.44) | 11.86(9.82,14.43) | 111.52(85.45,142.59) | 4.30(3.30,5.50) | -54.99(-61.89,-48.92) | -3.87(-4.23,-3.50) |
| Republic of Guatemala | 491.40(431.64,560.60) | 16.64(14.61,18.98) | 559.40(469.66,656.24) | 8.22(6.90,9.64) | 13.84(3.83,24.97) | -2.97(-3.27,-2.66) |
| Republic of Honduras | 131.11(103.01,162.01) | 7.59(5.97,9.38) | 156.65(112.07,220.12) | 3.56(2.55,5.01) | 19.48(-0.43,41.78) | -2.91(-3.12,-2.70) |
| United Mexican States | 2283.00(1595.21,3086.10) | 6.40(4.47,8.65) | 2141.99(1526.67,2866.96) | 4.16(2.96,5.57) | -6.18(-13.23,1.66) | -1.60(-1.79,-1.41) |
| Republic of Nicaragua | 89.24(66.34,116.30) | 6.05(4.49,7.88) | 102.44(69.36,146.11) | 3.60(2.44,5.13) | 14.79(-2.60,32.38) | -2.02(-2.18,-1.86) |
| Republic of Panama | 71.30(54.89,89.04) | 7.05(5.43,8.80) | 70.87(51.43,95.16) | 4.29(3.12,5.77) | -0.60(-11.20,11.30) | -1.81(-1.92,-1.70) |
| Bolivarian Republic of Venezuela | 711.02(559.85,872.42) | 8.88(6.99,10.89) | 614.39(494.24,752.29) | 6.56(5.28,8.04) | -13.59(-22.28,-4.98) | -1.32(-1.56,-1.08) |
| Federative Republic of Brazil | 8669.49(6270.08,11393.96) | 13.82(9.99,18.16) | 4427.96(3284.88,5676.67) | 5.19(3.85,6.66) | -48.92(-53.98,-43.47) | -3.65(-3.94,-3.35) |
| Republic of Paraguay | 138.82(111.68,168.26) | 8.86(7.13,10.74) | 150.31(114.27,191.47) | 4.91(3.73,6.26) | 8.28(-6.11,22.64) | -2.36(-2.55,-2.16) |
| People's Democratic Republic of Algeria | 1343.82(1089.90,1624.10) | 13.30(10.79,16.07) | 1225.91(977.40,1491.69) | 7.20(5.74,8.76) | -8.77(-18.04,1.43) | -2.50(-2.71,-2.30) |
| Kingdom of Bahrain | 25.46(20.02,31.60) | 9.93(7.81,12.33) | 41.72(31.12,53.59) | 5.93(4.42,7.62) | 63.88(43.68,83.47) | -2.24(-2.46,-2.02) |
| Arab Republic of Egypt | 1968.91(1555.40,2372.41) | 8.98(7.10,10.82) | 2401.13(1880.46,2967.02) | 5.69(4.45,7.03) | 21.95(11.16,33.77) | -1.72(-1.97,-1.47) |
| Islamic Republic of Iran | 1322.70(928.41,1799.73) | 6.09(4.27,8.29) | 1485.29(1092.77,1944.90) | 4.28(3.15,5.60) | 12.29(2.31,24.01) | -1.22(-1.34,-1.11) |
| Republic of Iraq | 1153.06(969.83,1351.69) | 16.05(13.50,18.81) | 2195.11(1853.88,2563.28) | 12.59(10.63,14.70) | 90.37(75.59,108.33) | -0.80(-0.97,-0.63) |
| Hashemite Kingdom of Jordan | 110.02(80.37,151.20) | 7.16(5.23,9.84) | 235.97(158.57,340.68) | 4.40(2.95,6.35) | 114.48(84.92,141.19) | -2.11(-2.37,-1.85) |
| State of Kuwait | 57.76(42.38,75.32) | 6.83(5.02,8.91) | 125.32(94.07,158.82) | 5.90(4.43,7.48) | 116.99(94.69,145.43) | -0.53(-0.82,-0.25) |
| Lebanese Republic | 122.26(98.11,148.32) | 10.61(8.51,12.87) | 150.37(115.70,187.23) | 6.48(4.99,8.07) | 22.99(10.31,37.16) | -1.86(-2.07,-1.65) |
| State of Libya | 154.13(121.86,188.53) | 9.17(7.25,11.22) | 218.59(177.96,261.33) | 7.28(5.93,8.71) | 41.82(29.77,56.28) | -0.78(-0.89,-0.67) |
| Kingdom of Morocco | 1165.56(943.57,1414.78) | 11.21(9.08,13.61) | 918.63(722.64,1122.61) | 6.26(4.92,7.65) | -21.19(-27.66,-14.42) | -2.17(-2.40,-1.95) |
| Palestine | 49.11(36.93,65.50) | 6.40(4.81,8.53) | 92.73(67.26,126.69) | 4.25(3.08,5.80) | 88.83(71.83,107.03) | -1.70(-1.84,-1.55) |
| Sultanate of Oman | 104.28(84.71,125.03) | 12.57(10.21,15.07) | 166.35(136.21,201.11) | 7.19(5.89,8.69) | 59.52(43.88,78.29) | -1.97(-2.07,-1.87) |
| State of Qatar | 34.88(28.18,42.20) | 14.75(11.92,17.85) | 119.31(91.95,149.92) | 7.22(5.56,9.07) | 242.11(193.43,291.49) | -2.92(-3.19,-2.64) |
| Kingdom of Saudi Arabia | 567.74(455.87,702.46) | 8.54(6.86,10.57) | 1500.48(1242.60,1806.52) | 8.10(6.71,9.75) | 164.29(138.15,192.42) | -0.05(-0.11,0.01) |
| Syrian Arab Republic | 1224.02(1029.28,1438.05) | 25.51(21.45,29.98) | 665.69(555.81,798.39) | 13.09(10.93,15.70) | -45.61(-51.25,-39.91) | -2.30(-2.43,-2.17) |
| Republic of Tunisia | 261.39(198.48,330.11) | 7.60(5.77,9.60) | 255.36(195.57,324.14) | 5.87(4.49,7.45) | -2.31(-12.00,7.71) | -1.09(-1.20,-0.97) |
| Republic of Turkey | 3111.61(2509.78,3834.79) | 13.01(10.49,16.03) | 1893.70(1375.92,2490.33) | 5.94(4.32,7.82) | -39.14(-48.23,-29.57) | -3.19(-3.44,-2.95) |
| United Arab Emirates | 101.83(80.63,123.92) | 10.65(8.43,12.96) | 393.73(305.21,510.51) | 9.80(7.60,12.71) | 286.65(229.14,356.11) | -0.61(-1.08,-0.13) |
| Republic of Yemen | 578.97(460.56,698.04) | 12.59(10.02,15.19) | 1090.75(894.21,1303.16) | 7.93(6.50,9.47) | 88.39(70.61,107.41) | -1.60(-1.67,-1.53) |
| Islamic Republic of Afghanistan | 672.93(549.28,805.48) | 21.37(17.44,25.57) | 1528.98(1273.62,1787.55) | 12.51(10.42,14.63) | 127.21(107.99,150.14) | -1.84(-2.37,-1.31) |
| People's Republic of Bangladesh | 5951.21(4877.82,7177.13) | 14.10(11.56,17.00) | 7359.69(5925.89,8885.20) | 10.70(8.61,12.91) | 23.67(13.19,37.36) | -0.99(-1.05,-0.92) |
| Kingdom of Bhutan | 18.54(13.60,24.38) | 6.88(5.05,9.05) | 16.64(12.21,21.88) | 4.80(3.52,6.31) | -10.28(-19.80,0.09) | -1.59(-1.75,-1.42) |
| Republic of India | 29376.66(21281.14,38500.51) | 8.61(6.24,11.29) | 42028.83(31367.81,53558.65) | 6.90(5.15,8.79) | 43.07(30.84,57.14) | -0.96(-1.11,-0.82) |
| Federal Democratic Republic of Nepal | 626.26(472.68,794.52) | 8.57(6.47,10.88) | 700.21(517.69,925.45) | 5.22(3.86,6.90) | 11.81(-0.91,26.19) | -1.78(-1.89,-1.67) |
| Islamic Republic of Pakistan | 4500.78(3243.02,5955.78) | 11.03(7.95,14.60) | 10285.23(7821.80,12892.52) | 10.40(7.91,13.04) | 128.52(104.62,152.55) | -0.33(-0.56,-0.10) |
| Republic of Angola | 567.69(467.46,675.22) | 14.51(11.95,17.26) | 1056.93(851.73,1288.22) | 8.69(7.00,10.59) | 86.18(68.61,106.49) | -1.74(-1.83,-1.65) |
| Central African Republic | 162.30(131.56,194.92) | 15.59(12.63,18.72) | 276.97(234.36,325.96) | 12.69(10.73,14.93) | 70.66(56.47,89.07) | -0.81(-0.88,-0.73) |
| Republic of the Congo | 160.94(132.41,194.76) | 16.99(13.98,20.56) | 199.75(160.64,243.38) | 9.02(7.25,10.98) | 24.12(13.08,36.42) | -2.29(-2.43,-2.15) |
| Democratic Republic of the Congo | 1750.17(1385.31,2149.03) | 12.22(9.67,15.00) | 3356.56(2755.46,3994.58) | 9.30(7.64,11.07) | 91.78(72.73,113.09) | -1.06(-1.12,-0.99) |
| Republic of Equatorial Guinea | 23.34(18.99,27.88) | 15.46(12.57,18.46) | 40.98(31.46,52.55) | 5.89(4.52,7.55) | 75.55(53.09,100.38) | -3.59(-3.85,-3.32) |
| Gabonese Republic | 52.66(41.80,64.07) | 13.69(10.87,16.66) | 58.96(46.96,72.57) | 7.87(6.26,9.68) | 11.97(2.63,22.51) | -2.10(-2.24,-1.95) |
| Republic of Burundi | 545.05(458.55,649.35) | 26.29(22.12,31.32) | 623.48(523.97,737.60) | 11.83(9.94,13.99) | 14.39(2.99,26.18) | -3.17(-3.47,-2.86) |
| Union of the Comoros | 29.71(24.20,35.84) | 17.20(14.01,20.75) | 29.83(23.62,36.32) | 9.63(7.62,11.72) | 0.41(-8.91,10.32) | -2.45(-2.66,-2.23) |
| Republic of Djibouti | 25.11(20.20,30.34) | 14.32(11.52,17.31) | 53.03(42.21,65.36) | 9.81(7.80,12.09) | 111.19(92.90,136.25) | -1.51(-1.64,-1.38) |
| State of Eritrea | 265.17(219.29,316.44) | 20.46(16.92,24.42) | 390.27(325.84,463.03) | 13.94(11.64,16.54) | 47.18(34.98,61.51) | -1.50(-1.61,-1.40) |
| Federal Democratic Republic of Ethiopia | 3856.55(2850.03,4918.27) | 21.11(15.60,26.92) | 3141.80(2401.40,3993.59) | 6.78(5.18,8.62) | -18.53(-26.50,-9.85) | -4.25(-4.50,-3.99) |
| Republic of Kenya | 933.88(676.01,1221.74) | 10.66(7.72,13.95) | 1754.31(1342.23,2195.51) | 8.10(6.20,10.14) | 87.85(71.51,103.86) | -1.03(-1.25,-0.82) |
| Republic of Madagascar | 1525.06(1308.04,1749.17) | 33.67(28.88,38.62) | 2339.54(1975.55,2723.13) | 19.98(16.87,23.25) | 53.41(40.68,66.72) | -1.87(-1.96,-1.78) |
| Republic of Malawi | 476.77(393.90,569.83) | 12.76(10.54,15.25) | 728.02(612.61,870.47) | 8.90(7.49,10.64) | 52.70(39.04,66.64) | -1.37(-1.54,-1.20) |
| Republic of Mauritius | 79.79(66.07,94.61) | 16.05(13.29,19.03) | 44.82(36.52,53.82) | 9.85(8.02,11.82) | -43.83(-48.97,-37.80) | -2.00(-2.20,-1.79) |
| Republic of Mozambique | 580.73(468.11,699.92) | 12.24(9.87,14.76) | 1535.38(1276.98,1813.72) | 12.77(10.62,15.08) | 164.39(137.26,191.70) | 0.47(0.29,0.65) |
| Republic of Rwanda | 833.35(698.82,990.41) | 30.41(25.50,36.14) | 526.59(431.15,641.29) | 9.29(7.60,11.31) | -36.81(-43.00,-29.34) | -4.71(-5.12,-4.31) |
| Republic of Seychelles | 5.02(4.09,5.99) | 16.08(13.10,19.18) | 4.67(3.82,5.62) | 12.18(9.96,14.63) | -6.89(-15.36,5.14) | -1.19(-1.29,-1.09) |
| Federal Republic of Somalia | 588.71(479.70,714.29) | 20.31(16.55,24.64) | 1032.13(864.37,1222.43) | 12.45(10.43,14.74) | 75.32(59.14,92.80) | -1.53(-1.65,-1.41) |
| United Republic of Tanzania | 1246.55(1033.83,1476.30) | 12.87(10.67,15.24) | 2107.75(1718.91,2556.76) | 9.03(7.37,10.96) | 69.09(54.27,85.96) | -1.31(-1.38,-1.24) |
| Republic of Uganda | 715.33(579.00,861.23) | 11.15(9.03,13.43) | 1469.46(1207.07,1771.21) | 8.54(7.02,10.30) | 105.42(87.01,125.40) | -1.32(-1.62,-1.02) |
| Republic of Zambia | 442.68(369.31,525.87) | 14.59(12.17,17.33) | 839.33(700.79,996.02) | 10.37(8.66,12.31) | 89.60(74.59,105.33) | -1.31(-1.50,-1.13) |
| Republic of Botswana | 57.71(45.46,70.16) | 11.21(8.83,13.63) | 100.96(80.89,122.28) | 9.48(7.59,11.48) | 74.94(57.29,95.06) | -1.02(-1.35,-0.70) |
| Kingdom of Lesotho | 33.37(25.85,42.03) | 6.18(4.79,7.79) | 85.61(71.52,102.14) | 10.29(8.60,12.28) | 156.56(121.95,197.34) | 2.15(1.86,2.43) |
| Republic of Namibia | 50.17(38.81,62.53) | 8.97(6.94,11.18) | 70.04(55.64,85.37) | 6.70(5.32,8.17) | 39.60(27.51,54.10) | -1.41(-1.75,-1.07) |
| Republic of South Africa | 2545.58(1886.62,3212.59) | 16.18(11.99,20.42) | 1831.81(1405.53,2279.06) | 7.55(5.80,9.40) | -28.04(-34.63,-21.51) | -3.35(-3.78,-2.91) |
| Kingdom of Eswatini | 22.73(17.82,27.82) | 7.54(5.91,9.23) | 45.88(37.76,55.56) | 9.01(7.42,10.91) | 101.86(82.99,124.51) | 0.58(0.16,1.00) |
| Republic of Zimbabwe | 181.75(135.15,234.68) | 4.58(3.41,5.92) | 360.69(291.74,436.92) | 5.69(4.60,6.89) | 98.46(71.24,126.84) | 1.02(0.60,1.43) |
| Republic of Benin | 205.69(161.99,250.74) | 12.08(9.51,14.72) | 495.13(392.70,610.94) | 9.44(7.49,11.65) | 140.71(118.32,167.57) | -0.92(-1.01,-0.83) |
| Burkina Faso | 308.78(235.44,384.93) | 9.69(7.39,12.07) | 740.63(597.11,901.18) | 8.56(6.90,10.41) | 139.86(117.04,169.69) | -0.44(-0.48,-0.40) |
| Republic of Cameroon | 527.80(426.25,633.13) | 13.88(11.21,16.65) | 1621.46(1360.34,1910.44) | 12.58(10.55,14.82) | 207.21(181.50,239.89) | -0.44(-0.63,-0.24) |
| Republic of Cabo Verde | 19.29(15.00,23.60) | 14.75(11.47,18.05) | 24.09(19.08,29.51) | 9.61(7.61,11.78) | 24.90(12.20,38.33) | -1.77(-1.96,-1.57) |
| Republic of Chad | 278.99(224.53,336.48) | 13.29(10.70,16.03) | 774.04(643.35,903.27) | 12.30(10.22,14.35) | 177.44(149.89,209.08) | -0.28(-0.47,-0.09) |
| Republic of Côte d'Ivoire | 715.55(582.50,859.11) | 15.13(12.31,18.16) | 1320.13(1093.02,1567.33) | 11.77(9.75,13.98) | 84.49(67.69,102.89) | -1.03(-1.16,-0.91) |
| Republic of the Gambia | 46.99(36.32,57.88) | 12.46(9.63,15.35) | 106.61(86.74,127.86) | 10.66(8.67,12.78) | 126.89(105.16,152.61) | -0.59(-0.76,-0.43) |
| Republic of Ghana | 1177.34(978.55,1402.05) | 20.51(17.05,24.43) | 2215.49(1842.34,2642.83) | 15.49(12.88,18.48) | 88.18(71.06,108.28) | -1.06(-1.23,-0.90) |
| Republic of Guinea | 314.60(258.14,372.87) | 15.31(12.56,18.15) | 718.99(600.08,850.09) | 13.91(11.61,16.45) | 128.54(108.27,151.38) | -0.29(-0.34,-0.25) |
| Republic of Guinea-Bissau | 79.33(66.87,94.42) | 21.39(18.03,25.46) | 134.90(115.12,158.97) | 15.99(13.64,18.84) | 70.05(55.35,85.65) | -1.05(-1.14,-0.96) |
| Republic of Liberia | 140.41(114.00,167.10) | 15.22(12.36,18.11) | 245.69(200.48,294.90) | 10.94(8.93,13.13) | 74.98(61.09,90.82) | -1.13(-1.29,-0.96) |
| Republic of Mali | 415.23(334.34,498.95) | 13.91(11.20,16.71) | 800.11(635.34,969.49) | 8.98(7.13,10.88) | 92.69(77.32,110.27) | -1.57(-1.64,-1.50) |
| Islamic Republic of Mauritania | 119.76(93.94,145.96) | 15.58(12.22,18.98) | 138.49(107.35,174.27) | 8.11(6.29,10.21) | 15.65(3.89,28.32) | -2.31(-2.40,-2.21) |
| Republic of the Niger | 395.16(319.91,475.98) | 14.20(11.49,17.10) | 865.17(700.58,1048.80) | 9.70(7.86,11.76) | 118.94(97.44,141.40) | -1.51(-1.60,-1.41) |
| Federal Republic of Nigeria | 3333.22(2388.69,4372.08) | 9.76(7.00,12.80) | 5793.47(4283.43,7492.45) | 6.44(4.76,8.33) | 73.81(60.30,88.88) | -1.54(-1.73,-1.35) |
| Democratic Republic of Sao Tome and Principe | 6.62(5.25,8.06) | 15.42(12.24,18.77) | 13.64(11.15,16.33) | 15.00(12.27,17.97) | 105.96(84.90,128.65) | -0.24(-0.47,-0.00) |
| Republic of Senegal | 358.52(278.31,438.98) | 12.99(10.09,15.91) | 628.79(504.28,757.81) | 9.75(7.82,11.75) | 75.38(61.20,91.69) | -0.96(-1.02,-0.91) |
| Republic of Sierra Leone | 215.88(170.17,263.85) | 13.51(10.65,16.52) | 460.92(376.14,545.72) | 12.36(10.08,14.63) | 113.50(90.87,138.99) | -0.34(-0.48,-0.19) |
| Togolese Republic | 196.78(159.68,235.90) | 14.35(11.65,17.21) | 408.38(333.31,488.12) | 12.14(9.91,14.51) | 107.53(91.34,127.57) | -0.63(-0.74,-0.52) |
| American Samoa | 3.63(2.99,4.35) | 17.95(14.76,21.48) | 2.65(2.18,3.14) | 15.14(12.49,17.96) | -27.13(-33.33,-20.90) | -0.82(-0.91,-0.72) |
| Bermuda | 1.60(1.13,2.14) | 6.22(4.41,8.34) | 0.75(0.49,1.10) | 4.29(2.78,6.27) | -53.02(-59.71,-45.66) | -1.62(-1.79,-1.44) |
| Cook Islands | 1.29(1.05,1.56) | 16.75(13.66,20.20) | 0.75(0.61,0.91) | 12.79(10.31,15.46) | -41.67(-47.06,-36.38) | -0.94(-1.04,-0.83) |
| Greenland | 2.71(2.14,3.30) | 10.24(8.10,12.46) | 1.01(0.77,1.27) | 4.95(3.77,6.23) | -62.75(-67.12,-57.81) | -3.39(-3.86,-2.92) |
| Guam | 7.08(5.67,8.71) | 11.16(8.95,13.74) | 6.88(5.73,8.09) | 12.41(10.33,14.60) | -2.79(-12.60,7.90) | 0.09(-0.05,0.23) |
| Principality of Monaco | 0.44(0.32,0.58) | 4.79(3.47,6.35) | 0.31(0.22,0.44) | 3.35(2.36,4.70) | -28.70(-35.49,-22.23) | -1.49(-1.60,-1.37) |
| Republic of Nauru | 1.74(1.48,2.07) | 43.13(36.66,51.29) | 1.79(1.53,2.08) | 38.47(32.96,44.76) | 2.69(-4.83,10.98) | -0.45(-0.75,-0.16) |
| Republic of Niue | 0.20(0.16,0.24) | 24.52(20.26,29.41) | 0.10(0.08,0.12) | 17.44(14.36,20.72) | -49.81(-54.12,-45.20) | -1.28(-1.43,-1.14) |
| Northern Mariana Islands | 4.33(3.47,5.32) | 18.49(14.81,22.73) | 2.05(1.69,2.49) | 12.44(10.26,15.10) | -52.62(-57.87,-46.65) | -1.58(-1.91,-1.25) |
| Republic of Palau | 1.99(1.66,2.39) | 28.57(23.76,34.27) | 1.80(1.54,2.12) | 30.51(26.08,35.92) | -9.86(-18.15,-0.01) | 0.09(-0.10,0.29) |
| Puerto Rico | 88.77(67.04,113.52) | 6.28(4.74,8.03) | 40.49(28.48,56.31) | 3.92(2.75,5.44) | -54.38(-59.86,-47.97) | -1.92(-2.08,-1.76) |
| Saint Kitts and Nevis | 3.39(2.91,3.91) | 19.59(16.86,22.62) | 1.53(1.17,1.91) | 6.71(5.17,8.40) | -54.95(-61.95,-47.68) | -4.12(-4.42,-3.81) |
| Republic of San Marino | 0.30(0.19,0.45) | 3.18(2.04,4.78) | 0.25(0.16,0.37) | 2.78(1.78,4.17) | -16.52(-24.07,-10.03) | -0.62(-0.73,-0.51) |
| Tokelau | 0.12(0.09,0.14) | 19.99(15.99,24.31) | 0.08(0.06,0.09) | 15.49(12.82,18.58) | -33.94(-39.67,-27.96) | -1.00(-1.18,-0.82) |
| Tuvalu | 1.09(0.90,1.31) | 30.27(25.07,36.30) | 1.27(1.10,1.49) | 25.66(22.04,30.08) | 16.71(5.29,28.84) | -0.73(-0.84,-0.61) |
| United States Virgin Islands | 2.69(2.05,3.44) | 6.80(5.17,8.68) | 1.00(0.70,1.38) | 4.31(3.04,5.96) | -63.00(-67.16,-57.42) | -2.10(-2.40,-1.80) |
| Republic of South Sudan | 305.99(246.02,371.75) | 13.26(10.66,16.10) | 280.42(223.73,339.89) | 7.79(6.22,9.44) | -8.36(-16.79,1.50) | -1.85(-1.95,-1.76) |
| Republic of Sudan | 1113.90(906.73,1340.00) | 14.62(11.90,17.58) | 1438.99(1170.94,1736.85) | 7.78(6.33,9.40) | 29.18(18.40,42.38) | -2.35(-2.51,-2.19) |

**Abbreviations:** EAPC, estimated annual percentage change; SDI, sociodemographic Index; UI, uncertainty interval. ^a^EAPC is expressed as 95% confidence interval. ^b^Change shows the percentage change.

**Table S4** Mortality of ICH between 1990 and 2021 in the 204 countries and territories

| **location** | **Rate per 100,000 (95% UI)** | | | | | |
| --- | --- | --- | --- | --- | --- | --- |
|  | **1990** | | **2021** | | **1990-2021** | |
|  | **Death numbers** | **Mortality rate** | **Death numbers** | **Mortality rate^b^** | **Cases change^b^** | **EAPC^a^** |
| People's Republic of China | 25635.06(21512.06,31297.82) | 4.68(3.92,5.71) | 17518.35(14291.91,20930.10) | 3.80(3.10,4.54) | -31.66(-46.64,-11.88) | -0.88(-1.20,-0.56) |
| Democratic People's Republic of Korea | 527.91(368.86,751.88) | 6.33(4.42,9.01) | 827.16(559.49,1228.31) | 8.22(5.56,12.21) | 56.69(1.62,159.16) | 0.52(0.26,0.78) |
| Taiwan (Province of China) | 329.33(307.46,353.57) | 3.57(3.33,3.83) | 127.35(113.28,139.99) | 1.69(1.50,1.86) | -61.33(-65.91,-56.51) | -1.91(-2.23,-1.59) |
| Kingdom of Cambodia | 265.64(205.46,336.56) | 6.90(5.34,8.74) | 301.95(211.76,439.46) | 4.17(2.92,6.07) | 13.67(-27.22,78.53) | -2.19(-2.43,-1.96) |
| Republic of Indonesia | 7605.11(6474.28,8661.56) | 9.74(8.30,11.10) | 8294.54(6722.35,10984.70) | 7.28(5.90,9.65) | 9.07(-15.49,49.19) | -0.82(-1.06,-0.59) |
| Lao People's Democratic Republic | 183.50(134.59,240.35) | 11.88(8.71,15.56) | 244.31(171.13,340.90) | 7.62(5.33,10.63) | 33.14(-16.69,98.83) | -1.73(-1.85,-1.61) |
| Malaysia | 377.53(322.46,438.48) | 5.09(4.34,5.91) | 460.37(386.73,542.39) | 3.31(2.78,3.90) | 21.94(-5.01,55.13) | -1.53(-1.94,-1.11) |
| Republic of Maldives | 8.11(6.59,10.84) | 9.98(8.11,13.34) | 9.25(6.96,11.58) | 3.55(2.68,4.45) | 14.04(-22.74,54.38) | -3.77(-4.28,-3.26) |
| Republic of the Union of Myanmar | 2386.65(1819.24,3171.69) | 13.90(10.60,18.48) | 1837.41(1376.44,2473.50) | 8.17(6.12,11.00) | -23.01(-45.40,13.48) | -1.91(-2.19,-1.62) |
| Republic of the Philippines | 1400.91(1264.96,1537.82) | 5.40(4.88,5.93) | 2822.84(2390.47,3358.35) | 5.97(5.06,7.11) | 101.50(68.45,142.75) | 1.11(0.73,1.49) |
| Democratic Socialist Republic of Sri Lanka | 337.65(289.65,390.87) | 4.57(3.92,5.29) | 202.47(137.67,279.35) | 2.51(1.71,3.46) | -40.04(-61.63,-13.42) | -2.30(-2.66,-1.93) |
| Kingdom of Thailand | 916.25(665.66,1181.19) | 3.53(2.57,4.55) | 1276.12(959.00,1638.48) | 6.02(4.52,7.73) | 39.28(-3.48,106.83) | 0.71(-0.10,1.53) |
| Democratic Republic of Timor-Leste | 18.79(13.77,24.40) | 5.90(4.33,7.67) | 28.27(16.76,40.74) | 4.95(2.93,7.13) | 50.47(-11.65,124.07) | -0.63(-1.23,-0.03) |
| Socialist Republic of Viet Nam | 1530.08(1133.61,2017.40) | 5.37(3.98,7.07) | 1667.73(1223.64,2367.65) | 4.34(3.19,6.17) | 9.00(-29.16,68.36) | -0.74(-1.00,-0.48) |
| Republic of Fiji | 30.01(24.83,35.92) | 9.31(7.70,11.14) | 24.35(18.26,31.98) | 6.82(5.12,8.96) | -18.88(-41.68,12.78) | -1.09(-1.29,-0.88) |
| Republic of Kiribati | 4.72(3.65,5.90) | 15.47(11.97,19.31) | 8.23(5.98,10.99) | 16.55(12.02,22.11) | 74.23(20.77,154.01) | -0.09(-0.26,0.07) |
| Republic of the Marshall Islands | 2.44(1.92,3.05) | 14.25(11.17,17.77) | 3.89(2.81,5.37) | 16.41(11.83,22.64) | 59.35(13.27,117.42) | 0.68(0.53,0.84) |
| Federated States of Micronesia | 6.84(4.88,8.82) | 17.07(12.17,22.00) | 6.15(4.48,8.13) | 14.47(10.54,19.15) | -10.14(-37.67,30.54) | -0.45(-0.51,-0.40) |
| Independent State of Papua New Guinea | 118.77(71.33,184.32) | 7.18(4.31,11.14) | 272.15(160.48,400.03) | 6.36(3.75,9.35) | 129.14(39.58,295.19) | -0.56(-0.72,-0.41) |
| Independent State of Samoa | 4.98(3.51,7.02) | 7.43(5.24,10.48) | 6.67(4.66,9.29) | 8.30(5.80,11.57) | 33.94(-12.19,104.20) | 0.40(0.24,0.56) |
| Solomon Islands | 8.08(4.32,11.61) | 6.30(3.37,9.05) | 21.47(15.09,28.75) | 7.85(5.52,10.51) | 165.81(70.03,382.59) | 0.91(0.80,1.02) |
| Kingdom of Tonga | 1.05(0.77,1.35) | 2.84(2.08,3.67) | 1.22(0.85,1.74) | 3.13(2.18,4.46) | 16.34(-22.12,80.72) | 0.54(0.41,0.67) |
| Republic of Vanuatu | 7.36(5.15,10.25) | 12.57(8.80,17.50) | 15.98(11.50,21.45) | 12.82(9.23,17.21) | 117.05(39.55,232.05) | -0.20(-0.30,-0.10) |
| Republic of Armenia | 24.31(21.06,27.51) | 1.69(1.47,1.91) | 5.13(4.34,5.93) | 0.48(0.40,0.55) | -78.90(-83.34,-73.71) | -5.26(-5.86,-4.66) |
| Republic of Azerbaijan | 125.40(90.46,166.80) | 3.95(2.85,5.25) | 69.87(44.60,100.75) | 1.65(1.05,2.38) | -44.28(-67.49,-12.00) | -4.32(-4.81,-3.82) |
| Georgia | 121.75(99.32,145.85) | 5.72(4.67,6.85) | 25.06(19.98,30.92) | 2.21(1.76,2.73) | -79.42(-84.66,-72.38) | -4.79(-6.25,-3.31) |
| Republic of Kazakhstan | 232.48(209.05,257.95) | 3.42(3.08,3.80) | 147.77(108.65,185.03) | 2.12(1.56,2.65) | -36.44(-53.87,-22.34) | -2.71(-3.57,-1.86) |
| Kyrgyz Republic | 88.84(75.57,102.11) | 4.93(4.19,5.66) | 52.20(41.87,65.00) | 1.92(1.54,2.39) | -41.24(-55.10,-24.24) | -5.14(-5.90,-4.37) |
| Mongolia | 40.42(29.46,54.47) | 4.57(3.33,6.16) | 44.87(31.72,60.23) | 3.56(2.51,4.77) | 11.02(-27.69,63.53) | -1.41(-2.01,-0.80) |
| Republic of Tajikistan | 86.33(66.60,109.36) | 4.08(3.15,5.17) | 93.44(62.64,133.34) | 2.24(1.50,3.20) | 8.24(-37.78,67.44) | -3.42(-4.02,-2.82) |
| Turkmenistan | 47.93(40.67,56.52) | 3.12(2.65,3.68) | 109.04(83.18,139.32) | 5.24(4.00,6.70) | 127.51(65.68,202.38) | 1.33(0.73,1.94) |
| Republic of Uzbekistan | 415.10(379.41,452.82) | 4.84(4.42,5.28) | 302.91(256.77,352.66) | 2.21(1.87,2.57) | -27.03(-39.67,-12.42) | -2.73(-3.41,-2.04) |
| Republic of Albania | 49.09(39.84,58.60) | 3.46(2.80,4.13) | 16.56(11.77,22.14) | 1.75(1.24,2.34) | -66.27(-76.61,-51.85) | -2.68(-3.11,-2.25) |
| Bosnia and Herzegovina | 32.16(23.25,41.77) | 1.69(1.22,2.20) | 7.04(4.71,9.73) | 0.70(0.47,0.97) | -78.12(-86.44,-67.02) | -3.34(-3.71,-2.96) |
| Republic of Bulgaria | 193.97(177.00,210.99) | 6.52(5.95,7.09) | 63.56(53.43,74.36) | 3.35(2.81,3.91) | -67.23(-73.30,-60.11) | -2.99(-3.28,-2.70) |
| Republic of Croatia | 42.17(38.10,46.61) | 2.32(2.10,2.57) | 4.59(3.79,5.56) | 0.37(0.30,0.45) | -89.11(-91.34,-86.53) | -6.41(-6.71,-6.12) |
| Czech Republic | 54.72(48.00,61.01) | 1.47(1.29,1.64) | 9.88(8.12,12.09) | 0.33(0.27,0.41) | -81.95(-85.95,-77.32) | -4.43(-4.77,-4.10) |
| Hungary | 148.14(134.34,163.44) | 4.01(3.63,4.42) | 18.31(15.05,21.93) | 0.67(0.55,0.80) | -87.64(-90.13,-84.85) | -6.44(-6.96,-5.92) |
| North Macedonia | 32.65(27.01,38.40) | 4.11(3.40,4.84) | 11.15(7.99,15.09) | 1.46(1.04,1.97) | -65.84(-75.81,-52.64) | -3.55(-3.85,-3.25) |
| Montenegro | 15.56(12.61,18.70) | 6.20(5.02,7.45) | 6.23(4.79,8.10) | 3.03(2.33,3.94) | -59.96(-70.69,-45.77) | -2.61(-3.14,-2.08) |
| Republic of Poland | 476.80(457.55,496.31) | 3.30(3.17,3.44) | 127.67(117.01,139.08) | 1.06(0.97,1.15) | -73.22(-75.71,-70.54) | -3.84(-4.27,-3.40) |
| Romania | 295.22(260.92,330.38) | 3.40(3.00,3.80) | 88.46(74.07,103.84) | 1.64(1.37,1.93) | -70.04(-75.96,-63.62) | -2.62(-2.84,-2.41) |
| Republic of Serbia | 133.32(105.77,166.68) | 3.71(2.95,4.64) | 27.24(20.24,36.02) | 0.92(0.68,1.22) | -79.57(-85.94,-70.86) | -4.81(-5.01,-4.62) |
| Slovak Republic | 52.51(42.97,63.49) | 2.57(2.10,3.10) | 13.76(10.47,18.62) | 0.80(0.61,1.09) | -73.79(-80.76,-63.28) | -3.46(-3.77,-3.16) |
| Republic of Slovenia | 9.62(8.27,11.13) | 1.26(1.08,1.45) | 0.75(0.59,0.95) | 0.13(0.10,0.17) | -92.25(-94.04,-89.61) | -7.93(-8.28,-7.58) |
| Republic of Belarus | 106.90(90.77,124.17) | 2.71(2.30,3.15) | 68.01(54.49,84.12) | 2.32(1.86,2.87) | -36.38(-51.93,-15.83) | -1.80(-2.48,-1.11) |
| Republic of Estonia | 9.00(7.56,10.53) | 1.58(1.33,1.85) | 1.62(1.34,1.93) | 0.41(0.34,0.49) | -82.02(-86.05,-76.40) | -6.25(-6.91,-5.58) |
| Republic of Latvia | 21.34(19.07,23.85) | 2.24(2.00,2.50) | 5.41(4.37,6.70) | 1.00(0.81,1.24) | -74.64(-79.59,-68.87) | -4.27(-4.87,-3.66) |
| Republic of Lithuania | 20.62(17.52,23.75) | 1.48(1.26,1.70) | 4.84(4.06,5.87) | 0.60(0.50,0.73) | -76.55(-81.29,-70.01) | -3.17(-3.75,-2.58) |
| Republic of Moldova | 70.57(62.20,79.47) | 4.05(3.57,4.56) | 24.12(20.25,28.47) | 1.94(1.63,2.30) | -65.82(-72.28,-57.33) | -3.15(-3.53,-2.76) |
| Russian Federation | 1509.35(1467.45,1543.70) | 2.59(2.52,2.65) | 1481.01(1345.12,1585.92) | 3.19(2.89,3.41) | -1.88(-11.81,6.37) | -0.07(-0.55,0.42) |
| Ukraine | 378.73(322.44,437.58) | 1.99(1.70,2.30) | 401.98(284.81,536.62) | 2.92(2.07,3.89) | 6.14(-25.43,47.83) | 0.26(-0.19,0.70) |
| Brunei Darussalam | 4.96(3.81,6.38) | 4.03(3.09,5.18) | 3.99(3.04,4.97) | 1.96(1.49,2.44) | -19.56(-44.16,18.54) | -2.93(-3.50,-2.36) |
| Japan | 577.35(560.52,597.78) | 1.29(1.25,1.33) | 246.63(238.18,256.62) | 0.76(0.73,0.79) | -57.28(-59.41,-55.02) | -1.44(-1.70,-1.18) |
| Republic of Korea | 906.99(732.67,1087.17) | 4.31(3.48,5.17) | 132.42(109.10,170.04) | 0.83(0.68,1.06) | -85.40(-88.71,-78.42) | -6.00(-6.37,-5.63) |
| Republic of Singapore | 24.00(22.10,26.02) | 1.59(1.46,1.72) | 11.61(10.30,12.99) | 0.60(0.54,0.68) | -51.62(-58.29,-44.52) | -3.75(-4.23,-3.26) |
| Australia | 36.84(33.32,41.18) | 0.54(0.49,0.61) | 20.66(18.00,23.73) | 0.24(0.21,0.27) | -43.92(-52.57,-32.31) | -3.11(-3.39,-2.83) |
| New Zealand | 8.37(7.75,9.06) | 0.61(0.56,0.66) | 3.76(3.39,4.14) | 0.21(0.19,0.23) | -55.07(-60.95,-49.25) | -3.93(-4.21,-3.64) |
| Principality of Andorra | 0.14(0.09,0.19) | 0.54(0.38,0.77) | 0.06(0.04,0.09) | 0.24(0.16,0.35) | -54.10(-72.89,-28.78) | -2.39(-2.65,-2.13) |
| Republic of Austria | 32.30(29.76,35.02) | 1.08(0.99,1.17) | 6.72(6.12,7.35) | 0.24(0.22,0.26) | -79.21(-81.60,-76.23) | -5.68(-6.09,-5.26) |
| Kingdom of Belgium | 68.51(63.26,74.42) | 1.84(1.70,2.00) | 10.34(9.31,11.42) | 0.30(0.27,0.33) | -84.91(-86.79,-82.71) | -5.83(-6.16,-5.51) |
| Republic of Cyprus | 2.67(1.96,3.46) | 0.87(0.64,1.12) | 1.55(1.16,2.00) | 0.31(0.23,0.40) | -41.97(-59.51,-17.04) | -4.41(-5.06,-3.76) |
| Kingdom of Denmark | 21.31(19.50,23.46) | 1.12(1.02,1.23) | 4.37(3.89,4.90) | 0.24(0.21,0.27) | -79.48(-82.19,-76.29) | -5.36(-5.77,-4.96) |
| Republic of Finland | 27.99(24.65,31.15) | 1.54(1.36,1.72) | 5.39(4.78,6.02) | 0.32(0.29,0.36) | -80.76(-83.36,-77.46) | -4.86(-5.26,-4.46) |
| French Republic | 268.90(247.79,292.30) | 1.22(1.13,1.33) | 54.76(49.17,60.70) | 0.28(0.25,0.31) | -79.64(-82.16,-76.96) | -5.06(-5.40,-4.71) |
| Federal Republic of Germany | 450.43(405.86,500.13) | 1.52(1.37,1.68) | 82.67(74.88,91.73) | 0.33(0.30,0.36) | -81.65(-84.18,-78.67) | -5.06(-5.48,-4.64) |
| Hellenic Republic | 102.16(93.76,111.23) | 2.72(2.49,2.96) | 27.46(24.58,30.52) | 0.99(0.88,1.10) | -73.12(-76.59,-69.29) | -3.35(-3.66,-3.04) |
| Republic of Iceland | 0.93(0.85,1.03) | 0.90(0.82,0.99) | 0.30(0.26,0.34) | 0.25(0.22,0.28) | -68.37(-73.06,-62.34) | -4.14(-4.58,-3.69) |
| Ireland | 10.23(9.44,11.11) | 0.75(0.69,0.81) | 2.23(1.96,2.52) | 0.14(0.13,0.16) | -78.24(-81.06,-74.68) | -5.17(-5.58,-4.77) |
| State of Israel | 22.48(20.76,24.44) | 1.18(1.09,1.28) | 7.50(6.54,8.51) | 0.23(0.20,0.26) | -66.65(-72.33,-60.63) | -5.50(-5.72,-5.28) |
| Republic of Italy | 309.71(300.21,320.61) | 1.45(1.41,1.50) | 76.61(71.94,81.97) | 0.49(0.46,0.52) | -75.26(-77.05,-73.21) | -3.77(-3.99,-3.55) |
| Grand Duchy of Luxembourg | 3.24(2.97,3.49) | 2.19(2.01,2.37) | 0.55(0.48,0.63) | 0.25(0.22,0.28) | -83.12(-85.42,-80.10) | -7.86(-8.22,-7.51) |
| Republic of Malta | 2.13(1.94,2.34) | 1.54(1.41,1.69) | 0.62(0.54,0.71) | 0.46(0.40,0.53) | -70.80(-75.34,-65.48) | -3.58(-3.83,-3.32) |
| Kingdom of the Netherlands | 49.63(45.39,53.97) | 0.82(0.75,0.90) | 11.36(10.26,12.61) | 0.22(0.19,0.24) | -77.12(-79.88,-73.63) | -5.04(-5.49,-4.59) |
| Kingdom of Norway | 7.62(7.28,7.99) | 0.48(0.45,0.50) | 2.01(1.90,2.14) | 0.11(0.11,0.12) | -73.60(-75.45,-71.64) | -5.04(-5.40,-4.68) |
| Portuguese Republic | 133.79(123.54,143.55) | 3.53(3.26,3.79) | 20.90(18.57,23.09) | 0.71(0.63,0.78) | -84.38(-86.42,-82.25) | -6.03(-6.49,-5.56) |
| Kingdom of Spain | 314.92(294.15,339.31) | 2.12(1.98,2.29) | 49.78(45.04,54.64) | 0.40(0.36,0.44) | -84.19(-86.09,-82.11) | -5.61(-5.82,-5.39) |
| Kingdom of Sweden | 18.65(17.03,20.43) | 0.64(0.58,0.70) | 5.12(4.43,5.83) | 0.16(0.14,0.18) | -72.53(-76.56,-67.75) | -4.16(-4.34,-3.98) |
| Swiss Confederation | 22.66(20.35,25.13) | 0.86(0.77,0.95) | 3.57(3.15,3.98) | 0.13(0.11,0.14) | -84.26(-86.74,-81.52) | -6.98(-7.32,-6.63) |
| United Kingdom of Great Britain and Northern Ireland | 142.78(140.33,145.22) | 0.68(0.67,0.69) | 72.13(69.69,74.43) | 0.33(0.32,0.34) | -49.48(-51.28,-47.69) | -2.41(-2.69,-2.12) |
| Argentine Republic | 552.69(502.60,600.45) | 4.52(4.11,4.92) | 223.59(204.84,244.24) | 1.28(1.17,1.39) | -59.54(-64.97,-54.43) | -3.94(-4.32,-3.57) |
| Republic of Chile | 113.65(104.97,122.75) | 1.98(1.83,2.14) | 56.10(50.92,61.44) | 0.79(0.72,0.87) | -50.64(-56.27,-44.42) | -2.92(-3.09,-2.75) |
| Eastern Republic of Uruguay | 33.79(30.95,36.62) | 2.98(2.72,3.22) | 12.39(11.36,13.57) | 1.04(0.95,1.13) | -63.32(-67.63,-58.54) | -3.59(-3.92,-3.25) |
| Canada | 61.25(55.46,67.92) | 0.55(0.50,0.61) | 44.82(39.79,50.52) | 0.38(0.34,0.43) | -26.82(-38.31,-14.89) | -1.63(-2.13,-1.13) |
| United States of America | 986.70(958.10,1016.02) | 0.97(0.94,0.99) | 745.81(692.67,793.48) | 0.67(0.62,0.71) | -24.41(-30.39,-19.13) | -1.25(-1.46,-1.03) |
| Antigua and Barbuda | 0.85(0.77,0.94) | 3.30(2.98,3.65) | 0.30(0.27,0.35) | 0.88(0.77,1.01) | -64.22(-69.86,-56.42) | -3.46(-4.05,-2.87) |
| Commonwealth of the Bahamas | 4.36(3.82,4.86) | 3.69(3.24,4.12) | 3.34(2.60,4.13) | 2.16(1.68,2.67) | -23.41(-42.08,0.11) | -2.61(-2.89,-2.32) |
| Barbados | 2.62(2.38,2.89) | 2.40(2.18,2.64) | 1.14(0.89,1.48) | 1.15(0.90,1.50) | -56.54(-66.84,-42.21) | -3.00(-3.36,-2.64) |
| Belize | 1.82(1.60,2.07) | 2.48(2.19,2.82) | 2.95(2.52,3.37) | 1.57(1.34,1.79) | 62.66(33.71,94.62) | -1.94(-2.47,-1.41) |
| Republic of Cuba | 112.22(102.60,121.68) | 2.30(2.10,2.49) | 40.58(34.79,47.01) | 1.13(0.97,1.31) | -63.84(-70.25,-56.95) | -2.64(-2.96,-2.32) |
| Commonwealth of Dominica | 0.48(0.40,0.58) | 1.65(1.36,2.00) | 0.40(0.30,0.51) | 1.53(1.16,1.98) | -17.63(-41.23,14.12) | -0.53(-0.77,-0.30) |
| Dominican Republic | 120.59(99.14,143.95) | 3.93(3.23,4.69) | 150.33(115.62,202.31) | 3.31(2.54,4.45) | 24.66(-7.61,77.04) | 0.11(-0.26,0.47) |
| Grenada | 1.80(1.60,2.01) | 5.40(4.79,6.02) | 0.67(0.56,0.79) | 1.65(1.38,1.95) | -62.86(-70.03,-54.35) | -3.80(-4.16,-3.43) |
| Republic of Guyana | 25.26(20.93,28.79) | 7.42(6.15,8.46) | 13.27(10.01,17.34) | 4.27(3.22,5.58) | -47.47(-62.35,-30.30) | -1.95(-2.28,-1.61) |
| Republic of Haiti | 260.28(186.43,347.06) | 10.69(7.66,14.26) | 365.50(248.55,516.87) | 6.66(4.53,9.42) | 40.42(-4.13,97.73) | -1.24(-1.46,-1.01) |
| Jamaica | 24.26(20.25,29.32) | 2.47(2.06,2.98) | 21.12(15.68,28.42) | 1.77(1.31,2.38) | -12.95(-42.15,28.16) | -2.17(-3.02,-1.30) |
| Saint Lucia | 2.02(1.86,2.20) | 3.59(3.31,3.92) | 1.18(0.97,1.41) | 1.79(1.47,2.14) | -41.61(-52.59,-27.81) | -2.77(-3.12,-2.43) |
| Saint Vincent and the Grenadines | 1.97(1.78,2.19) | 4.28(3.87,4.76) | 1.04(0.89,1.21) | 2.52(2.15,2.93) | -47.09(-56.59,-36.46) | -2.56(-2.91,-2.22) |
| Republic of Suriname | 8.92(6.17,10.70) | 5.48(3.79,6.57) | 8.63(6.73,11.06) | 4.02(3.14,5.15) | -3.21(-27.91,38.48) | -1.47(-1.83,-1.10) |
| Republic of Trinidad and Tobago | 14.66(13.45,16.11) | 2.92(2.68,3.21) | 11.63(8.80,15.14) | 2.34(1.77,3.04) | -20.68(-41.67,5.37) | -1.46(-1.94,-0.97) |
| Plurinational State of Bolivia | 160.18(117.76,220.51) | 6.50(4.78,8.94) | 108.76(71.75,153.20) | 2.21(1.46,3.12) | -32.10(-59.45,1.71) | -3.86(-4.18,-3.54) |
| Republic of Ecuador | 190.56(175.42,204.99) | 4.62(4.25,4.97) | 97.53(76.76,119.96) | 1.34(1.05,1.64) | -48.82(-59.53,-36.18) | -4.34(-4.77,-3.91) |
| Republic of Peru | 379.80(306.93,462.82) | 4.28(3.46,5.22) | 330.52(248.21,432.46) | 2.22(1.67,2.91) | -12.97(-38.71,20.37) | -2.14(-2.51,-1.77) |
| Republic of Colombia | 323.37(301.13,347.79) | 2.30(2.14,2.47) | 136.32(112.64,162.60) | 0.68(0.56,0.81) | -57.84(-65.51,-49.15) | -4.10(-4.62,-3.58) |
| Republic of Costa Rica | 13.08(11.79,14.40) | 1.02(0.92,1.12) | 10.52(9.13,11.95) | 0.55(0.48,0.63) | -19.59(-31.67,-4.59) | -2.76(-3.27,-2.25) |
| Republic of El Salvador | 76.96(64.66,89.88) | 3.68(3.09,4.30) | 37.22(28.51,47.32) | 1.44(1.10,1.83) | -51.63(-64.74,-34.81) | -3.01(-3.60,-2.42) |
| Republic of Guatemala | 144.00(133.67,155.19) | 4.87(4.52,5.25) | 156.73(133.19,181.15) | 2.30(1.96,2.66) | 8.84(-9.33,28.25) | -3.53(-4.10,-2.96) |
| Republic of Honduras | 102.80(80.84,128.54) | 5.95(4.68,7.45) | 96.21(49.36,151.56) | 2.19(1.12,3.45) | -6.41(-54.08,48.79) | -3.89(-4.25,-3.53) |
| United Mexican States | 600.79(584.97,617.82) | 1.68(1.64,1.73) | 590.57(530.42,649.04) | 1.15(1.03,1.26) | -1.70(-12.37,8.69) | -1.32(-1.78,-0.87) |
| Republic of Nicaragua | 32.48(27.77,37.53) | 2.20(1.88,2.54) | 30.92(24.78,38.62) | 1.09(0.87,1.36) | -4.80(-26.76,26.69) | -2.33(-2.59,-2.06) |
| Republic of Panama | 19.77(18.02,21.62) | 1.95(1.78,2.14) | 18.00(14.67,21.94) | 1.09(0.89,1.33) | -8.95(-27.40,12.78) | -2.17(-2.48,-1.86) |
| Bolivarian Republic of Venezuela | 222.87(203.28,243.83) | 2.78(2.54,3.04) | 209.15(151.16,274.40) | 2.23(1.61,2.93) | -6.16(-32.75,24.11) | -1.38(-1.97,-0.78) |
| Federative Republic of Brazil | 3278.01(3167.29,3395.79) | 5.22(5.05,5.41) | 1591.51(1523.96,1663.55) | 1.87(1.79,1.95) | -51.45(-54.15,-48.51) | -3.59(-3.89,-3.30) |
| Republic of Paraguay | 49.18(39.90,58.19) | 3.14(2.55,3.71) | 45.18(33.75,58.87) | 1.48(1.10,1.92) | -8.14(-34.46,29.42) | -2.53(-2.71,-2.35) |
| People's Democratic Republic of Algeria | 453.33(338.79,583.67) | 4.49(3.35,5.78) | 383.47(265.74,538.01) | 2.25(1.56,3.16) | -15.41(-37.80,15.17) | -2.53(-2.77,-2.30) |
| Kingdom of Bahrain | 8.02(6.77,9.35) | 3.13(2.64,3.65) | 13.24(10.72,15.96) | 1.88(1.52,2.27) | 65.02(25.92,118.67) | -2.18(-2.60,-1.76) |
| Arab Republic of Egypt | 1546.05(1161.66,1968.04) | 7.05(5.30,8.98) | 1182.31(837.09,1571.53) | 2.80(1.98,3.72) | -23.53(-43.33,1.36) | -2.74(-3.12,-2.35) |
| Islamic Republic of Iran | 422.70(356.48,471.97) | 1.95(1.64,2.17) | 468.90(428.09,512.86) | 1.35(1.23,1.48) | 10.93(-4.87,34.59) | -0.88(-1.11,-0.64) |
| Republic of Iraq | 530.37(424.17,659.73) | 7.38(5.90,9.18) | 644.54(484.97,875.31) | 3.70(2.78,5.02) | 21.53(-11.10,77.20) | -2.31(-2.63,-2.00) |
| Hashemite Kingdom of Jordan | 64.25(52.70,77.62) | 4.18(3.43,5.05) | 74.57(60.60,91.28) | 1.39(1.13,1.70) | 16.07(-12.70,54.62) | -4.27(-4.64,-3.89) |
| State of Kuwait | 14.80(13.19,16.35) | 1.75(1.56,1.94) | 21.75(17.85,26.32) | 1.02(0.84,1.24) | 46.91(19.74,82.05) | -2.41(-3.60,-1.20) |
| Lebanese Republic | 64.27(46.83,85.21) | 5.57(4.06,7.39) | 38.87(31.53,48.47) | 1.68(1.36,2.09) | -39.52(-59.88,-9.49) | -3.49(-4.08,-2.89) |
| State of Libya | 66.01(49.00,86.55) | 3.93(2.92,5.15) | 105.50(71.24,150.67) | 3.52(2.37,5.02) | 59.84(13.84,123.17) | -0.02(-0.38,0.34) |
| Kingdom of Morocco | 563.01(373.04,779.34) | 5.42(3.59,7.50) | 351.56(211.82,553.73) | 2.39(1.44,3.77) | -37.56(-58.49,5.51) | -2.84(-3.04,-2.64) |
| Palestine | 33.68(25.23,44.92) | 4.39(3.29,5.85) | 45.83(36.99,54.45) | 2.10(1.69,2.49) | 36.08(-1.73,93.42) | -2.37(-2.70,-2.05) |
| Sultanate of Oman | 31.79(22.89,42.48) | 3.83(2.76,5.12) | 38.68(29.09,50.10) | 1.67(1.26,2.16) | 21.67(-17.96,80.68) | -1.98(-2.28,-1.68) |
| State of Qatar | 7.92(6.20,9.98) | 3.35(2.62,4.22) | 17.83(13.00,24.64) | 1.08(0.79,1.49) | 125.12(54.69,224.28) | -4.18(-4.63,-3.72) |
| Kingdom of Saudi Arabia | 343.11(252.60,457.40) | 5.16(3.80,6.88) | 778.59(549.49,1100.54) | 4.20(2.97,5.94) | 126.92(41.70,269.23) | -0.35(-0.57,-0.12) |
| Syrian Arab Republic | 548.18(431.66,678.83) | 11.43(9.00,14.15) | 232.97(178.71,311.87) | 4.58(3.51,6.13) | -57.50(-70.86,-36.60) | -2.96(-3.34,-2.58) |
| Republic of Tunisia | 95.67(70.09,125.79) | 2.78(2.04,3.66) | 78.92(49.66,112.58) | 1.81(1.14,2.59) | -17.51(-42.18,14.98) | -1.61(-1.70,-1.53) |
| Republic of Turkey | 1101.22(867.42,1374.58) | 4.60(3.63,5.75) | 443.71(351.48,545.10) | 1.39(1.10,1.71) | -59.71(-70.67,-46.46) | -4.10(-4.45,-3.75) |
| United Arab Emirates | 26.41(19.34,36.96) | 2.76(2.02,3.87) | 55.15(38.47,75.04) | 1.37(0.96,1.87) | 108.84(39.74,209.68) | -2.22(-2.45,-1.99) |
| Republic of Yemen | 268.51(138.23,397.57) | 5.84(3.01,8.65) | 491.91(302.71,729.33) | 3.57(2.20,5.30) | 83.20(20.25,189.85) | -1.67(-1.85,-1.49) |
| Islamic Republic of Afghanistan | 303.41(197.53,408.28) | 9.63(6.27,12.96) | 797.31(547.15,1126.45) | 6.52(4.48,9.22) | 162.78(87.99,278.24) | -1.19(-1.70,-0.68) |
| People's Republic of Bangladesh | 3255.62(2258.65,4165.69) | 7.71(5.35,9.87) | 3420.04(2124.87,4762.34) | 4.97(3.09,6.92) | 5.05(-24.94,44.36) | -1.42(-1.70,-1.15) |
| Kingdom of Bhutan | 5.75(3.30,8.41) | 2.14(1.22,3.12) | 4.65(2.61,7.11) | 1.34(0.75,2.05) | -19.23(-52.64,41.49) | -2.05(-2.22,-1.88) |
| Republic of India | 9390.88(7274.66,11625.60) | 2.75(2.13,3.41) | 10800.82(8772.69,12947.15) | 1.77(1.44,2.12) | 15.01(-6.15,38.58) | -1.45(-1.73,-1.17) |
| Federal Democratic Republic of Nepal | 225.71(148.25,317.25) | 3.09(2.03,4.34) | 222.26(137.12,327.94) | 1.66(1.02,2.45) | -1.53(-37.77,51.44) | -2.02(-2.15,-1.90) |
| Islamic Republic of Pakistan | 1035.35(679.03,1346.58) | 2.54(1.66,3.30) | 3080.00(2168.23,4126.87) | 3.11(2.19,4.17) | 197.48(115.28,345.17) | 0.28(-0.08,0.64) |
| Republic of Angola | 190.32(136.22,254.26) | 4.86(3.48,6.50) | 352.87(243.29,472.92) | 2.90(2.00,3.89) | 85.41(20.48,177.29) | -1.60(-1.78,-1.43) |
| Central African Republic | 62.45(42.40,85.69) | 6.00(4.07,8.23) | 110.53(66.26,165.41) | 5.06(3.03,7.58) | 76.99(22.13,152.13) | -0.70(-0.82,-0.59) |
| Republic of the Congo | 52.66(35.71,74.37) | 5.56(3.77,7.85) | 81.31(52.39,124.68) | 3.67(2.36,5.63) | 54.39(-2.42,144.19) | -1.60(-1.87,-1.33) |
| Democratic Republic of the Congo | 530.16(357.48,745.55) | 3.70(2.50,5.20) | 972.50(626.84,1419.30) | 2.69(1.74,3.93) | 83.43(29.24,168.86) | -1.11(-1.20,-1.03) |
| Republic of Equatorial Guinea | 8.73(5.97,12.52) | 5.78(3.95,8.29) | 14.37(7.95,23.71) | 2.07(1.14,3.41) | 64.68(-4.24,174.99) | -3.96(-4.41,-3.50) |
| Gabonese Republic | 14.62(10.50,19.35) | 3.80(2.73,5.03) | 16.36(9.61,25.14) | 2.18(1.28,3.35) | 11.95(-27.87,82.75) | -2.03(-2.24,-1.83) |
| Republic of Burundi | 227.14(164.99,306.72) | 10.96(7.96,14.80) | 252.15(178.82,331.75) | 4.78(3.39,6.29) | 11.01(-24.95,64.86) | -3.25(-3.73,-2.77) |
| Union of the Comoros | 11.67(4.63,16.37) | 6.75(2.68,9.48) | 10.98(7.76,14.77) | 3.54(2.51,4.77) | -5.91(-41.01,119.61) | -2.86(-3.64,-2.08) |
| Republic of Djibouti | 8.30(5.71,11.75) | 4.73(3.26,6.70) | 17.77(11.04,27.21) | 3.29(2.04,5.03) | 114.16(33.61,242.12) | -1.37(-1.60,-1.13) |
| State of Eritrea | 121.10(86.01,162.00) | 9.34(6.64,12.50) | 159.45(103.48,235.70) | 5.69(3.70,8.42) | 31.68(-15.23,96.24) | -1.69(-1.83,-1.55) |
| Federal Democratic Republic of Ethiopia | 1267.84(936.25,1811.08) | 6.94(5.12,9.91) | 1174.14(862.48,1479.14) | 2.53(1.86,3.19) | -7.39(-44.21,30.97) | -3.80(-4.05,-3.55) |
| Republic of Kenya | 244.67(188.76,297.58) | 2.79(2.16,3.40) | 527.14(375.42,712.88) | 2.43(1.73,3.29) | 115.45(68.17,176.88) | -0.11(-0.45,0.22) |
| Republic of Madagascar | 660.72(541.73,792.02) | 14.59(11.96,17.49) | 1189.31(827.77,1598.36) | 10.16(7.07,13.65) | 80.00(20.45,151.64) | -1.16(-1.27,-1.06) |
| Republic of Malawi | 192.70(143.31,248.26) | 5.16(3.84,6.64) | 375.09(257.17,495.84) | 4.58(3.14,6.06) | 94.64(38.95,172.65) | -0.56(-0.81,-0.31) |
| Republic of Mauritius | 27.81(25.27,30.44) | 5.59(5.08,6.12) | 20.60(18.49,22.65) | 4.53(4.06,4.98) | -25.91(-35.58,-14.73) | 0.45(0.02,0.87) |
| Republic of Mozambique | 214.75(153.74,279.18) | 4.53(3.24,5.89) | 689.26(444.47,970.99) | 5.73(3.70,8.08) | 220.96(110.85,363.59) | 1.58(1.27,1.90) |
| Republic of Rwanda | 373.30(271.96,494.19) | 13.62(9.92,18.03) | 192.83(124.23,276.46) | 3.40(2.19,4.87) | -48.34(-68.08,-19.97) | -5.84(-6.53,-5.15) |
| Republic of Seychelles | 1.77(1.49,2.10) | 5.66(4.77,6.72) | 1.23(1.01,1.48) | 3.21(2.64,3.85) | -30.28(-45.67,-11.50) | -1.56(-1.85,-1.26) |
| Federal Republic of Somalia | 238.22(153.65,344.08) | 8.22(5.30,11.87) | 409.79(223.63,596.90) | 4.94(2.70,7.20) | 72.02(9.49,164.73) | -1.51(-1.84,-1.17) |
| United Republic of Tanzania | 453.48(342.82,580.01) | 4.68(3.54,5.99) | 668.99(453.71,925.26) | 2.87(1.94,3.97) | 47.52(-1.70,119.35) | -1.92(-2.09,-1.74) |
| Republic of Uganda | 274.92(182.12,387.20) | 4.29(2.84,6.04) | 515.45(341.91,724.48) | 3.00(1.99,4.21) | 87.49(18.17,189.82) | -2.31(-2.77,-1.84) |
| Republic of Zambia | 186.20(142.54,241.45) | 6.14(4.70,7.96) | 354.32(224.96,500.78) | 4.38(2.78,6.19) | 90.29(15.34,180.81) | -1.46(-1.72,-1.21) |
| Republic of Botswana | 22.45(12.44,33.48) | 4.36(2.42,6.50) | 23.00(11.89,35.17) | 2.16(1.12,3.30) | 2.45(-36.41,56.19) | -2.46(-2.77,-2.15) |
| Kingdom of Lesotho | 12.90(7.06,18.60) | 2.39(1.31,3.45) | 43.36(30.40,60.76) | 5.21(3.65,7.30) | 236.22(109.08,572.51) | 3.85(3.20,4.50) |
| Republic of Namibia | 18.45(11.32,24.79) | 3.30(2.02,4.43) | 27.16(15.10,42.91) | 2.60(1.44,4.11) | 47.18(-7.29,135.32) | -1.18(-1.70,-0.66) |
| Republic of South Africa | 1140.31(1028.18,1264.20) | 7.25(6.53,8.03) | 862.53(764.16,976.45) | 3.56(3.15,4.03) | -24.36(-34.27,-12.82) | -2.99(-4.01,-1.97) |
| Kingdom of Eswatini | 10.40(6.73,14.02) | 3.45(2.23,4.65) | 22.50(13.55,32.97) | 4.42(2.66,6.48) | 116.48(20.51,276.44) | 1.33(0.49,2.17) |
| Republic of Zimbabwe | 56.84(43.45,72.22) | 1.43(1.10,1.82) | 269.37(184.98,379.09) | 4.25(2.92,5.98) | 373.88(212.58,627.16) | 4.72(3.52,5.94) |
| Republic of Benin | 62.14(47.77,81.90) | 3.65(2.81,4.81) | 145.02(103.80,194.03) | 2.77(1.98,3.70) | 133.39(62.12,228.96) | -0.98(-1.18,-0.77) |
| Burkina Faso | 102.93(72.83,139.01) | 3.23(2.28,4.36) | 235.62(164.16,313.75) | 2.72(1.90,3.63) | 128.90(58.60,218.31) | -0.43(-0.63,-0.23) |
| Republic of Cameroon | 205.44(147.59,267.26) | 5.40(3.88,7.03) | 582.23(365.22,822.99) | 4.52(2.83,6.38) | 183.41(91.96,320.72) | -0.56(-0.94,-0.18) |
| Republic of Cabo Verde | 6.85(5.35,8.28) | 5.24(4.09,6.34) | 5.65(3.73,7.64) | 2.26(1.49,3.05) | -17.44(-41.92,13.00) | -2.90(-3.19,-2.60) |
| Republic of Chad | 96.30(71.31,129.61) | 4.59(3.40,6.17) | 268.51(183.91,378.10) | 4.27(2.92,6.01) | 178.81(90.27,303.05) | -0.19(-0.44,0.05) |
| Republic of Côte d'Ivoire | 243.09(177.15,327.41) | 5.14(3.75,6.92) | 482.64(317.96,668.30) | 4.30(2.84,5.96) | 98.55(31.85,193.42) | -0.41(-0.60,-0.22) |
| Republic of the Gambia | 19.45(13.82,26.36) | 5.16(3.66,6.99) | 47.06(33.67,64.85) | 4.71(3.37,6.48) | 141.92(58.90,278.56) | -0.61(-0.90,-0.31) |
| Republic of Ghana | 520.96(383.85,683.09) | 9.08(6.69,11.90) | 786.93(563.05,1042.80) | 5.50(3.94,7.29) | 51.05(3.75,125.28) | -1.47(-1.65,-1.30) |
| Republic of Guinea | 113.57(85.95,152.56) | 5.53(4.18,7.43) | 249.57(167.09,344.59) | 4.83(3.23,6.67) | 119.75(48.08,220.10) | -0.17(-0.27,-0.06) |
| Republic of Guinea-Bissau | 41.74(29.57,56.44) | 11.26(7.97,15.22) | 67.31(49.03,91.44) | 7.98(5.81,10.84) | 61.27(11.95,139.57) | -1.06(-1.10,-1.02) |
| Republic of Liberia | 49.50(36.94,66.34) | 5.37(4.00,7.19) | 106.26(69.84,155.29) | 4.73(3.11,6.92) | 114.67(39.66,225.77) | -0.32(-0.59,-0.04) |
| Republic of Mali | 201.07(146.87,268.14) | 6.73(4.92,8.98) | 379.72(267.56,509.44) | 4.26(3.00,5.72) | 88.85(36.33,166.91) | -1.40(-1.53,-1.26) |
| Islamic Republic of Mauritania | 45.27(34.30,59.75) | 5.89(4.46,7.77) | 43.95(28.76,67.85) | 2.57(1.68,3.97) | -2.92(-36.32,43.14) | -2.76(-2.85,-2.67) |
| Republic of the Niger | 120.94(82.07,175.65) | 4.35(2.95,6.31) | 265.45(162.40,381.72) | 2.98(1.82,4.28) | 119.49(53.19,217.27) | -1.28(-1.42,-1.13) |
| Federal Republic of Nigeria | 1017.92(774.94,1343.52) | 2.98(2.27,3.93) | 1431.58(981.67,1974.03) | 1.59(1.09,2.19) | 40.64(-1.92,109.38) | -2.38(-2.60,-2.15) |
| Democratic Republic of Sao Tome and Principe | 1.90(1.12,2.65) | 4.43(2.61,6.18) | 3.62(2.28,5.66) | 3.98(2.51,6.23) | 90.19(1.56,266.41) | -0.76(-1.37,-0.14) |
| Republic of Senegal | 168.03(130.87,214.80) | 6.09(4.74,7.78) | 231.02(167.31,312.71) | 3.58(2.59,4.85) | 37.49(-6.64,98.18) | -1.47(-1.65,-1.28) |
| Republic of Sierra Leone | 86.70(60.46,118.01) | 5.43(3.78,7.39) | 185.22(121.55,266.99) | 4.97(3.26,7.16) | 113.64(48.66,209.37) | 0.06(-0.14,0.25) |
| Togolese Republic | 81.36(60.75,105.72) | 5.93(4.43,7.71) | 154.83(98.95,212.71) | 4.60(2.94,6.32) | 90.32(22.89,178.55) | -0.76(-0.91,-0.60) |
| American Samoa | 1.52(1.23,1.91) | 7.50(6.09,9.43) | 1.20(0.90,1.55) | 6.89(5.13,8.90) | -20.68(-45.34,11.79) | -0.51(-0.68,-0.34) |
| Bermuda | 0.28(0.24,0.32) | 1.08(0.94,1.25) | 0.06(0.05,0.08) | 0.35(0.28,0.44) | -78.13(-83.89,-71.27) | -4.01(-4.26,-3.75) |
| Cook Islands | 0.51(0.36,0.70) | 6.56(4.70,9.02) | 0.22(0.15,0.31) | 3.72(2.54,5.21) | -56.75(-73.63,-30.20) | -1.41(-1.79,-1.03) |
| Greenland | 1.08(0.80,1.41) | 4.08(3.02,5.34) | 0.29(0.18,0.37) | 1.40(0.91,1.83) | -73.55(-83.40,-61.36) | -4.33(-4.69,-3.96) |
| Guam | 2.10(1.61,2.50) | 3.32(2.53,3.94) | 2.14(1.83,2.52) | 3.86(3.29,4.55) | 1.80(-19.80,43.54) | 0.57(0.24,0.90) |
| Principality of Monaco | 0.16(0.12,0.21) | 1.72(1.30,2.25) | 0.08(0.05,0.13) | 0.90(0.54,1.37) | -46.77(-69.38,-10.72) | -2.26(-2.48,-2.04) |
| Republic of Nauru | 0.91(0.64,1.32) | 22.40(15.92,32.61) | 1.03(0.72,1.50) | 22.10(15.39,32.18) | 13.59(-20.19,63.85) | -0.16(-0.62,0.31) |
| Republic of Niue | 0.07(0.05,0.11) | 8.98(6.30,13.10) | 0.05(0.04,0.06) | 8.70(6.69,11.42) | -31.61(-53.72,2.48) | -1.02(-1.29,-0.75) |
| Northern Mariana Islands | 1.83(1.16,2.56) | 7.83(4.94,10.93) | 0.72(0.56,0.93) | 4.36(3.37,5.63) | -60.82(-73.68,-37.18) | -2.28(-2.52,-2.03) |
| Republic of Palau | 0.84(0.61,1.11) | 12.09(8.71,15.96) | 0.86(0.68,1.08) | 14.59(11.60,18.38) | 1.90(-28.88,50.29) | 0.78(0.65,0.91) |
| Puerto Rico | 17.76(16.04,19.74) | 1.26(1.13,1.40) | 5.77(4.81,6.87) | 0.56(0.46,0.66) | -67.49(-73.66,-60.53) | -3.43(-3.84,-3.02) |
| Saint Kitts and Nevis | 1.36(1.21,1.55) | 7.90(7.01,8.94) | 0.33(0.23,0.45) | 1.43(1.00,1.98) | -76.13(-83.89,-65.51) | -6.23(-6.92,-5.53) |
| Republic of San Marino | 0.08(0.06,0.10) | 0.83(0.67,1.04) | 0.03(0.02,0.04) | 0.30(0.18,0.44) | -65.44(-80.61,-45.34) | -2.86(-3.13,-2.59) |
| Tokelau | 0.05(0.04,0.08) | 9.38(6.24,14.09) | 0.05(0.04,0.06) | 9.91(7.44,12.19) | -9.90(-37.32,32.92) | -0.57(-0.88,-0.26) |
| Tuvalu | 0.60(0.44,0.78) | 16.50(12.19,21.73) | 0.59(0.43,0.75) | 11.81(8.71,15.07) | -1.42(-28.63,36.68) | -1.15(-1.24,-1.05) |
| United States Virgin Islands | 1.01(0.72,1.31) | 2.54(1.83,3.32) | 0.42(0.27,0.61) | 1.80(1.17,2.62) | -58.55(-73.41,-38.47) | -1.12(-1.47,-0.77) |
| Republic of South Sudan | 115.95(79.45,164.42) | 5.02(3.44,7.12) | 142.31(90.06,208.67) | 3.95(2.50,5.80) | 22.73(-18.66,82.88) | -1.00(-1.53,-0.46) |
| Republic of Sudan | 671.23(444.49,924.70) | 8.81(5.83,12.13) | 740.70(426.66,1115.73) | 4.01(2.31,6.04) | 10.35(-36.29,82.00) | -2.58(-2.66,-2.50) |

**Abbreviations:** EAPC, estimated annual percentage change; SDI, sociodemographic Index; UI, uncertainty interval. ^a^EAPC is expressed as 95% confidence interval. ^b^Change shows the percentage change.

**Table S5** DALYs of ICH between 1990 and 2021 in the 204 countries and territories

| **location** | **Rate per 100,000 (95% UI)** | | | | | |
| --- | --- | --- | --- | --- | --- | --- |
|  | **1990** | | **2021** | | **1990-2021** | |
|  | **DALYs numbers** | **DALYs rate** | **DALYs numbers** | **DALYs rate^b^** | **Cases change^b^** | **EAPC^a^** |
| People's Republic of China | 1651705.08(1400466.00,1993851.30) | 301.33(255.49,363.75) | 1102003.76(918369.00,1298948.48) | 238.82(199.02,281.50) | -33.28(-46.71,-16.29) | -0.95(-1.23,-0.67) |
| Democratic People's Republic of Korea | 33988.70(24510.33,47089.37) | 407.50(293.86,564.56) | 50552.01(35370.30,73908.77) | 502.59(351.65,734.80) | 48.73(-0.88,137.35) | 0.38(0.15,0.61) |
| Taiwan (Province of China) | 22530.01(20808.03,24338.38) | 244.19(225.52,263.79) | 9280.22(8245.22,10283.26) | 123.00(109.28,136.30) | -58.81(-63.07,-54.21) | -1.85(-2.12,-1.59) |
| Kingdom of Cambodia | 16428.41(12869.73,20478.81) | 426.68(334.25,531.88) | 18978.64(13730.73,27114.47) | 262.02(189.57,374.35) | 15.52(-24.17,76.84) | -2.10(-2.31,-1.88) |
| Republic of Indonesia | 473667.39(405597.51,537246.88) | 606.92(519.70,688.39) | 508945.94(415644.23,665887.63) | 446.94(365.00,584.76) | 7.45(-15.51,44.54) | -0.90(-1.13,-0.67) |
| Lao People's Democratic Republic | 11245.83(8300.06,14820.70) | 727.88(537.22,959.26) | 15125.89(10774.77,20902.92) | 471.53(335.89,651.62) | 34.50(-14.03,98.39) | -1.67(-1.78,-1.56) |
| Malaysia | 25091.79(21874.79,28776.41) | 338.08(294.73,387.72) | 30446.67(26187.78,35129.64) | 219.00(188.37,252.69) | 21.34(-1.85,49.79) | -1.54(-1.92,-1.17) |
| Republic of Maldives | 512.31(421.98,671.35) | 630.41(519.25,826.11) | 593.85(465.77,731.49) | 228.26(179.03,281.16) | 15.92(-18.61,52.62) | -3.65(-4.12,-3.19) |
| Republic of the Union of Myanmar | 145897.19(112415.77,191583.74) | 849.85(654.82,1115.98) | 113235.53(87179.27,152234.53) | 503.68(387.78,677.15) | -22.39(-44.54,13.06) | -1.88(-2.16,-1.60) |
| Republic of the Philippines | 86359.81(78068.49,94794.40) | 333.19(301.20,365.73) | 174291.89(149624.18,205323.01) | 368.87(316.66,434.54) | 101.82(71.07,141.07) | 1.10(0.73,1.48) |
| Democratic Socialist Republic of Sri Lanka | 21852.38(18864.95,25211.00) | 295.52(255.12,340.94) | 13310.18(9481.86,17554.21) | 165.04(117.57,217.67) | -39.09(-58.51,-14.62) | -2.22(-2.55,-1.89) |
| Kingdom of Thailand | 61068.52(46284.92,76361.15) | 235.49(178.49,294.47) | 77547.42(59480.14,97763.92) | 365.79(280.57,461.16) | 26.98(-8.47,84.58) | 0.46(-0.28,1.21) |
| Democratic Republic of Timor-Leste | 1172.41(891.83,1519.69) | 368.34(280.19,477.44) | 1814.68(1129.67,2573.19) | 317.62(197.72,450.38) | 54.78(-6.29,126.34) | -0.51(-1.09,0.07) |
| Socialist Republic of Viet Nam | 94474.72(71886.69,123132.15) | 331.29(252.08,431.78) | 103186.86(77876.21,141112.81) | 268.78(202.85,367.58) | 9.22(-26.25,62.88) | -0.71(-0.96,-0.47) |
| Republic of Fiji | 1905.92(1591.15,2278.58) | 591.07(493.45,706.63) | 1540.25(1174.13,1980.42) | 431.77(329.13,555.15) | -19.19(-40.76,10.20) | -1.10(-1.29,-0.91) |
| Republic of Kiribati | 299.40(235.47,367.05) | 980.61(771.25,1202.20) | 511.90(379.98,672.13) | 1029.49(764.18,1351.73) | 70.98(22.15,141.06) | -0.12(-0.27,0.03) |
| Republic of the Marshall Islands | 152.39(122.06,188.89) | 888.41(711.59,1101.23) | 235.27(171.52,322.34) | 991.81(723.07,1358.87) | 54.39(12.37,108.38) | 0.60(0.45,0.76) |
| Federated States of Micronesia | 415.96(304.64,530.08) | 1038.19(760.35,1323.03) | 376.78(279.99,491.08) | 887.24(659.32,1156.40) | -9.42(-35.70,29.23) | -0.43(-0.48,-0.38) |
| Independent State of Papua New Guinea | 7203.04(4372.57,11047.02) | 435.18(264.17,667.41) | 16567.79(10093.46,24039.43) | 387.14(235.85,561.73) | 130.01(41.52,289.97) | -0.55(-0.70,-0.40) |
| Independent State of Samoa | 318.16(229.99,436.59) | 475.13(343.45,651.98) | 419.96(300.94,576.46) | 522.67(374.54,717.45) | 31.99(-9.73,93.75) | 0.35(0.20,0.50) |
| Solomon Islands | 508.63(295.34,716.09) | 396.54(230.26,558.28) | 1328.16(984.91,1752.70) | 485.58(360.09,640.80) | 161.13(74.07,349.59) | 0.83(0.73,0.93) |
| Kingdom of Tonga | 69.60(52.74,88.55) | 188.64(142.95,239.99) | 80.42(59.11,111.27) | 206.76(151.97,286.07) | 15.54(-19.56,73.27) | 0.51(0.39,0.62) |
| Republic of Vanuatu | 455.12(325.14,619.70) | 776.90(555.03,1057.84) | 992.06(725.77,1315.60) | 795.95(582.30,1055.53) | 117.98(43.50,221.58) | -0.18(-0.28,-0.08) |
| Republic of Armenia | 1604.60(1413.62,1803.41) | 111.65(98.36,125.48) | 421.25(357.04,492.68) | 39.18(33.21,45.83) | -73.75(-78.64,-68.15) | -4.45(-4.97,-3.94) |
| Republic of Azerbaijan | 8035.30(5909.28,10490.51) | 252.83(185.94,330.09) | 4663.51(3173.61,6401.35) | 110.09(74.92,151.12) | -41.96(-63.26,-13.76) | -4.12(-4.61,-3.62) |
| Georgia | 7973.24(6697.91,9318.45) | 374.51(314.61,437.70) | 1661.92(1350.29,1987.59) | 146.46(119.00,175.17) | -79.16(-83.72,-73.25) | -4.66(-6.03,-3.27) |
| Republic of Kazakhstan | 15185.54(13791.17,16703.26) | 223.68(203.14,246.04) | 9507.04(7201.38,11666.98) | 136.41(103.33,167.40) | -37.39(-52.00,-25.08) | -2.69(-3.50,-1.88) |
| Kyrgyz Republic | 5586.60(4816.42,6346.94) | 309.74(267.04,351.90) | 3302.39(2687.54,4040.90) | 121.34(98.75,148.48) | -40.89(-53.49,-25.73) | -5.01(-5.72,-4.29) |
| Mongolia | 2661.73(2022.79,3524.72) | 301.21(228.91,398.87) | 2889.75(2133.58,3832.24) | 228.96(169.05,303.63) | 8.57(-26.20,53.10) | -1.46(-2.00,-0.91) |
| Republic of Tajikistan | 5455.76(4305.26,6843.07) | 258.00(203.59,323.60) | 5984.77(4145.62,8290.52) | 143.48(99.39,198.76) | 9.70(-33.83,64.85) | -3.32(-3.90,-2.74) |
| Turkmenistan | 3085.03(2674.84,3609.45) | 200.97(174.25,235.14) | 6849.54(5317.62,8657.52) | 329.33(255.67,416.25) | 122.03(65.06,189.03) | 1.25(0.67,1.84) |
| Republic of Uzbekistan | 25835.53(23647.90,28191.81) | 300.99(275.51,328.44) | 19760.39(16933.15,22763.40) | 143.85(123.27,165.71) | -23.51(-35.34,-9.55) | -2.58(-3.23,-1.93) |
| Republic of Albania | 3323.49(2732.37,3912.73) | 233.97(192.36,275.45) | 1148.46(861.32,1473.71) | 121.14(90.85,155.44) | -65.44(-74.41,-53.22) | -2.57(-2.97,-2.17) |
| Bosnia and Herzegovina | 2138.13(1604.94,2724.33) | 112.59(84.51,143.45) | 535.31(388.70,683.05) | 53.20(38.63,67.88) | -74.96(-82.82,-65.26) | -2.91(-3.24,-2.59) |
| Republic of Bulgaria | 12001.91(10948.99,13026.39) | 403.17(367.80,437.58) | 3969.89(3376.77,4574.39) | 208.98(177.76,240.81) | -66.92(-72.68,-60.38) | -2.95(-3.22,-2.67) |
| Republic of Croatia | 2736.71(2459.69,3009.21) | 150.82(135.56,165.84) | 411.90(343.02,488.21) | 33.00(27.48,39.11) | -84.95(-87.60,-82.16) | -5.41(-5.67,-5.15) |
| Czech Republic | 3734.55(3328.99,4141.28) | 100.64(89.71,111.61) | 939.89(766.23,1145.29) | 31.83(25.95,38.78) | -74.83(-79.06,-69.46) | -3.56(-3.85,-3.28) |
| Hungary | 9087.68(8256.76,9915.43) | 245.88(223.40,268.28) | 1418.51(1204.13,1650.65) | 51.55(43.76,59.99) | -84.39(-86.91,-81.21) | -5.64(-6.12,-5.15) |
| North Macedonia | 2109.59(1773.97,2443.70) | 265.66(223.39,307.73) | 779.25(594.55,1016.80) | 101.89(77.74,132.96) | -63.06(-72.26,-51.59) | -3.35(-3.62,-3.08) |
| Montenegro | 969.57(800.35,1151.29) | 386.24(318.83,458.64) | 401.59(317.16,512.27) | 195.20(154.17,249.00) | -58.58(-68.51,-45.67) | -2.50(-3.02,-1.99) |
| Republic of Poland | 29478.33(28023.31,31018.51) | 204.07(194.00,214.74) | 8462.00(7695.53,9285.57) | 69.94(63.61,76.75) | -71.29(-73.87,-68.70) | -3.62(-4.01,-3.22) |
| Romania | 18323.65(16284.82,20315.47) | 210.93(187.46,233.86) | 5783.62(4953.51,6781.04) | 107.32(91.91,125.83) | -68.44(-74.08,-62.52) | -2.45(-2.64,-2.26) |
| Republic of Serbia | 8378.25(6884.46,10252.55) | 233.30(191.71,285.50) | 1941.84(1525.71,2465.32) | 65.51(51.47,83.17) | -76.82(-82.87,-68.46) | -4.41(-4.59,-4.23) |
| Slovak Republic | 3316.28(2755.58,3942.00) | 162.00(134.61,192.57) | 1000.65(793.75,1255.52) | 58.47(46.38,73.37) | -69.83(-76.54,-60.18) | -3.08(-3.36,-2.80) |
| Republic of Slovenia | 661.34(568.11,759.01) | 86.30(74.13,99.04) | 101.91(78.74,125.38) | 17.90(13.83,22.02) | -84.59(-87.77,-81.34) | -5.84(-6.13,-5.56) |
| Republic of Belarus | 6592.99(5680.76,7648.38) | 167.14(144.02,193.90) | 4123.42(3371.00,4999.87) | 140.55(114.91,170.43) | -37.46(-51.65,-19.64) | -1.74(-2.38,-1.10) |
| Republic of Estonia | 578.69(496.63,668.05) | 101.89(87.44,117.62) | 125.41(106.28,146.13) | 31.71(26.88,36.95) | -78.33(-82.55,-72.79) | -5.45(-6.02,-4.87) |
| Republic of Latvia | 1335.53(1199.50,1480.35) | 140.00(125.74,155.18) | 351.17(288.47,422.81) | 65.20(53.56,78.50) | -73.71(-78.25,-68.63) | -4.03(-4.59,-3.48) |
| Republic of Lithuania | 1311.15(1139.99,1491.17) | 94.10(81.82,107.02) | 334.88(289.88,391.32) | 41.59(36.01,48.61) | -74.46(-78.70,-68.93) | -2.93(-3.44,-2.43) |
| Republic of Moldova | 4296.27(3817.30,4811.18) | 246.51(219.03,276.06) | 1496.05(1277.02,1731.23) | 120.62(102.96,139.59) | -65.18(-71.27,-57.32) | -3.04(-3.41,-2.67) |
| Russian Federation | 93571.50(89884.27,97137.40) | 160.85(154.52,166.98) | 87905.54(80144.09,94230.52) | 189.14(172.44,202.75) | -6.06(-14.90,1.25) | -0.14(-0.60,0.33) |
| Ukraine | 24705.96(21632.93,28159.21) | 130.07(113.89,148.25) | 24722.83(18074.65,32154.25) | 179.35(131.12,233.26) | 0.07(-27.38,35.48) | 0.18(-0.22,0.59) |
| Brunei Darussalam | 321.44(251.89,404.37) | 260.72(204.31,327.98) | 255.21(206.17,312.65) | 125.08(101.04,153.23) | -20.61(-42.35,11.87) | -2.93(-3.45,-2.41) |
| Japan | 40074.36(37425.92,43087.92) | 89.42(83.51,96.15) | 18778.79(17127.29,20812.22) | 57.94(52.85,64.22) | -53.14(-55.52,-50.66) | -1.25(-1.46,-1.03) |
| Republic of Korea | 61038.66(50496.84,71830.55) | 290.00(239.91,341.27) | 10588.34(8880.16,13045.20) | 66.17(55.50,81.52) | -82.65(-86.17,-76.22) | -5.42(-5.78,-5.05) |
| Republic of Singapore | 1680.14(1535.69,1833.66) | 111.32(101.75,121.49) | 945.75(816.41,1090.63) | 49.16(42.44,56.69) | -43.71(-51.06,-35.95) | -3.13(-3.52,-2.74) |
| Australia | 2718.49(2446.85,3047.85) | 40.14(36.13,45.00) | 1751.95(1513.49,2041.47) | 20.21(17.46,23.55) | -35.55(-43.45,-26.45) | -2.60(-2.83,-2.38) |
| New Zealand | 590.15(541.21,644.77) | 42.72(39.18,46.67) | 345.17(296.18,403.43) | 19.16(16.44,22.40) | -41.51(-48.20,-34.19) | -2.99(-3.21,-2.77) |
| Principality of Andorra | 9.77(7.38,12.97) | 39.08(29.49,51.87) | 5.45(3.96,7.02) | 21.38(15.51,27.54) | -44.22(-62.56,-22.71) | -1.88(-2.07,-1.70) |
| Republic of Austria | 2199.03(2029.14,2392.07) | 73.25(67.59,79.68) | 631.13(551.16,722.43) | 22.36(19.53,25.60) | -71.30(-74.82,-67.79) | -4.51(-4.85,-4.17) |
| Kingdom of Belgium | 4352.67(4038.84,4690.38) | 116.99(108.55,126.07) | 853.75(756.58,967.16) | 24.38(21.60,27.61) | -80.39(-82.84,-77.82) | -4.96(-5.25,-4.65) |
| Republic of Cyprus | 189.78(146.15,234.47) | 61.71(47.52,76.24) | 134.18(107.32,162.25) | 26.74(21.39,32.34) | -29.30(-46.58,-7.67) | -3.71(-4.25,-3.17) |
| Kingdom of Denmark | 1411.11(1298.68,1537.61) | 73.96(68.07,80.59) | 395.20(344.36,454.77) | 21.67(18.88,24.94) | -71.99(-75.52,-68.01) | -4.40(-4.71,-4.08) |
| Republic of Finland | 1788.08(1597.14,1975.69) | 98.49(87.98,108.83) | 461.88(403.44,528.15) | 27.72(24.22,31.70) | -74.17(-77.59,-69.86) | -3.92(-4.26,-3.57) |
| French Republic | 17290.88(16012.43,18731.44) | 78.59(72.78,85.14) | 4513.53(3958.74,5104.50) | 22.72(19.93,25.69) | -73.90(-76.99,-70.55) | -4.24(-4.55,-3.92) |
| Federal Republic of Germany | 29324.80(26716.41,32219.89) | 98.71(89.93,108.45) | 6714.65(5910.14,7561.39) | 26.54(23.36,29.89) | -77.10(-80.02,-74.00) | -4.35(-4.74,-3.95) |
| Hellenic Republic | 6626.27(6075.32,7175.51) | 176.26(161.61,190.87) | 1879.79(1698.50,2069.58) | 67.53(61.01,74.34) | -71.63(-74.99,-67.94) | -3.21(-3.49,-2.94) |
| Republic of Iceland | 65.55(59.50,72.11) | 63.10(57.28,69.41) | 26.57(22.90,31.07) | 22.21(19.13,25.96) | -59.46(-64.92,-52.95) | -3.40(-3.75,-3.05) |
| Ireland | 732.29(672.27,798.63) | 53.38(49.01,58.22) | 254.32(211.01,305.65) | 16.25(13.49,19.53) | -65.27(-70.62,-59.95) | -3.92(-4.17,-3.67) |
| State of Israel | 1484.51(1363.51,1611.67) | 77.68(71.35,84.34) | 646.45(561.75,747.96) | 19.45(16.90,22.51) | -56.45(-62.40,-49.37) | -4.73(-4.94,-4.53) |
| Republic of Italy | 20722.48(19711.92,21840.62) | 97.07(92.33,102.31) | 5674.54(5143.49,6237.43) | 35.93(32.56,39.49) | -72.62(-74.60,-70.71) | -3.50(-3.70,-3.30) |
| Grand Duchy of Luxembourg | 206.66(190.50,223.09) | 140.04(129.08,151.17) | 48.56(41.97,56.03) | 22.01(19.03,25.40) | -76.50(-79.67,-72.58) | -6.81(-7.12,-6.50) |
| Republic of Malta | 142.78(130.70,155.48) | 103.47(94.72,112.67) | 48.38(42.23,55.22) | 36.14(31.55,41.25) | -66.12(-70.53,-61.41) | -3.28(-3.48,-3.07) |
| Kingdom of the Netherlands | 3449.84(3149.87,3782.41) | 57.21(52.24,62.73) | 1066.58(924.87,1235.56) | 20.20(17.52,23.40) | -69.08(-72.87,-65.26) | -4.01(-4.35,-3.67) |
| Kingdom of Norway | 554.32(512.31,603.95) | 34.65(32.02,37.75) | 227.28(190.97,271.45) | 12.81(10.76,15.30) | -59.00(-63.98,-54.26) | -3.68(-3.89,-3.46) |
| Portuguese Republic | 8401.25(7799.34,9007.82) | 221.90(206.00,237.92) | 1433.56(1296.62,1586.85) | 48.57(43.93,53.76) | -82.94(-84.80,-80.78) | -5.73(-6.16,-5.30) |
| Kingdom of Spain | 20778.15(19415.62,22324.11) | 140.11(130.92,150.54) | 3750.25(3345.83,4175.46) | 30.20(26.95,33.63) | -81.95(-83.91,-79.95) | -5.18(-5.42,-4.95) |
| Kingdom of Sweden | 1337.24(1205.68,1482.81) | 45.55(41.07,50.51) | 601.15(488.40,742.26) | 18.55(15.07,22.90) | -55.05(-61.60,-48.11) | -2.79(-2.93,-2.65) |
| Swiss Confederation | 1562.42(1411.38,1712.59) | 59.30(53.56,65.00) | 380.15(319.92,454.71) | 13.69(11.52,16.38) | -75.67(-79.23,-71.50) | -5.46(-5.78,-5.13) |
| United Kingdom of Great Britain and Northern Ireland | 10250.86(9622.96,11005.87) | 49.05(46.04,52.66) | 5664.50(5142.73,6233.24) | 26.04(23.64,28.66) | -44.74(-46.88,-42.64) | -2.17(-2.39,-1.95) |
| Argentine Republic | 35349.85(32616.35,38274.77) | 289.39(267.01,313.34) | 15873.66(14603.40,17312.94) | 90.59(83.34,98.80) | -55.10(-60.25,-49.75) | -3.67(-4.01,-3.33) |
| Republic of Chile | 7830.94(7219.25,8488.64) | 136.73(126.05,148.21) | 4459.74(3981.55,4943.69) | 63.03(56.27,69.87) | -43.05(-49.31,-36.84) | -2.51(-2.66,-2.36) |
| Eastern Republic of Uruguay | 2178.36(1996.87,2359.25) | 191.79(175.81,207.71) | 887.35(810.52,972.78) | 74.18(67.76,81.32) | -59.27(-63.38,-54.88) | -3.27(-3.56,-2.98) |
| Canada | 4811.57(4300.02,5394.71) | 43.28(38.68,48.53) | 3724.24(3261.87,4213.15) | 31.40(27.50,35.52) | -22.60(-31.67,-13.71) | -1.34(-1.71,-0.98) |
| United States of America | 69686.30(64896.17,75349.00) | 68.21(63.52,73.75) | 55465.54(50601.89,61055.48) | 49.83(45.46,54.86) | -20.41(-25.69,-15.61) | -1.02(-1.13,-0.90) |
| Antigua and Barbuda | 51.62(46.96,56.83) | 200.18(182.10,220.38) | 20.55(18.18,23.28) | 59.74(52.84,67.67) | -60.19(-65.88,-52.72) | -3.27(-3.80,-2.74) |
| Commonwealth of the Bahamas | 264.87(233.21,293.35) | 224.44(197.61,248.57) | 202.63(162.29,247.82) | 131.02(104.93,160.23) | -23.50(-41.06,-1.77) | -2.53(-2.80,-2.27) |
| Barbados | 161.15(147.01,177.64) | 147.64(134.68,162.75) | 72.88(58.81,93.12) | 73.78(59.54,94.27) | -54.78(-64.43,-41.38) | -2.88(-3.21,-2.54) |
| Belize | 116.54(103.49,131.69) | 159.27(141.45,179.98) | 184.99(159.43,209.18) | 97.98(84.45,110.80) | 58.74(31.58,87.05) | -2.01(-2.50,-1.52) |
| Republic of Cuba | 6977.21(6399.64,7528.89) | 142.99(131.15,154.29) | 2577.86(2226.85,2976.37) | 71.89(62.10,83.01) | -63.05(-69.07,-56.68) | -2.56(-2.85,-2.26) |
| Commonwealth of Dominica | 31.05(26.13,36.90) | 106.30(89.48,126.34) | 24.74(19.17,31.39) | 95.52(74.02,121.19) | -20.32(-40.73,8.38) | -0.61(-0.83,-0.38) |
| Dominican Republic | 7453.08(6176.27,8792.48) | 242.61(201.05,286.21) | 9229.67(7221.35,12191.51) | 202.97(158.81,268.11) | 23.84(-7.10,73.42) | 0.08(-0.28,0.45) |
| Grenada | 108.80(97.09,121.44) | 326.33(291.21,364.23) | 41.20(34.60,47.92) | 101.92(85.60,118.55) | -62.14(-69.07,-53.91) | -3.69(-4.04,-3.34) |
| Republic of Guyana | 1543.74(1282.95,1763.78) | 453.56(376.94,518.21) | 808.00(615.36,1048.70) | 260.03(198.03,337.49) | -47.66(-61.91,-31.51) | -1.96(-2.27,-1.65) |
| Republic of Haiti | 15734.59(11368.55,20913.45) | 646.37(467.02,859.12) | 22037.13(14992.90,30862.57) | 401.45(273.13,562.22) | 40.06(-1.67,95.03) | -1.25(-1.46,-1.04) |
| Jamaica | 1543.27(1298.00,1848.65) | 157.03(132.08,188.11) | 1308.83(995.83,1736.37) | 109.69(83.46,145.52) | -15.19(-41.63,22.00) | -2.19(-2.99,-1.38) |
| Saint Lucia | 123.47(114.38,134.32) | 219.47(203.31,238.76) | 73.85(61.63,87.37) | 111.74(93.25,132.19) | -40.19(-50.82,-27.35) | -2.68(-3.00,-2.35) |
| Saint Vincent and the Grenadines | 121.44(110.37,134.22) | 264.39(240.28,292.21) | 63.11(53.95,73.11) | 152.73(130.56,176.92) | -48.03(-56.99,-37.92) | -2.58(-2.90,-2.26) |
| Republic of Suriname | 547.85(381.35,651.50) | 336.42(234.18,400.07) | 519.16(408.73,653.82) | 241.86(190.42,304.59) | -5.24(-28.03,34.34) | -1.53(-1.89,-1.17) |
| Republic of Trinidad and Tobago | 910.41(840.76,994.24) | 181.59(167.70,198.31) | 698.36(538.09,900.82) | 140.30(108.10,180.97) | -23.29(-42.00,0.74) | -1.51(-1.93,-1.07) |
| Plurinational State of Bolivia | 10089.66(7501.29,13761.20) | 409.14(304.18,558.03) | 6925.98(4689.08,9678.19) | 140.94(95.42,196.95) | -31.36(-57.99,1.36) | -3.80(-4.08,-3.51) |
| Republic of Ecuador | 11969.72(11014.88,12835.79) | 290.08(266.94,311.07) | 6336.14(5156.56,7641.46) | 86.74(70.59,104.61) | -47.07(-57.52,-35.34) | -4.23(-4.65,-3.81) |
| Republic of Peru | 24432.65(19887.18,29623.97) | 275.41(224.17,333.93) | 21088.80(16183.46,27017.83) | 141.91(108.90,181.80) | -13.69(-37.91,17.77) | -2.15(-2.51,-1.79) |
| Republic of Colombia | 20581.64(19154.52,22079.34) | 146.44(136.29,157.10) | 9248.94(7785.90,10810.15) | 46.03(38.75,53.80) | -55.06(-62.49,-46.90) | -3.91(-4.39,-3.43) |
| Republic of Costa Rica | 888.45(808.86,970.96) | 69.16(62.97,75.58) | 755.10(663.27,858.35) | 39.67(34.85,45.10) | -15.01(-26.03,-2.14) | -2.47(-2.89,-2.05) |
| Republic of El Salvador | 4963.83(4193.66,5759.68) | 237.58(200.72,275.67) | 2388.21(1863.42,2988.60) | 92.14(71.89,115.30) | -51.89(-64.09,-36.74) | -3.04(-3.60,-2.47) |
| Republic of Guatemala | 9113.14(8503.91,9762.78) | 308.51(287.88,330.50) | 9930.41(8508.55,11402.56) | 145.89(125.00,167.52) | 8.97(-8.17,27.03) | -3.50(-4.04,-2.95) |
| Republic of Honduras | 6327.99(5008.05,7893.99) | 366.53(290.08,457.24) | 5972.01(3186.68,9228.95) | 135.86(72.50,209.96) | -5.63(-51.95,47.35) | -3.83(-4.18,-3.49) |
| United Mexican States | 39796.76(38315.13,41517.76) | 111.59(107.44,116.42) | 38369.63(34952.21,42357.74) | 74.49(67.85,82.23) | -3.59(-12.74,5.32) | -1.39(-1.80,-0.97) |
| Republic of Nicaragua | 2064.33(1781.61,2374.42) | 139.85(120.70,160.86) | 2000.52(1635.48,2450.42) | 70.29(57.46,86.10) | -3.09(-24.11,25.56) | -2.30(-2.54,-2.06) |
| Republic of Panama | 1280.32(1173.95,1389.67) | 126.57(116.05,137.38) | 1168.67(971.26,1387.14) | 70.81(58.85,84.05) | -8.72(-25.24,10.16) | -2.14(-2.43,-1.85) |
| Bolivarian Republic of Venezuela | 13968.88(12774.33,15176.02) | 174.41(159.50,189.48) | 12898.91(9489.63,16666.16) | 137.77(101.36,178.01) | -7.66(-32.52,19.67) | -1.37(-1.92,-0.82) |
| Federative Republic of Brazil | 195070.46(188641.87,202079.66) | 310.89(300.64,322.06) | 96765.96(92603.74,101001.90) | 113.51(108.63,118.48) | -50.39(-53.03,-47.60) | -3.51(-3.80,-3.22) |
| Republic of Paraguay | 3014.39(2465.12,3562.61) | 192.33(157.28,227.31) | 2818.19(2175.92,3613.99) | 92.11(71.12,118.12) | -6.51(-31.20,28.78) | -2.49(-2.66,-2.31) |
| People's Democratic Republic of Algeria | 29530.64(22579.85,37349.45) | 292.26(223.47,369.64) | 24747.43(17883.76,33875.89) | 145.34(105.03,198.95) | -16.20(-36.26,11.67) | -2.56(-2.78,-2.35) |
| Kingdom of Bahrain | 548.73(471.72,631.14) | 214.06(184.02,246.21) | 902.72(749.84,1067.61) | 128.31(106.58,151.74) | 64.51(31.15,109.15) | -2.12(-2.47,-1.76) |
| Arab Republic of Egypt | 97911.38(74637.84,123717.42) | 446.63(340.46,564.34) | 76509.89(55292.97,99635.85) | 181.25(130.99,236.04) | -21.86(-40.88,1.76) | -2.67(-3.02,-2.31) |
| Islamic Republic of Iran | 28847.23(24813.39,32341.01) | 132.82(114.25,148.91) | 31525.18(28990.47,34187.41) | 90.84(83.54,98.51) | 9.28(-4.69,31.12) | -0.96(-1.16,-0.77) |
| Republic of Iraq | 34184.37(27761.63,42110.83) | 475.76(386.37,586.08) | 42558.79(33209.99,56366.03) | 244.08(190.46,323.26) | 24.50(-6.23,77.11) | -2.27(-2.55,-1.98) |
| Hashemite Kingdom of Jordan | 4269.80(3533.93,5136.29) | 277.81(229.93,334.19) | 5195.19(4311.16,6196.15) | 96.78(80.31,115.42) | 21.67(-6.86,59.68) | -4.08(-4.43,-3.74) |
| State of Kuwait | 1040.13(932.50,1146.54) | 123.08(110.34,135.67) | 1585.23(1326.33,1892.08) | 74.68(62.48,89.13) | 52.41(28.71,81.17) | -2.22(-3.26,-1.15) |
| Lebanese Republic | 4045.68(3011.92,5303.59) | 350.95(261.28,460.07) | 2682.10(2225.91,3260.21) | 115.59(95.93,140.51) | -33.70(-54.66,-2.92) | -3.19(-3.74,-2.64) |
| State of Libya | 4423.04(3366.33,5672.52) | 263.24(200.35,337.61) | 6757.72(4764.82,9529.86) | 225.22(158.80,317.61) | 52.78(10.92,108.27) | -0.21(-0.54,0.12) |
| Kingdom of Morocco | 35941.75(24552.44,49314.75) | 345.70(236.16,474.33) | 22703.30(14660.15,34522.05) | 154.64(99.86,235.14) | -36.83(-56.34,1.61) | -2.81(-3.00,-2.61) |
| Palestine | 2228.20(1699.70,2947.04) | 290.32(221.46,383.98) | 3078.48(2528.91,3632.69) | 140.98(115.81,166.36) | 38.16(0.76,92.62) | -2.30(-2.62,-1.98) |
| Sultanate of Oman | 2102.60(1587.16,2752.21) | 253.42(191.29,331.71) | 2656.21(2065.37,3349.24) | 114.77(89.24,144.72) | 26.33(-10.12,80.01) | -1.90(-2.17,-1.63) |
| State of Qatar | 540.66(435.37,667.11) | 228.66(184.12,282.14) | 1347.48(1053.70,1723.46) | 81.54(63.76,104.29) | 149.23(81.23,234.21) | -3.84(-4.23,-3.45) |
| Kingdom of Saudi Arabia | 21737.61(16356.45,28635.77) | 327.11(246.14,430.92) | 47438.28(34067.74,66083.15) | 256.12(183.93,356.78) | 118.23(41.34,247.13) | -0.50(-0.71,-0.29) |
| Syrian Arab Republic | 35983.79(28402.37,44233.45) | 750.06(592.03,922.02) | 15488.64(12068.26,20324.40) | 304.52(237.28,399.60) | -56.96(-69.68,-37.39) | -2.99(-3.34,-2.64) |
| Republic of Tunisia | 6259.14(4675.63,8140.16) | 181.96(135.93,236.65) | 5166.10(3562.80,7197.05) | 118.71(81.87,165.38) | -17.46(-39.83,10.79) | -1.61(-1.69,-1.53) |
| Republic of Turkey | 72234.57(58304.25,89022.01) | 302.00(243.76,372.18) | 31123.37(25355.83,37324.63) | 97.69(79.59,117.15) | -56.91(-67.39,-44.32) | -3.89(-4.20,-3.58) |
| United Arab Emirates | 1805.87(1357.61,2454.47) | 188.92(142.02,256.77) | 3875.81(2908.23,5048.35) | 96.48(72.39,125.66) | 114.62(53.28,197.84) | -2.15(-2.35,-1.95) |
| Republic of Yemen | 16359.56(8568.84,23956.79) | 355.88(186.41,521.15) | 30511.98(19216.98,44193.69) | 221.75(139.66,321.18) | 86.51(25.29,190.54) | -1.62(-1.80,-1.44) |
| Islamic Republic of Afghanistan | 19374.17(12968.49,25779.68) | 615.14(411.76,818.52) | 50825.73(35456.20,70331.45) | 415.89(290.13,575.50) | 162.34(90.66,267.08) | -1.30(-1.75,-0.85) |
| People's Republic of Bangladesh | 200417.84(141016.95,253364.72) | 474.81(334.08,600.25) | 209770.34(134294.03,287529.83) | 304.84(195.15,417.84) | 4.67(-24.75,41.85) | -1.42(-1.68,-1.15) |
| Kingdom of Bhutan | 368.66(220.76,526.53) | 136.81(81.93,195.40) | 301.18(177.55,444.42) | 86.90(51.23,128.23) | -18.30(-49.82,35.65) | -1.98(-2.14,-1.82) |
| Republic of India | 592493.25(465629.09,725048.27) | 173.74(136.54,212.61) | 684599.65(562794.62,816229.21) | 112.33(92.34,133.93) | 15.55(-5.02,36.93) | -1.47(-1.74,-1.20) |
| Federal Democratic Republic of Nepal | 14061.82(9450.11,19310.87) | 192.49(129.36,264.35) | 14297.47(9202.00,20535.52) | 106.64(68.63,153.16) | 1.68(-32.74,51.70) | -1.93(-2.04,-1.81) |
| Islamic Republic of Pakistan | 67014.72(45360.17,85762.07) | 164.23(111.16,210.18) | 197036.61(142205.25,259873.32) | 199.22(143.78,262.75) | 194.02(117.12,324.63) | 0.26(-0.08,0.61) |
| Republic of Angola | 12076.47(8796.71,15886.41) | 308.65(224.83,406.03) | 22617.72(16007.10,29835.11) | 185.91(131.57,245.23) | 87.29(25.01,171.93) | -1.59(-1.76,-1.42) |
| Central African Republic | 3950.57(2743.07,5319.15) | 379.37(263.42,510.80) | 6914.73(4232.50,10267.86) | 316.72(193.86,470.31) | 75.03(23.31,144.10) | -0.73(-0.84,-0.63) |
| Republic of the Congo | 3320.26(2301.53,4607.12) | 350.49(242.95,486.33) | 5064.74(3389.40,7543.71) | 228.59(152.98,340.48) | 52.54(-0.52,131.46) | -1.65(-1.91,-1.39) |
| Democratic Republic of the Congo | 34352.09(23897.38,47264.62) | 239.80(166.82,329.94) | 62306.02(41628.45,88902.17) | 172.66(115.36,246.36) | 81.37(29.60,156.76) | -1.15(-1.23,-1.07) |
| Republic of Equatorial Guinea | 549.12(380.74,776.98) | 363.65(252.14,514.55) | 940.62(561.42,1509.79) | 135.22(80.71,217.05) | 71.30(5.27,174.91) | -3.82(-4.24,-3.40) |
| Gabonese Republic | 934.09(687.62,1214.24) | 242.85(178.77,315.69) | 1059.27(662.98,1590.72) | 141.32(88.45,212.22) | 13.40(-23.59,76.83) | -1.99(-2.19,-1.80) |
| Republic of Burundi | 14325.02(10628.46,19068.36) | 691.03(512.71,919.84) | 15887.34(11478.45,20663.15) | 301.34(217.71,391.92) | 10.91(-23.67,61.02) | -3.23(-3.67,-2.79) |
| Union of the Comoros | 744.13(306.24,1034.45) | 430.77(177.28,598.85) | 694.97(494.45,921.90) | 224.34(159.61,297.59) | -6.61(-39.99,109.68) | -2.90(-3.67,-2.13) |
| Republic of Djibouti | 529.63(369.62,738.73) | 302.09(210.82,421.35) | 1114.49(711.68,1655.16) | 206.09(131.61,306.08) | 110.43(35.29,228.80) | -1.43(-1.67,-1.20) |
| State of Eritrea | 7566.53(5502.04,10104.21) | 583.88(424.57,779.71) | 9881.58(6594.48,14419.16) | 352.92(235.52,514.98) | 30.60(-13.61,88.74) | -1.73(-1.87,-1.60) |
| Federal Democratic Republic of Ethiopia | 80432.90(60090.80,113788.13) | 440.25(328.91,622.83) | 75758.58(56829.71,94295.03) | 163.43(122.60,203.42) | -5.81(-41.76,30.85) | -3.75(-3.99,-3.51) |
| Republic of Kenya | 16218.33(12764.64,19552.98) | 185.19(145.76,223.27) | 34135.18(25251.53,45268.58) | 157.66(116.63,209.08) | 110.47(68.12,166.76) | -0.22(-0.53,0.10) |
| Republic of Madagascar | 41500.03(34192.21,49417.78) | 916.19(754.86,1090.99) | 74462.09(52503.01,100051.37) | 635.84(448.33,854.35) | 79.43(20.40,148.22) | -1.18(-1.28,-1.08) |
| Republic of Malawi | 12358.44(9301.76,15881.34) | 330.75(248.94,425.03) | 23717.29(16554.00,30969.43) | 289.83(202.29,378.45) | 91.91(39.53,164.63) | -0.62(-0.86,-0.37) |
| Republic of Mauritius | 1723.32(1584.39,1870.75) | 346.66(318.72,376.32) | 1269.34(1139.55,1390.67) | 278.85(250.34,305.50) | -26.34(-35.19,-16.45) | 0.32(-0.06,0.70) |
| Republic of Mozambique | 13469.69(9771.13,17133.06) | 283.99(206.01,361.22) | 42886.47(27967.98,59919.99) | 356.69(232.61,498.35) | 218.39(115.98,352.02) | 1.51(1.21,1.81) |
| Republic of Rwanda | 23259.45(17020.24,30564.27) | 848.71(621.05,1115.25) | 12182.78(8168.72,17214.57) | 214.81(144.03,303.53) | -47.62(-67.08,-20.19) | -5.75(-6.40,-5.11) |
| Republic of Seychelles | 110.85(94.35,130.03) | 355.06(302.22,416.52) | 78.93(65.11,93.69) | 205.62(169.62,244.09) | -28.80(-42.96,-11.19) | -1.45(-1.71,-1.19) |
| Federal Republic of Somalia | 14821.54(9813.62,21226.25) | 511.22(338.49,732.13) | 25985.07(14586.13,37182.18) | 313.42(175.93,448.48) | 75.32(13.90,163.73) | -1.51(-1.79,-1.23) |
| United Republic of Tanzania | 29012.17(22456.41,36882.43) | 299.44(231.77,380.67) | 43525.59(30014.26,59559.10) | 186.54(128.63,255.25) | 50.03(1.85,119.88) | -1.86(-2.03,-1.70) |
| Republic of Uganda | 17765.65(12147.01,24783.10) | 276.97(189.37,386.37) | 33506.27(22863.74,46086.60) | 194.82(132.94,267.97) | 88.60(21.76,184.81) | -2.26(-2.71,-1.80) |
| Republic of Zambia | 11840.77(9190.70,15287.64) | 390.24(302.90,503.84) | 22242.05(14542.43,31173.08) | 274.83(179.69,385.19) | 87.84(15.67,173.11) | -1.51(-1.76,-1.26) |
| Republic of Botswana | 1418.75(814.66,2071.79) | 275.61(158.26,402.47) | 1469.54(833.03,2169.42) | 137.94(78.19,203.63) | 3.58(-32.53,52.91) | -2.43(-2.72,-2.14) |
| Kingdom of Lesotho | 819.87(474.86,1164.36) | 151.95(88.01,215.79) | 2659.99(1885.70,3663.31) | 319.78(226.70,440.40) | 224.44(107.84,506.09) | 3.68(3.05,4.31) |
| Republic of Namibia | 1180.74(755.29,1573.63) | 211.14(135.06,281.40) | 1703.41(993.27,2640.58) | 162.99(95.04,252.67) | 44.27(-5.67,124.41) | -1.23(-1.71,-0.73) |
| Republic of South Africa | 68833.15(62267.08,75729.41) | 437.43(395.70,481.26) | 51842.93(46194.48,58491.14) | 213.81(190.52,241.23) | -24.68(-34.09,-13.88) | -2.99(-3.97,-2.00) |
| Kingdom of Eswatini | 663.64(444.73,891.64) | 220.21(147.57,295.87) | 1394.07(867.95,2001.71) | 273.81(170.48,393.16) | 110.06(20.17,255.59) | 1.21(0.39,2.04) |
| Republic of Zimbabwe | 3816.24(2986.72,4752.79) | 96.26(75.33,119.88) | 16973.11(11875.79,23328.15) | 267.81(187.39,368.09) | 344.76(199.87,573.36) | 4.43(3.29,5.59) |
| Republic of Benin | 4051.18(3198.46,5255.46) | 237.91(187.84,308.64) | 9602.02(7036.22,12505.99) | 183.16(134.21,238.55) | 137.02(71.04,223.36) | -0.93(-1.12,-0.75) |
| Burkina Faso | 6632.71(4859.92,8721.17) | 208.05(152.44,273.56) | 15313.72(11003.82,19975.97) | 176.95(127.15,230.82) | 130.88(64.86,210.77) | -0.42(-0.59,-0.24) |
| Republic of Cameroon | 13136.72(9556.76,16882.02) | 345.41(251.28,443.88) | 37408.63(24075.35,51708.81) | 290.15(186.73,401.06) | 184.76(98.94,308.26) | -0.55(-0.93,-0.17) |
| Republic of Cabo Verde | 445.46(353.83,531.57) | 340.67(270.60,406.52) | 385.38(275.13,499.79) | 153.77(109.78,199.42) | -13.49(-34.73,13.48) | -2.77(-3.03,-2.51) |
| Republic of Chad | 6234.19(4746.24,8138.11) | 297.00(226.11,387.70) | 17633.43(12586.78,24310.05) | 280.21(200.02,386.31) | 182.85(96.67,300.33) | -0.16(-0.39,0.07) |
| Republic of Côte d'Ivoire | 15903.63(11871.96,21050.48) | 336.23(250.99,445.04) | 31225.39(21345.94,42318.84) | 278.51(190.39,377.45) | 96.34(35.58,178.94) | -0.47(-0.65,-0.29) |
| Republic of the Gambia | 1260.56(928.88,1689.19) | 334.33(246.36,448.02) | 3027.45(2193.03,4117.77) | 302.70(219.27,411.72) | 140.17(59.97,266.79) | -0.62(-0.90,-0.34) |
| Republic of Ghana | 32807.37(24530.24,42658.48) | 571.54(427.34,743.16) | 49879.30(36375.03,65293.00) | 348.76(254.34,456.54) | 52.04(6.35,120.34) | -1.46(-1.64,-1.29) |
| Republic of Guinea | 7277.22(5632.34,9595.07) | 354.22(274.16,467.05) | 16197.93(11216.67,21910.05) | 313.48(217.08,424.03) | 122.58(55.24,215.97) | -0.14(-0.24,-0.04) |
| Republic of Guinea-Bissau | 2629.62(1901.15,3508.35) | 709.10(512.66,946.06) | 4209.63(3112.25,5614.87) | 498.90(368.84,665.44) | 60.08(14.40,133.29) | -1.09(-1.12,-1.07) |
| Republic of Liberia | 3204.36(2434.00,4230.94) | 347.32(263.82,458.60) | 6777.62(4595.49,9782.48) | 301.87(204.68,435.71) | 111.51(41.92,211.76) | -0.36(-0.61,-0.11) |
| Republic of Mali | 12739.21(9487.99,16721.95) | 426.68(317.78,560.07) | 24739.13(17802.99,32702.85) | 277.75(199.87,367.15) | 94.20(40.83,170.09) | -1.32(-1.45,-1.20) |
| Islamic Republic of Mauritania | 2914.82(2274.52,3768.78) | 379.13(295.84,490.20) | 2917.47(1972.00,4352.69) | 170.91(115.52,254.99) | 0.09(-30.18,43.76) | -2.67(-2.76,-2.59) |
| Republic of the Niger | 7979.10(5574.89,11324.63) | 286.67(200.29,406.87) | 17707.81(11436.39,24688.56) | 198.58(128.25,276.86) | 121.93(59.04,208.46) | -1.26(-1.40,-1.12) |
| Federal Republic of Nigeria | 66426.34(51770.11,85360.83) | 194.55(151.62,250.00) | 98117.02(70832.35,131832.70) | 109.09(78.76,146.58) | 47.71(6.63,111.41) | -2.21(-2.43,-2.00) |
| Democratic Republic of Sao Tome and Principe | 124.01(75.28,172.04) | 288.88(175.37,400.77) | 238.25(158.65,357.77) | 262.13(174.56,393.64) | 92.12(9.59,253.78) | -0.74(-1.33,-0.14) |
| Republic of Senegal | 10872.08(8617.43,13743.46) | 393.99(312.29,498.05) | 15167.10(11167.32,20000.06) | 235.23(173.19,310.18) | 39.51(-1.93,95.59) | -1.44(-1.62,-1.27) |
| Republic of Sierra Leone | 5603.85(4078.85,7583.72) | 350.81(255.34,474.75) | 12022.10(8145.56,17002.45) | 322.33(218.40,455.86) | 114.53(52.77,200.00) | 0.06(-0.13,0.26) |
| Togolese Republic | 5228.92(3968.07,6708.17) | 381.43(289.45,489.33) | 9865.66(6500.37,13364.14) | 293.23(193.20,397.21) | 88.67(24.55,172.48) | -0.80(-0.95,-0.66) |
| American Samoa | 96.07(78.66,118.99) | 474.87(388.78,588.15) | 75.20(57.56,95.07) | 430.34(329.41,544.02) | -21.73(-44.63,7.77) | -0.55(-0.72,-0.38) |
| Bermuda | 18.40(16.20,21.12) | 71.56(62.99,82.12) | 4.80(4.01,5.81) | 27.40(22.92,33.20) | -73.94(-79.18,-67.93) | -3.39(-3.60,-3.17) |
| Cook Islands | 32.41(24.15,43.70) | 419.95(312.97,566.27) | 14.40(10.37,19.48) | 244.39(175.98,330.65) | -55.57(-71.76,-31.41) | -1.39(-1.74,-1.04) |
| Greenland | 67.49(51.09,87.82) | 254.97(193.03,331.80) | 18.66(13.08,23.59) | 91.48(64.15,115.65) | -72.35(-81.29,-61.22) | -4.11(-4.43,-3.78) |
| Guam | 142.32(112.49,166.03) | 224.38(177.35,261.77) | 138.46(118.94,160.73) | 249.76(214.54,289.93) | -2.71(-21.17,30.23) | 0.46(0.17,0.74) |
| Principality of Monaco | 9.90(7.77,12.58) | 108.20(84.92,137.48) | 5.58(3.68,8.12) | 59.86(39.52,87.11) | -43.63(-63.95,-10.28) | -2.07(-2.26,-1.89) |
| Republic of Nauru | 55.37(40.34,80.13) | 1369.51(997.82,1981.77) | 63.11(44.54,91.62) | 1356.04(956.99,1968.49) | 13.98(-18.78,62.08) | -0.14(-0.60,0.32) |
| Republic of Niue | 4.56(3.30,6.51) | 566.31(409.60,808.73) | 3.16(2.47,4.06) | 557.04(434.32,715.35) | -30.61(-51.48,0.26) | -0.96(-1.22,-0.69) |
| Northern Mariana Islands | 112.27(74.57,152.44) | 479.39(318.40,650.92) | 45.46(36.29,57.57) | 275.71(220.09,349.18) | -59.51(-71.98,-37.69) | -2.17(-2.40,-1.94) |
| Republic of Palau | 52.16(38.13,68.27) | 747.78(546.66,978.64) | 52.25(42.21,64.85) | 887.21(716.61,1101.12) | 0.18(-28.70,43.40) | 0.71(0.59,0.84) |
| Puerto Rico | 1148.36(1045.30,1264.89) | 81.18(73.90,89.42) | 408.02(347.92,479.49) | 39.45(33.64,46.36) | -64.47(-70.14,-58.39) | -3.06(-3.40,-2.72) |
| Saint Kitts and Nevis | 80.89(72.15,91.33) | 468.06(417.52,528.50) | 20.74(15.23,27.93) | 91.24(66.99,122.90) | -74.36(-81.65,-64.12) | -5.94(-6.58,-5.30) |
| Republic of San Marino | 5.27(4.34,6.43) | 56.07(46.17,68.45) | 2.25(1.63,3.02) | 25.06(18.16,33.73) | -57.35(-71.28,-41.13) | -2.37(-2.57,-2.18) |
| Tokelau | 3.36(2.28,4.90) | 579.43(392.89,844.95) | 3.09(2.36,3.76) | 624.32(476.43,759.74) | -8.11(-34.75,33.15) | -0.54(-0.85,-0.23) |
| Tuvalu | 36.12(26.97,47.26) | 1000.73(747.38,1309.43) | 36.08(27.35,45.56) | 726.17(550.43,916.98) | -0.09(-26.38,35.99) | -1.09(-1.18,-1.00) |
| United States Virgin Islands | 61.15(44.85,77.93) | 154.38(113.22,196.74) | 26.00(17.43,37.35) | 112.42(75.37,161.48) | -57.48(-71.67,-38.29) | -0.99(-1.31,-0.67) |
| Republic of South Sudan | 7429.93(5213.38,10242.13) | 321.86(225.84,443.68) | 9106.12(5927.67,13339.63) | 253.01(164.70,370.64) | 22.56(-16.71,79.62) | -1.02(-1.54,-0.50) |
| Republic of Sudan | 42131.23(28099.34,57589.17) | 552.86(368.73,755.71) | 47343.55(28814.39,70162.95) | 256.11(155.87,379.55) | 12.37(-33.04,81.62) | -2.53(-2.61,-2.46) |

**Abbreviations:** DALYs, disability-adjusted life years; EAPC, estimated annual percentage change; SDI, sociodemographic Index; UI, uncertainty interval. ^a^EAPC is expressed as 95% confidence interval. ^b^Change shows the percentage change.
