# Supplementary material for: Global, regional, and national burden of intracerebral hemorrhage and attributable risk factors in youths and young adults, 1990–2021: a statistical analysis of incidence, mortality, and DALYs
Source: Front Neurol. 2025 Sep 9;16:1594166. doi: 10.3389/fneur.2025.1594166 (PMC12454068; doi:10.3389/fneur.2025.1594166)
Supplement: Supplementary file 2 [file Table_2.docx]

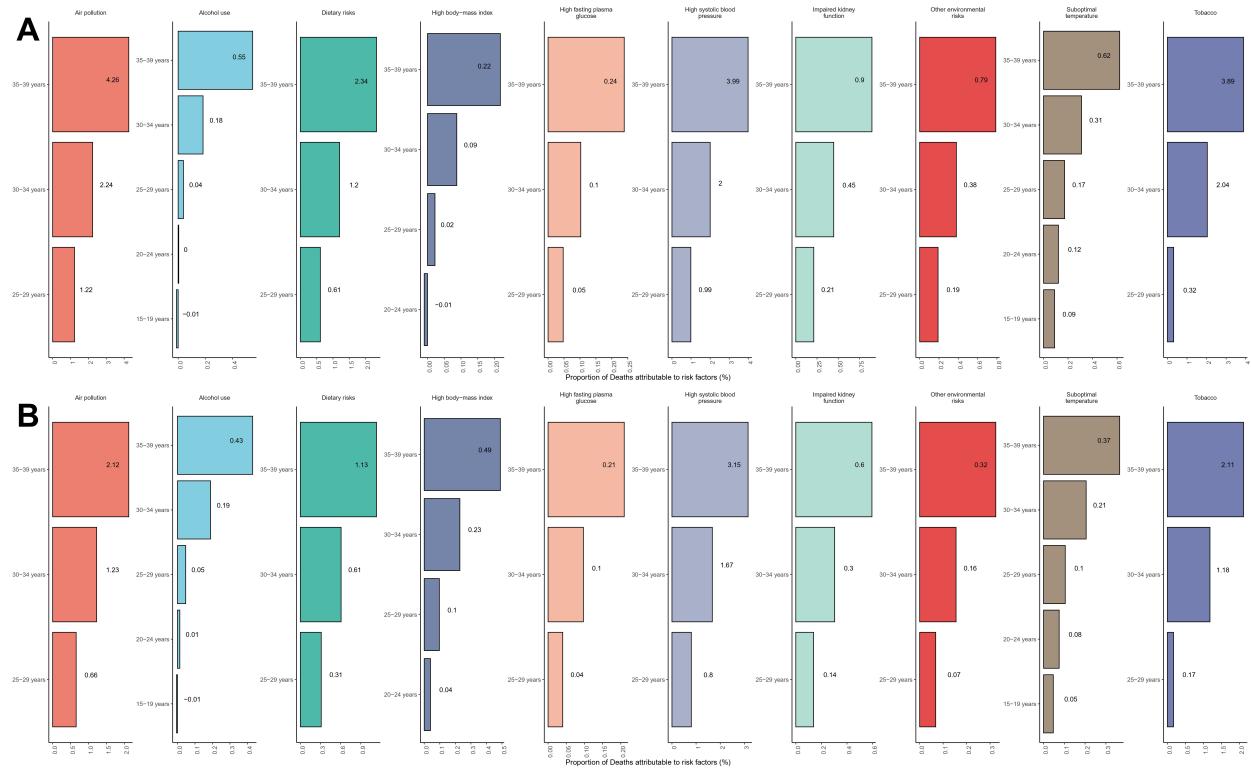


**Fig.S1** Mortality rates of global ICH attributable to risk factors across different age groups in 1990 and 2021. A, Mortality rates attributable to risk factors in 1990. B, Mortality rates attributable to risk factors in 2021.


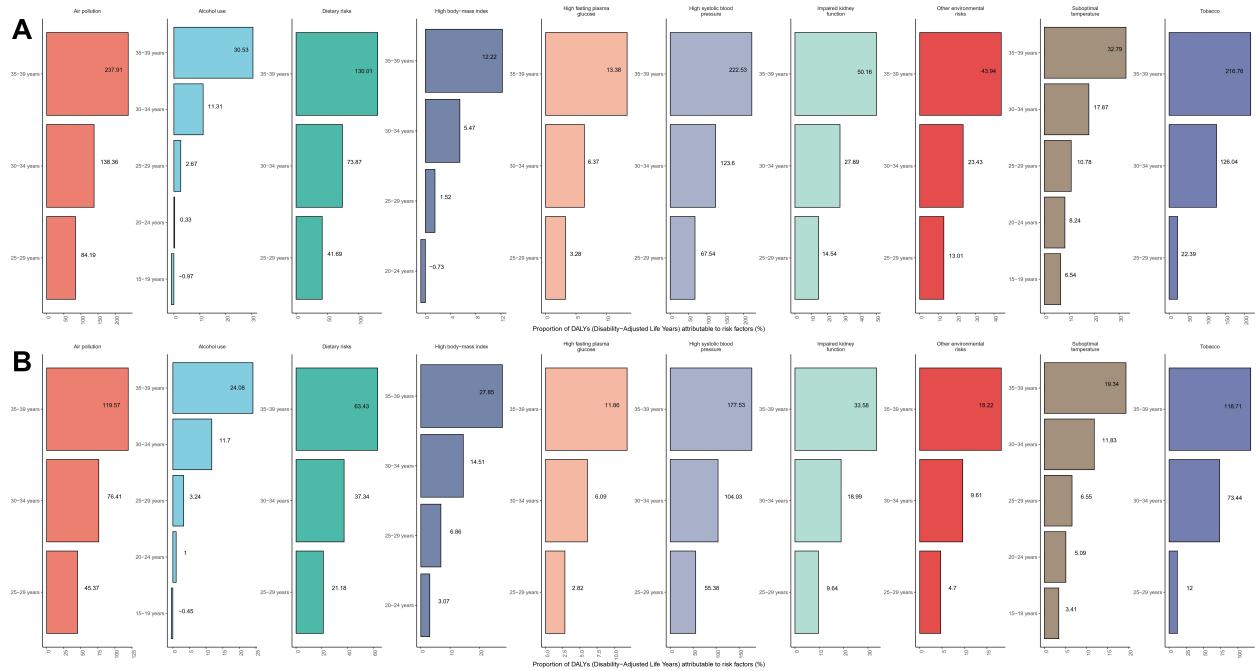


**Fig.S2** Disability-adjusted life years (DALYs) rates of global ICH attributable to risk factors across different age groups in 1990 and 2021. A, DALYs rates attributable to risk factors in 1990. B, DALYs rates attributable to risk factors in 2021.


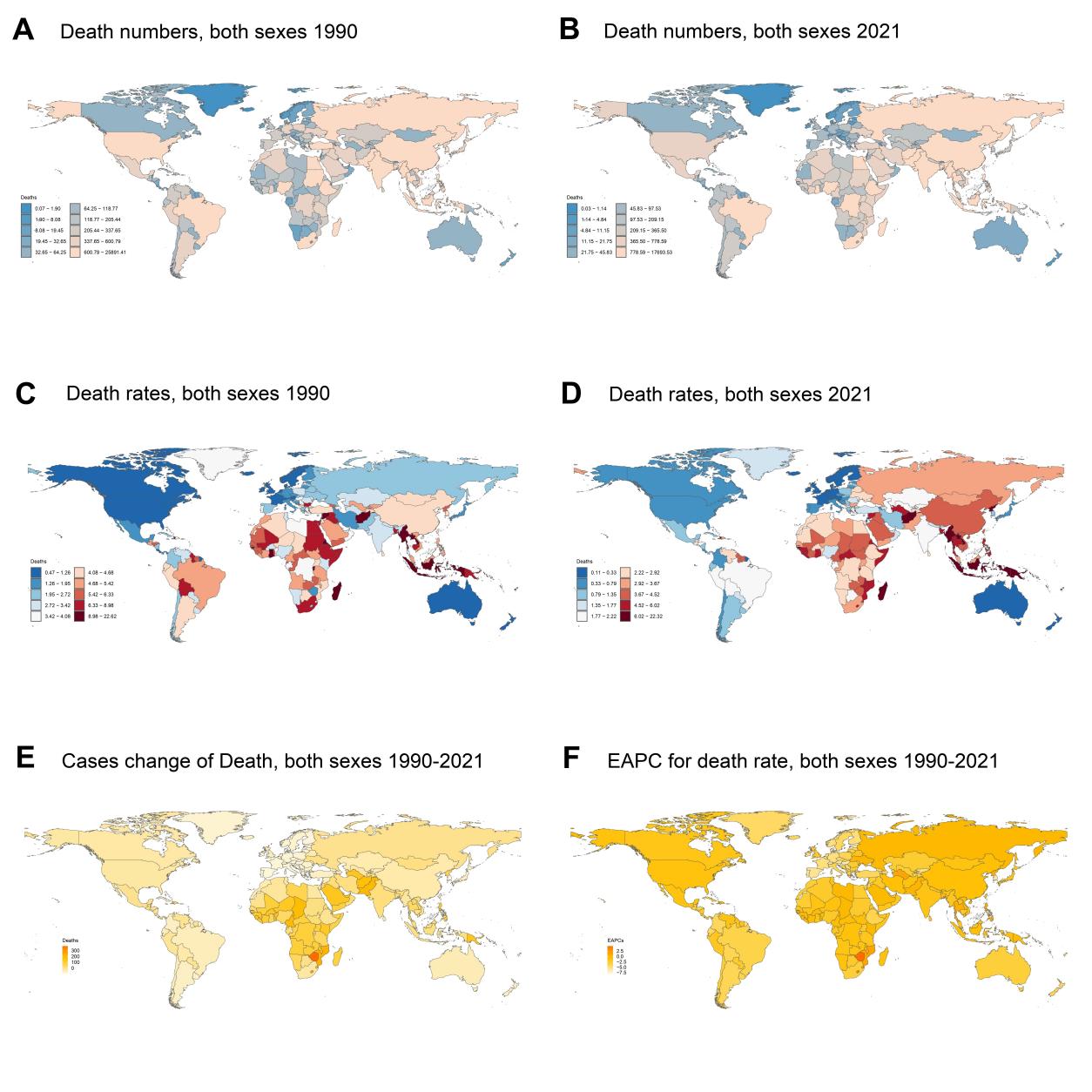


**Fig.S3** Mortality of ICH among youths and young adults across 204 countries and territories in 1990 and 2021. A, Number of deaths in 1990. B, Number of deaths in 2021. C, Mortality rate in 1990. D, Mortality rate in 2021. E, Cases change of death between 1990 and 2021. F, Estimated annual percent change (EAPC) in mortality.


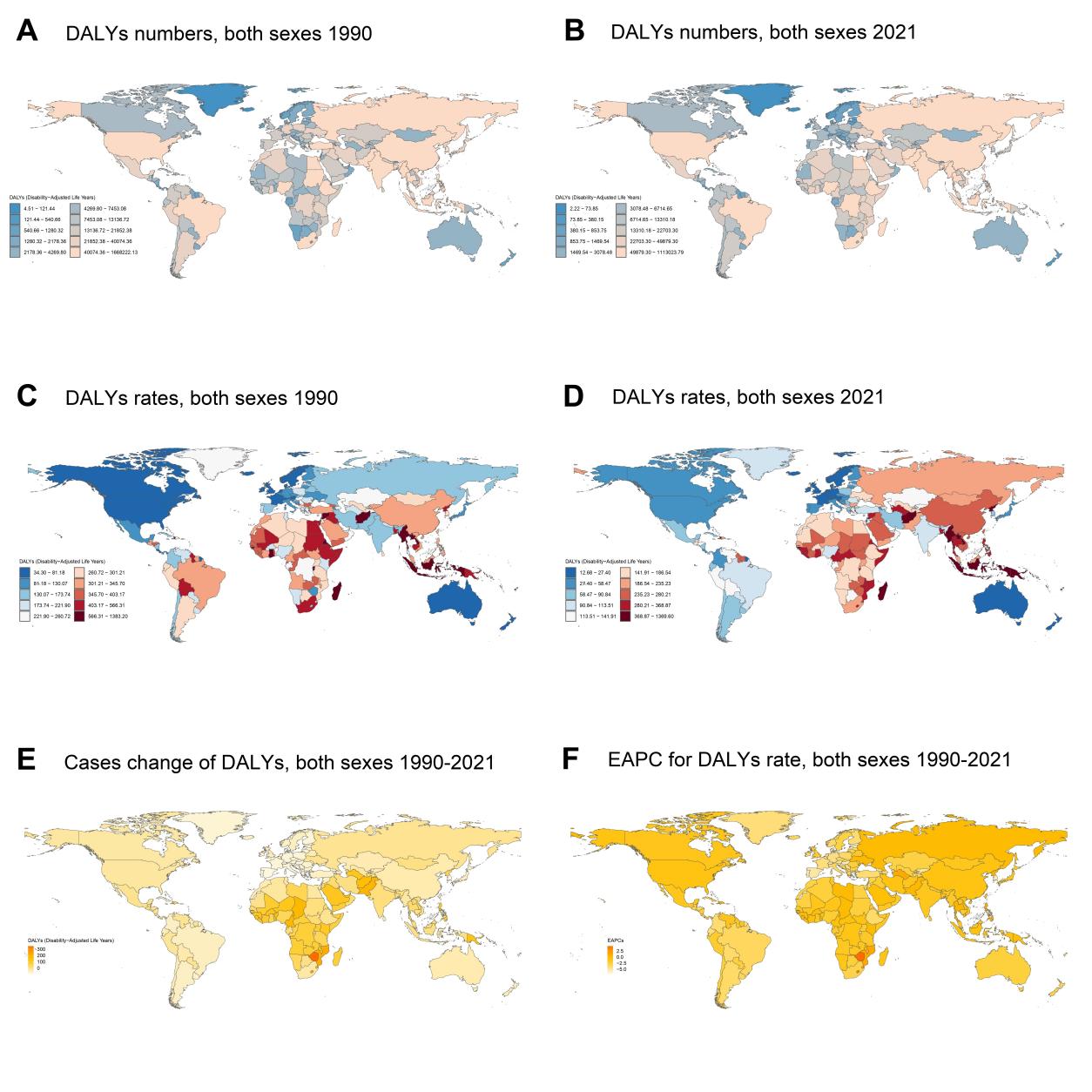


**Fig.S4** Disability-adjusted life years (DALYs) of ICH among youths and young adults across 204 countries and territories in 1990 and 2021. A, Number of DALYs in 1990. B, Number of DALYs in 2021. C, DALYs rate in 1990. D, DALYs rate in 2021. E, Cases change of DALYs between 1990 and 2021. F, Estimated annual percent change (EAPC) in DALYs.


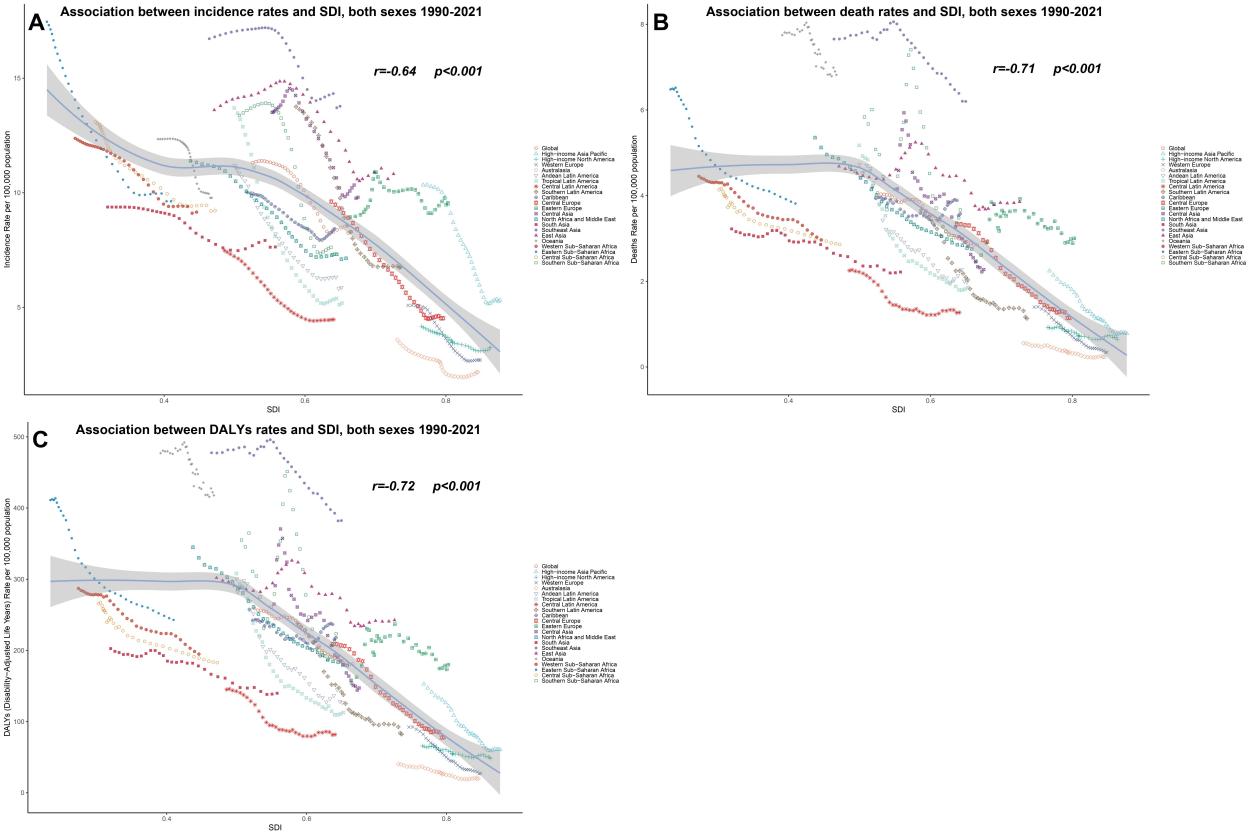


**Fig.S5** Association between incidence, mortality, and disability-adjusted life years (DALYs) rates of ICH among youths and young adults and regional Sociodemographic Index (SDI), 1990-2021. A, Incidence rate. B, Mortality rate. C, DALYs rate.
